# Prospect and challenge of detecting dynamic gene copy number increases in stem cells by whole genome sequencing

**DOI:** 10.1007/s00109-019-01792-y

**Published:** 2019-05-27

**Authors:** Ulrike Fischer, Christina Backes, Tobias Fehlmann, Valentina Galata, Andreas Keller, Eckart Meese

**Affiliations:** 1 Department of Human Genetics, Saarland University, Building 60, 66421 Homburg/Saar, Germany; 2 Clinical Bioinformatics, Saarland University, Building E2.1, 66123 Saarbrücken, Germany

**Keywords:** WGS, Gene amplification, Neural stem cell, CDK4, SHANK3

## Abstract

**Abstract:**

Gene amplification is an evolutionarily well-conserved and highly efficient mechanism to increase the amount of specific proteins. In humans, gene amplification is a hallmark of cancer and has recently been found during stem cell differentiation. Amplifications in stem cells are restricted to specific tissue areas and time windows, rendering their detection difficult. Here, we report on the performance of deep WGS sequencing (average 82-fold depth of coverage) on the BGISEQ with nanoball technology to detect amplifications in human mesenchymal and neural stem cells. As reference technology, we applied array-based comparative genomic hybridization (aCGH), fluorescence in situ hybridization (FISH), and qPCR. Using different in silico strategies for amplification detection, we analyzed the potential of WGS for amplification detection. Our results provide evidence that WGS accurately identifies changes of the copy number profiles in human stem cell differentiation. However, the identified changes are not in all cases consistent between WGS and aCGH. The results between WGS and the validation by qPCR were concordant in 83.3% of all tested 36 cases. In sum, both genome-wide techniques, aCGH and WGS, have unique advantages and specific challenges, calling for locus-specific confirmation by the low-throughput approaches qPCR or FISH.

**Key messages:**

- WGS allows for the identification of dynamic copy number changes in human stem cells.
- Less stringent threshold setting is crucial for detection of copy number increase.
- Broad knowledge of dynamic copy number is pivotal to estimate stem cell capabilities.

**Electronic supplementary material:**

The online version of this article (10.1007/s00109-019-01792-y) contains supplementary material, which is available to authorized users.

## Introduction

Gene amplification is an evolutionarily well-conserved mechanism to allow a highly efficient increase of the amount of specific proteins. Among the most prominent examples is the amplification of chorion genes in *Drosophilamelanogaster* during the developmental stages of this species [1]*.* In humans, gene amplification is known as a hallmark of genetic changes in cancer tissues with a rather extended number of tumors harboring amplified genes with very high copy numbers. In some cases, the functional role of gene amplification in cancer has already been uncovered, like the *ABCB1* gene encoding the P-glycoprotein, which is an ATP-dependent efflux pump. Upon chemotherapeutical treatment of human tumors, an amplification of *ABCB1* gene results in P-glycoprotein overexpression, and in turn, in a multidrug resistance phenotype [2]. In contrast to these high copy number amplifications often documented for malignant tumors, the amplifications are less evident in low-grade tumors. Only small fractions of the solid tissues of low-grade tumors harbor gene amplifications which consequently go frequently undetected [3].

The limited numbers of cells carrying amplified genes in a given tissue are also the reason why gene amplifications have in the past been overlooked in non-tumorous human cells. It is only in the last few years that there is increasing evidence for gene amplification in non-tumorous human cells, specifically human stem cells. We have recently identified gene amplifications in several human stem cells including neural stem cells, myoblasts, and mesenchymal stem cells, most prominent during differentiation processes [4–7]. The identification of gene amplifications in rodent stem cells showed that this mechanism appears to be conserved across the species. Two recent studies report gene amplification during trophoblast differentiation in humans [8] and also in mice [9]. As aforementioned, the fact that gene amplification in mammalian stem cells seems to be restricted both regionally, i.e., to specific tissue areas, and temporally, i.e., to specific time windows, complicates the identification of this phenomenon. To address this challenge, gene amplification was identified by single cell analysis, for example, by in situ hybridization, as previously shown for *CDK4* and *MDM2* in mesenchymal stem cells and *ERBB2* in trophoblasts [7, 8]. These approaches, however, require a prior knowledge of the amplified locus. Genome-wide amplification analyses by comparative genomic hybridization (CGH) or by next-generation sequencing (NGS), specifically whole genome sequencing (WGS), do not require a prior knowledge, but may readily miss amplifications depending on the number of cells that carry amplified genes, and the time during which the amplification occurs. This explains why there are only very few and partially contradictory reports, on a WGS-based amplification detection. A reanalysis of WGS data on human trophoblast differentiation showed amplification only by using modified cutoff criteria [9, 10]. Another WGS-based study failed to detect gene amplifications in human trophoblast cells [11] that harbor an *ERBB2* amplification detected by fluorescence in situ hybridization as mentioned above [8]. These results underline the challenges associated with the use of WGS for the detection of amplifications that are masked by their time and space-limited appearance.

Here, we evaluated the potential as well as the limitations of WGS to detect dynamic copy number changes in human mesenchymal stem cells (hMSCs) and human neural stem cells (hNSCs). Using aCGH, qPCR, and FISH as reference methods and different in silico strategies for detection of dynamic copy number increases, we demonstrate amplification detection by WGS. In contrast to previous studies, we apply a novel sequencing method, developed by BGI that has previously already shown very promising results in the accurate quantification of RNAs [12].

## Materials and methods

### Cell culture and differentiation

GIBCO Human neural stem cells (H9 hESC-derived), further named NSC, were cultured on CELLStart™-coated culture ware with complete StemPro NSC SFM medium as described in the manufacturers’ instructions with EGF/bFGF. For spontaneous differentiation of NSCs, these cells were plated at 2.5 × 10^4^ cells/cm^2^ on CELLStart tissue culture plates in StemPro NSC SFM medium without bFGF and EGF.

### DNA isolation

Cells were harvested and DNA was isolated using chloroform/NaCl method. In brief, the cell pellet was digested with proteinase K at 55 °C overnight (> 12 h) and chloroform extracted for 1 h at room temperature.

### Sequencing

The DNA samples were RNase treated and sent to BGI for whole genome sequencing 50-fold BGI SEQ 500 service. Read length for all samples was 100 bp.

### Bioinformatics analysis

First, we merged the technical replicates per sample by simply concatenating the respective fastq files. These served as input for the bcbio-nextgen pipeline (https://github.com/chapmanb/bcbio-nextgen). This pipeline performed read mapping with bwa 0.7.15-r1140 [13] against GRCh37/hg19, and marking duplicates, realigning, recalibrating, and variant calling with GATK 3.8-0-ge9d806836 [14]. Read quality was assessed with MultiQC v1.2 [15]. We used mosdepth 0.1.7 [16] to determine the proportion of the genome covered at a certain depth.

For detecting amplification differences in our samples, we used Control-FREEC 11.0 [17] with the bam files generated by the bcbio-nextgen pipeline with the following parameters: ploidy = 2, breakPointThreshold = 0.1, window = 25,000, mateOrientation = FR, PB as control sample. In addition, we set the options for GC content normalization and to exclude low mappability regions. The output of Control-FREEC served as input for the R package ggplot2 [18] for visualizing the log_2_ ratios along the chromosomes.

### qPCR analysis

TaqMan Copy Number Assays for genes *CDK4* (Hs00957586_cn), *DIABLO* (Hs00949671_cn), *GINS2* (Hs05472641_cn), *RAB20* (Hs02953107_cn), *EGFR* (Hs01463609_cn), *MAPK8IP2* (Hs00526226_cn), *PPP6R2* (Hs02084609_cn), *SHANK3* (Hs04081743_cn), and *FAM19A5* (Hs05567853_cn) were performed following manufacturers’ instructions. We used the *RNaseP* TaqMan Copy Number reference assay for relative quantitation of copy number of target genes. DNA from human normal blood lymphocytes (PB) was used as control standard for normal diploid copy number.

TaqMan assays were run in four technical replicates and results were analyzed using StepOne™ Software v2.0 and CopyCaller™ software.

### Fluorescence in situ hybridization

Human mesenchymal stem cells were grown on glass slides and fixed in ice-cold methanol for 15 min.

BAC clones were taken from the RP-11 (http://www.chori.org/bacpac/) libraries of the Welcome Trust Sanger Institute and available from SourceBioSciences, Germany [19]. Labeling, hybridization, and post-hybridization washes were as described previously [4].

## Results

WGS was done by a BGISEQ-500RS on different human stem cells that harbor gene amplifications as shown by independent methods. Stem cells included hMSCs that were previously analyzed by aCGH, hNSCs that were analyzed using qPCR, and peripheral blood lymphocytes (PB) from a healthy female donor as control. The stem cells were grown under conditions that were previously shown to favor the development of gene amplifications. In detail, the hMSCs were harvested after 4 days of culturing and hNSCs were analyzed prior to culturing (0h_1, 0h_2), after 2 days of culturing in presence of growth factors EGF/bFGF (2d_C), and after 2 days of culturing without growth factors EGF/bFGF (2d_Diff). The lack of the growth factors EGF/bFGF triggers differentiation processes of the stem cells. A summary of the analyzed primary cells and their condition of culturing is given in Table 1 [7].

**Table 1:** Overview of the analyzed primary cells and their culturing conditions

Sample name	Cell type	Time of DNA isolation	Source
hMSC	Human mesenchymal stem cells	Upon 4 days of culturing	Lonza (Walkersville Inc. USA)
hNSC 0h_1/0h_2	Human neural stem cells	Upon thawing	GIBCO
hNSC 2d_C	Human neural stem cells	Upon 2 days of culturing with EGF/bFGF after 1 passaging	GIBCO
2d_Diff	Human neural stem cells	Upon 2 days of culturing without EGF/bFGF after 1 passaging	GIBCO
PB	Peripheral blood lymphocytes	After donation	Female donor

### Quality assessment for WGS data

**Fig. 1:**
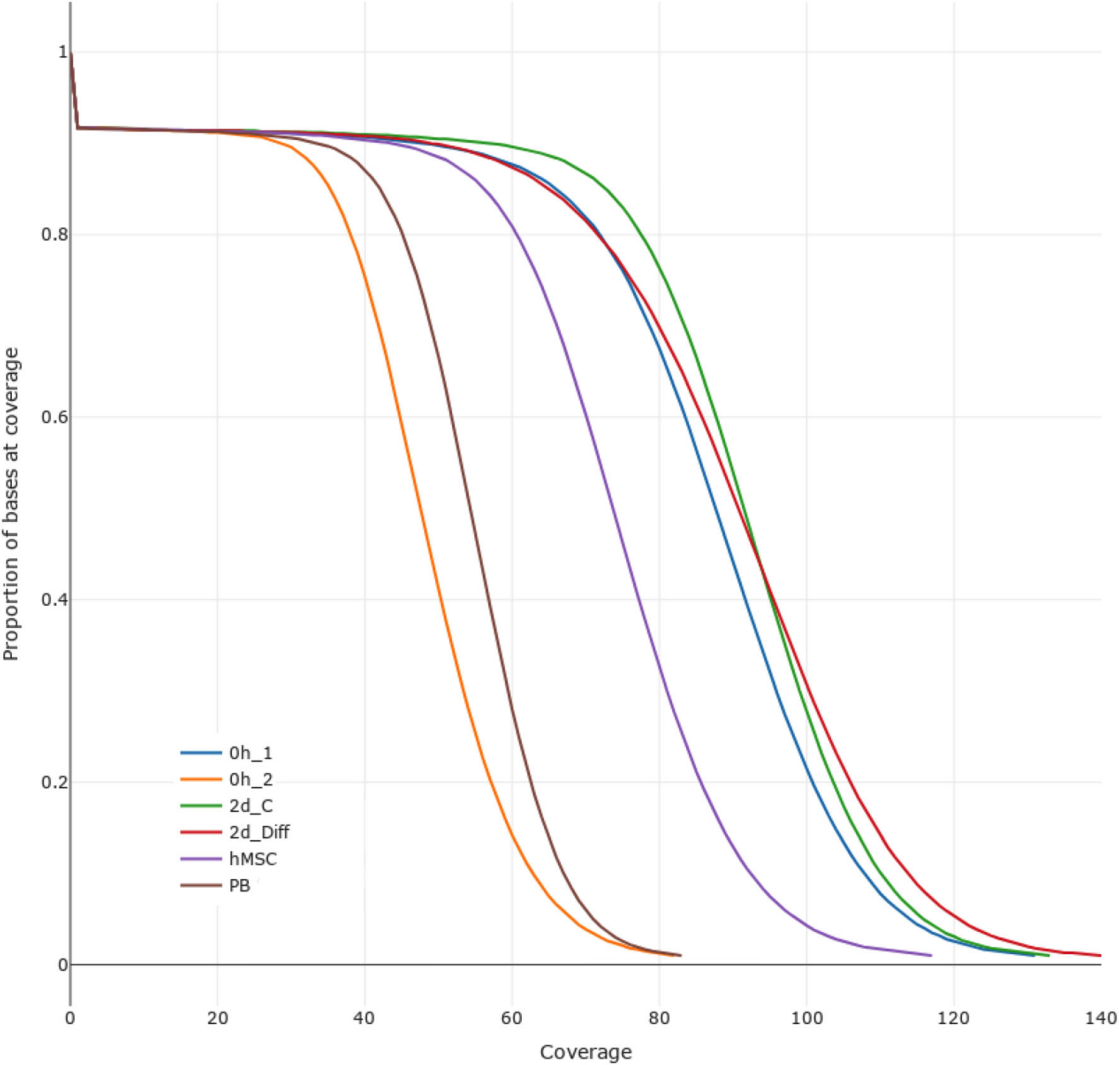
Distribution of the genome coverage. Approximately 80% of the genome is covered with a depth of at least 38x and about 90% of the genome with a coverage of about 30x. 0h_1: human neural stem cells analyzed directly upon thawing; 0h_2: biological replicate of the experiment; 2d_C: human neural stem analyzed after 2 days of culturing with EGF/bFGF; 2d_Diff: human neural stem cells analyzed after 2 days of culturing without EGF/bFGF; human mesenchymal stem cells analyzed after 4 days of culturing; PB: peripheral blood lymphocytes analyzed after blood drawing

To assess the quality of the generated reads, we used the tool MultiQC. This tool summarizes different metrics and alignment statistics to facilitate quality control (Table 2). The sequencing runs were generated between 1.8 and 2.9 billion reads per sample, of which more than 99.2% could be mapped to the human genome (hg19). The average depth ranges from 60 for 0h_2 to 96 for 2d_Diff. A distribution of the coverage against the proportion of the genome that is covered is depicted in Fig. 1. As can be seen, we cover about 80% of the bases of the genome with a depth of at least  38x and about 90% of the genome with a coverage of about  30x. Since the samples 0h_1, 0h_2, 2d_C, and 2d_Diff stem from the same stem cells, we compared how well variant calling matched in these samples. Considering these four samples, we detected a total of 4,500,581 variants that passed quality filtering and where all of these samples had an annotated genotype. Of these variants, 4,371,447 (97%) had the same annotated genotype (heterozygous or homozygous variant). Combining these results with the variants of hMSC (passing filters, having an annotated genotype), we still detect 2,868,360 variants of which 1,714,982 (60%) have the same genotype for these five samples. The differences between hMSC and the other samples are expected because of the different stem cell types. In summary, the mapping metrics and variant concordance analysis illustrate that our sequencing data are high quality. An overview of read numbers and mapping statistics is displayed in Table 2.

**Table 2:** Overview of read numbers and mapping statistics generated by the tool MultiQC

Sample	Total reads	Reads aligned	% mapped reads	Depth
0h_1	2813.22 M	2805.12 M	99.7	90.60
0h_2	1856.53 M	1854.57 M	99.9	60.12
2d_C	2953.84 M	2945.18 M	99.7	95.21
2d_Diff	2972.57 M	2961.18 M	99.6	95.79
hMSC	2617.69 M	2595.81 M	99.2	84.04
PB	2050.34 M	2047.72 M	99.9	66.26

### Analyzing copy number variants

To analyze if we can detect copy number variants in the four samples stemming from neural stem cells incubated at different conditions and mesenchymal stem cells, we used the tool Control-FREEC. To make the samples comparable, we used the PB sample as control. Control-FREEC normalized the read counts for each sample against the control sample and computed the copy number profiles.

As previously reported by [9, 10], the threshold setting is decisive to identify amplified genes within WGS-derived sequencing data. In the following, we refer to log_2_ ratio values > 0.05 as copy number gains, values ≥ 0.1 as amplifications, values < − 0.05 as copy number losses, and values ≤ − 0.1 as under-replications. To verify amplifications identified by WGS, we used two independent methods with aCGH as a method that does not require a prior knowledge of the amplified locus, but covers the entire genome, and qPCR for the analysis of single amplified loci.

The complete WGS and aCGH data set for all chromosomes of hMSCs and hNSCs are provided in the Supplementary Material.

### aCGH versus WGS

**Fig. 2:**
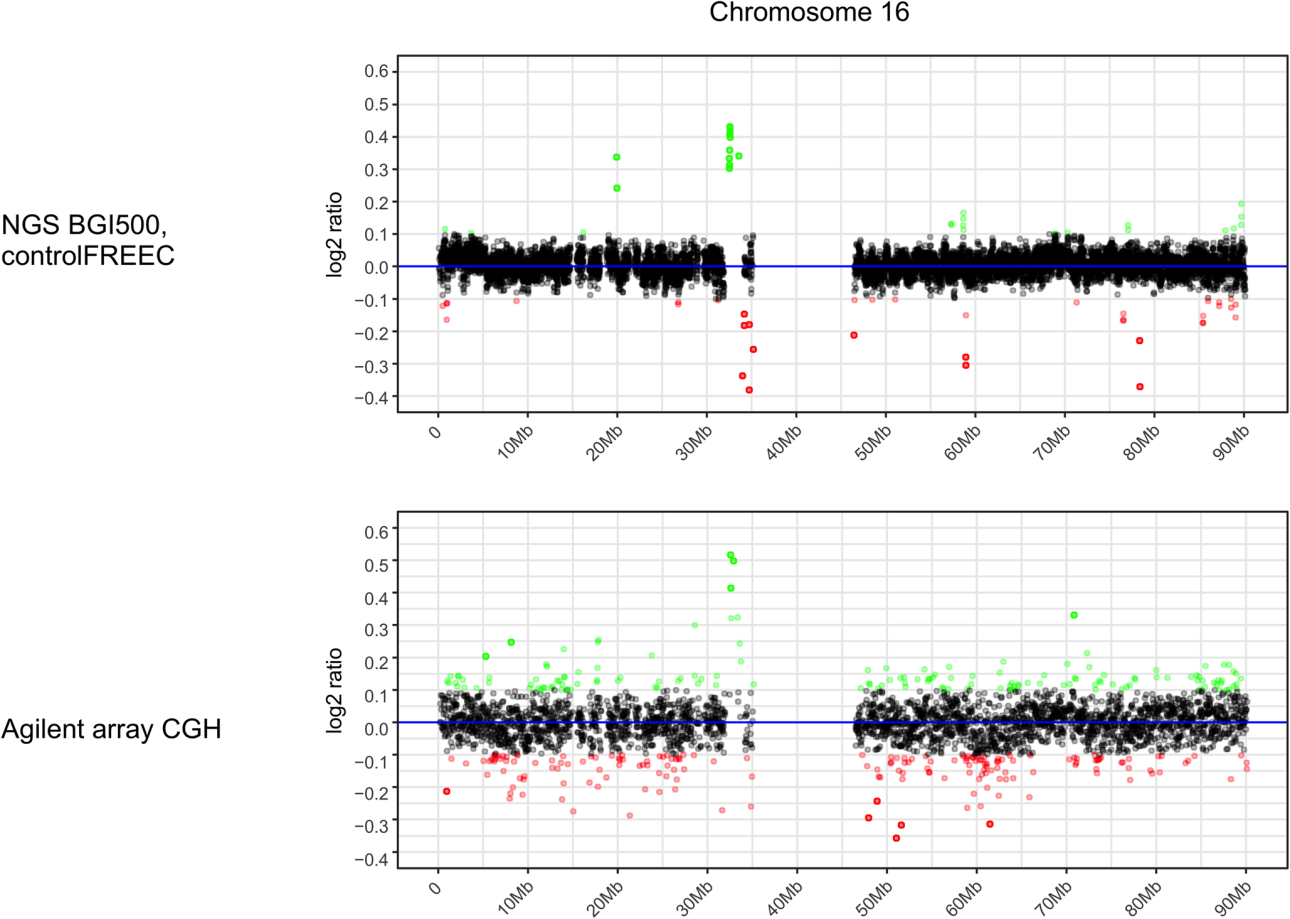
Comparative analysis of copy numbers on chromosome 16 isolated from human mesenchymal stem cells by aCGH and by WGS. The aCGH have previously been generated by using Agilent SurePrint G3 Human CGH microarrays as documented by Altmayer et al. [7]. The CGH analysis shows the array signal intensities as log_2_ ratio values on the *y*-axis and the chromosomal position in megabases on the *x*-axis scale The WGS data were generated by a BGISEQ-500RS. The log_2_ ratio values of normalized copy numbers are shown on the *y*-axis, and the chromosomal position megabases (Mb) on the *x*-axis. Significantly amplified regions are shown in green and under-replicated regions in red. The array and the WGS results are displayed in a way that corresponding chromosomal regions are arranged directly beneath each other. A copy number increase at 32 Mb is detected by aCGH and WGS

**Fig. 3:**
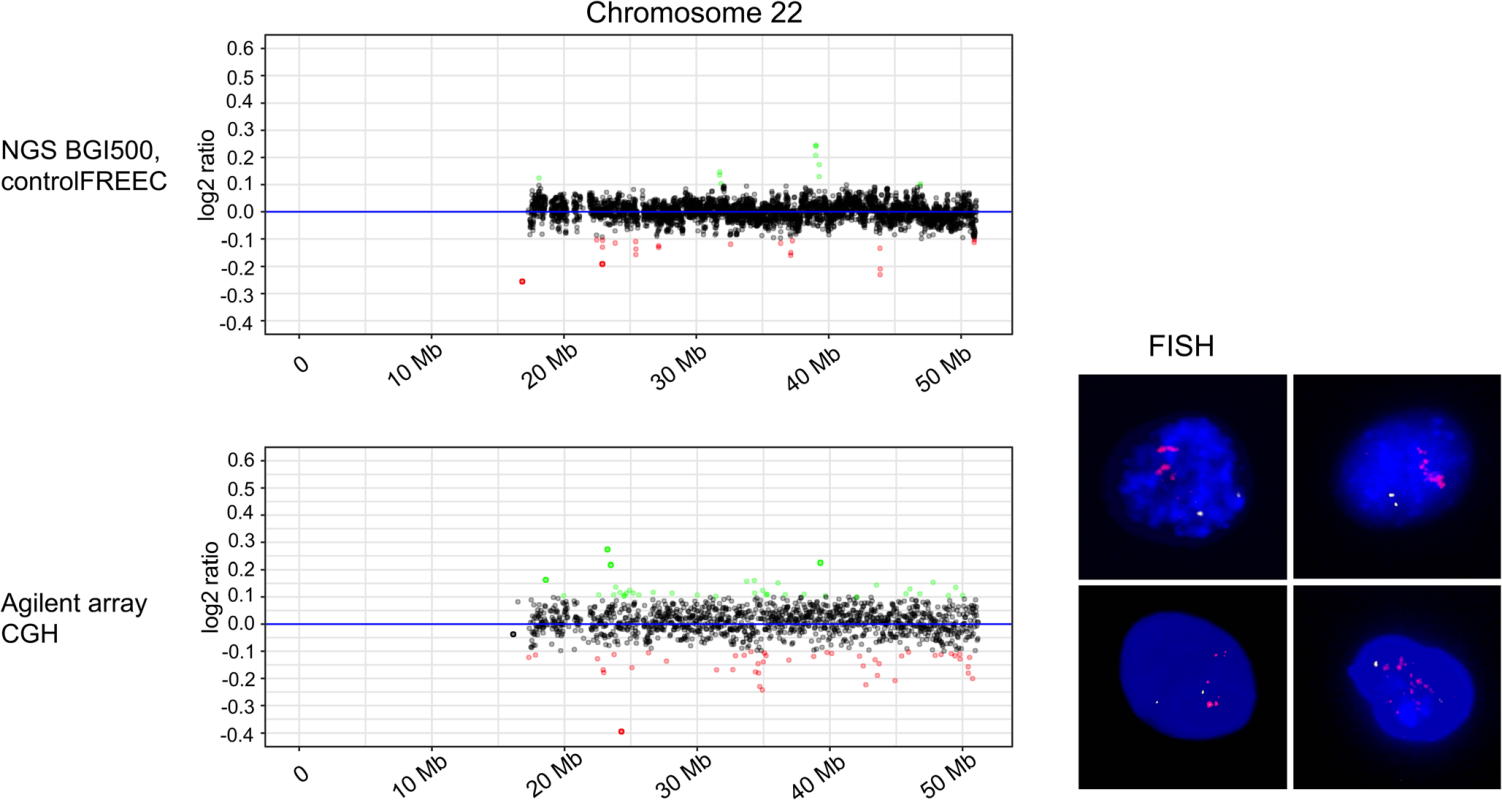
Comparative analysis of copy numbers on chromosome 22 isolated from human mesenchymal stem cells by aCGH and WGS. aCGH and WGS analyses have been performed as described in Fig. 2. The CGH analysis shows the array signal intensities as log_2_ ratio on the *y*-axis, and the chromosomal position in megabases on the *x*-axis scale. Significantly amplified regions are shown in green and under-replicated regions in red. The array and the WGS results are displayed in a way that corresponding chromosomal regions are directly arranged beneath each other. A copy number increase at 24.3 Mb is detected by aCGH but not by WGS. As shown on the right-hand side, fluorescence in situ hybridization identified an amplification of the *CABIN1*gene, which maps in the region at 24.3 Mb. *CABIN1* gene is represented by BAC probe RP11-297B9 with red fluorescence signals; BAC RP11-81J11 served as control with green fluorescence signals

The comparison of the WGS data with aCGH was done with human mesenchymal stem cells that were previously analyzed by aCGH using Agilent-021529 SurePrint G3 Human CGH Microarray Kit [hg19:GRCh37:Feb2009]. For direct comparison, we used the identical DNA preparation as previously used for aCGH experiment. As example chromosome 16 in Fig. 2, the WGS and the array techniques showed similarities, but also dissimilarities, with regard to copy number changes. Both analyses clearly indicated that hMSCs have a copy number increase at 32 Mb on chromosome 16. Other copy number increases are indicated either by aCGH or by a WGS, but not by both methods, as also documented in Fig. 3. Here, single-cell analysis by fluorescence in situ hybridization of gene *CABIN1* demonstrated an amplification at 24.3 Mb of chromosome 22 in hMSCs. At the same position, we found evidence for an amplification by using aCGH. WGS analysis, however, failed to detect this amplification. As for the overall picture, and as already shown for the data of chromosome 16, several copy number changes on chromosome 22 of hMSCs were only found either by aCGH or by a WGS. The complete data set for all chromosomes of hMSCs are provided in the Supplementary Material.

### qPCR versus WGS

For the verification of single-gene amplification, we used qPCR of genes that were previously shown to be amplified in hNSCs under specific culture conditions (Table 1). The conditions include hNSCs that were cultured for 2 days supplemented by medium containing EGF/bFGF (2d_C) and for 2 days supplemented by medium without EGF/bFGF (2d_Diff). In addition, we included two biological replicates of hNSCs without culturing (0h_1, 0h_2). As for the tested genes, we analyzed the copy numbers of the genes *CDK4* and *DIABLO* on chromosome 12 by both PCR and WGS. Likewise, we analyzed the copy numbers of the genes *FAM19A5*, *PPP6R2*, *MAPK8IP2*, and *SHANK3* on chromosome 22, *GINS2* on chromosome 16, *RAB20* on chromosome 13, and *EGFR* on chromosome 7 by both approaches. The qPCR experiments were done in four technical replicates.

**Fig. 4:**
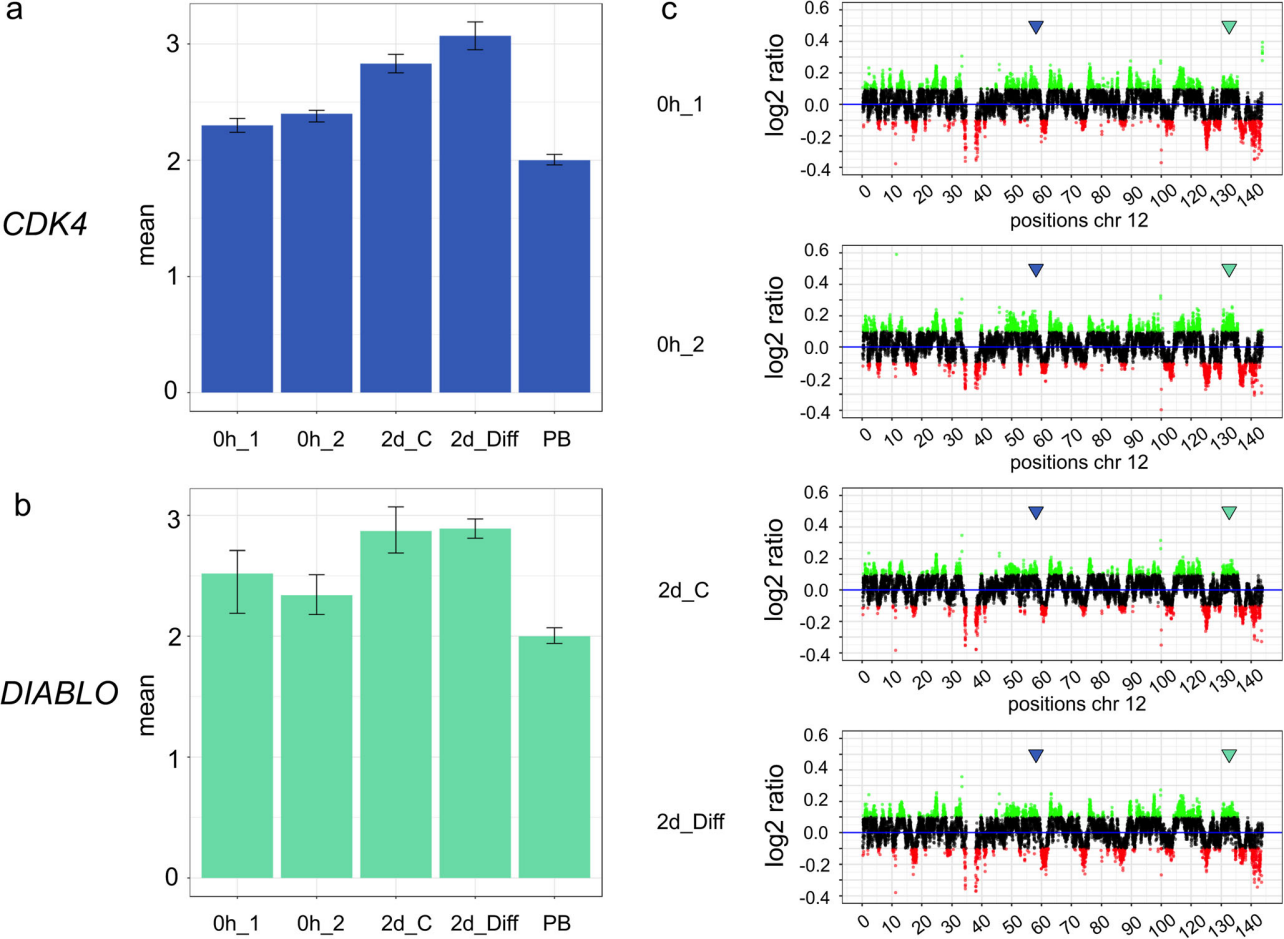
Comparative analysis of copy numbers of the genes *CDK4* and *DIABLO* on chromosome 12, isolated from human neural stem cells (hNSCs) by qPCR and WGS. hNSCs were analyzed prior to culturing (0h_1 and 0h_2), after 2 days of culturing in presence of growth factors EGF/bFGF (2d_C), and after 2 days of culturing without growth factors EGF/bFGF (2d_Diff). The qPCR analysis of *CDK4* (**a**) and *DIABLO* (**b**) shows the copy numbers on the *y*-axis and the growth conditions on the *x*-axis. In addition to the hNSCs, peripheral blood lymphocytes were used as a control with normal copy numbers. The WGS analysis (**c**) shows the log_2_ ratio values of normalized copy numbers on the *y*-axis and the chromosomal position in megabases (Mb) on the *x*-axis. Significantly amplified regions are shown in green and under-replicated regions in red. The chromosomal positions of the genes *CDK4* and *DIABLO* are indicated by arrows in blue and green, respectively

**Fig. 5:**
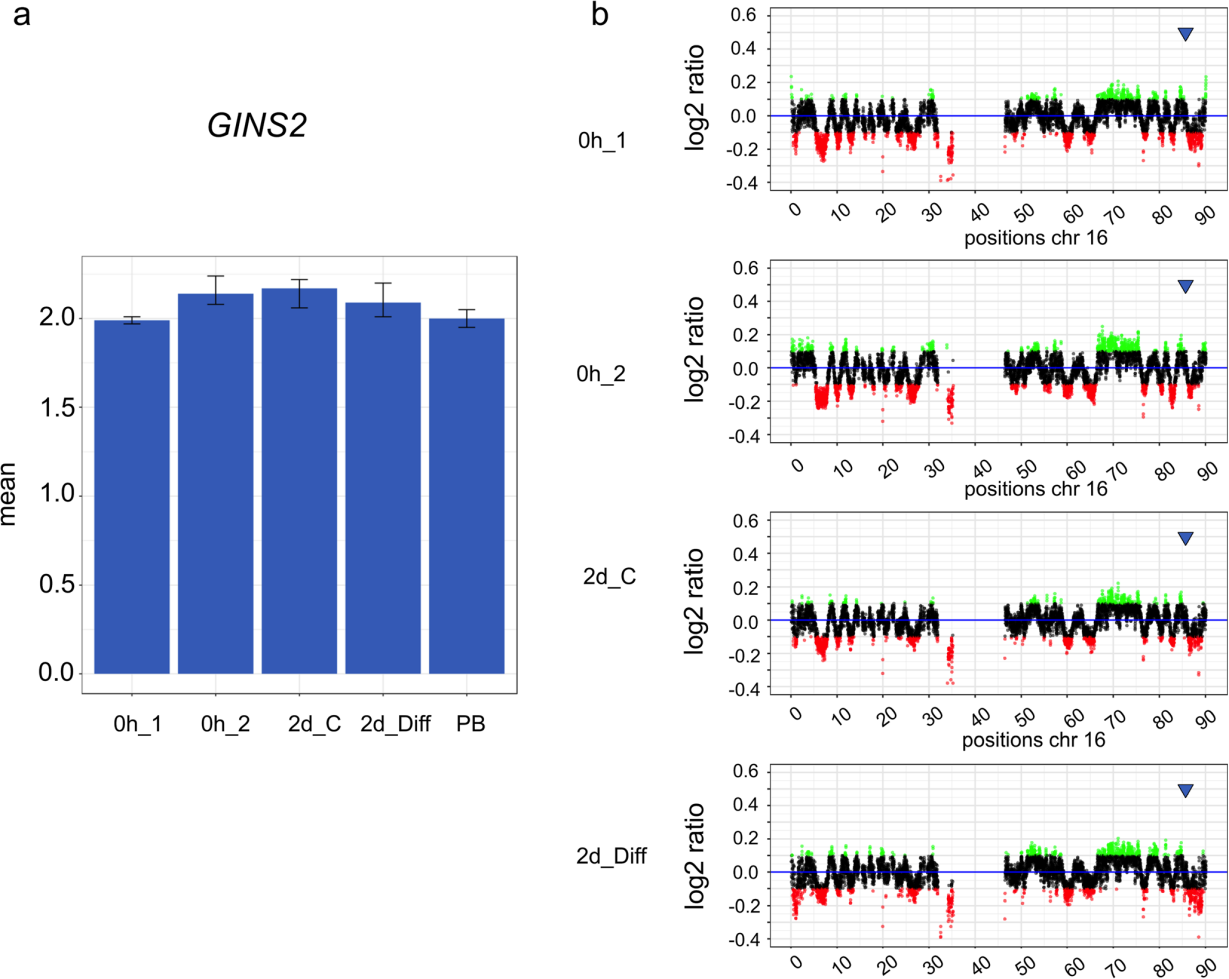
**a**, **b** Comparative analysis of copy numbers of the gene *GINS2* on chromosome 16 isolated from human neuronal stem cells (hNSCs) by qPCR (a) and WGS (b). The culture conditions of hNSCs, the source of the peripheral blood lymphocytes, and data display were the same as in Fig. 4. The chromosomal position of gene *GINS2* is indicated by a blue arrow

qPCR detected elevated copy numbers for the gene *CDK4* for hNSCs that were analyzed prior to culturing, after 2 days of culturing in presence of growth factors EGF/bFGF, and after 2 days of culturing without growth factors EGF/bFGF (Fig. 4a). Likewise, qPCR detected elevated copy numbers of the gene *DIABLO* for all growth conditions (Fig. 4b). The highest numbers of gene copies were found for *CDK4* and *DIABLO* after 2 days of culturing without EGF/bFGF, i.e., after 2 days of the differentiation process. A similar high copy number for both genes was found after 2 days of culturing with EGF/bFGF. Since there was no medium replacement during these 2 days, the EGF/bFGF was depleted, thereby triggering differentiation. WGS data indicate elevated copy numbers for all four conditions for both loci at which the genes *CDK4* and *DIABLO* map (Fig. 4c). However, comparable copy number differences between the four culture conditions were not revealed by WGSs, but only by qPCR (Table 3). Besides the chromosome region that contains the genes *CDK4* and *DIABLO*, there were multiple other regions on chromosome 12 at which WGS identified elevated copy numbers. WGS was also able to detect rather low copy number levels as shown for the gene *GINS2*. As shown in Fig. 5, the low copy number elevation of *GINS2* that was detected by qPCR was also found by WGS in hNSCs which were analyzed immediately upon thawing and after 2 days of culturing with EGF/bFGF.

**Table 3:**
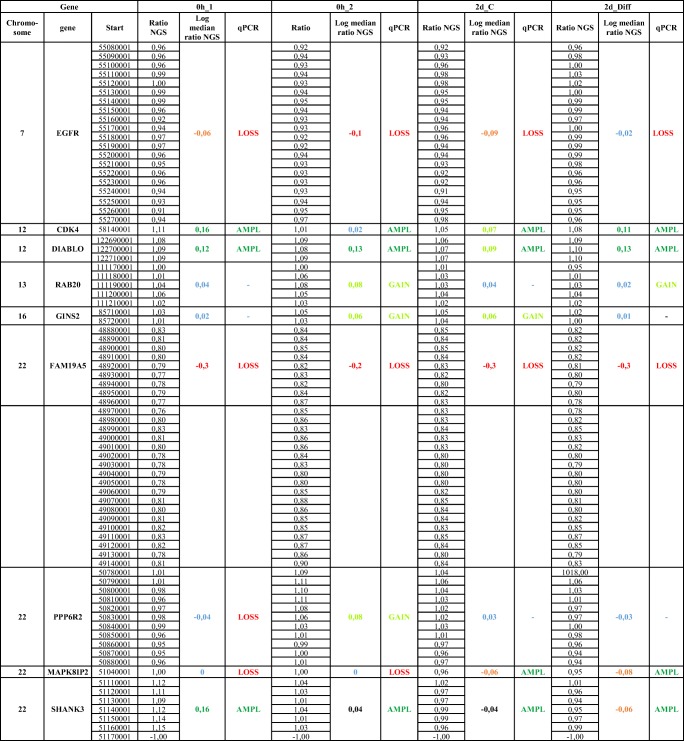
Results of ControlFREEC analysis of genes analyzed by qPCR

The copy numbers are indicated by log_2_ ratio values 10 kb encompassing regions of the genes that have been analyzed by qPCR. Regions with copy number gains (> 0.05) are indicated by light green, regions with amplifications (≥ 0.1) by dark green, regions with reduced copy number (< − 0.05) by orange, and regions with under-replication by red (≤ − 0.1). Genes that are inside of the corridor between − 0.05 and 0.05 are colored in blue

**Fig. 6:**
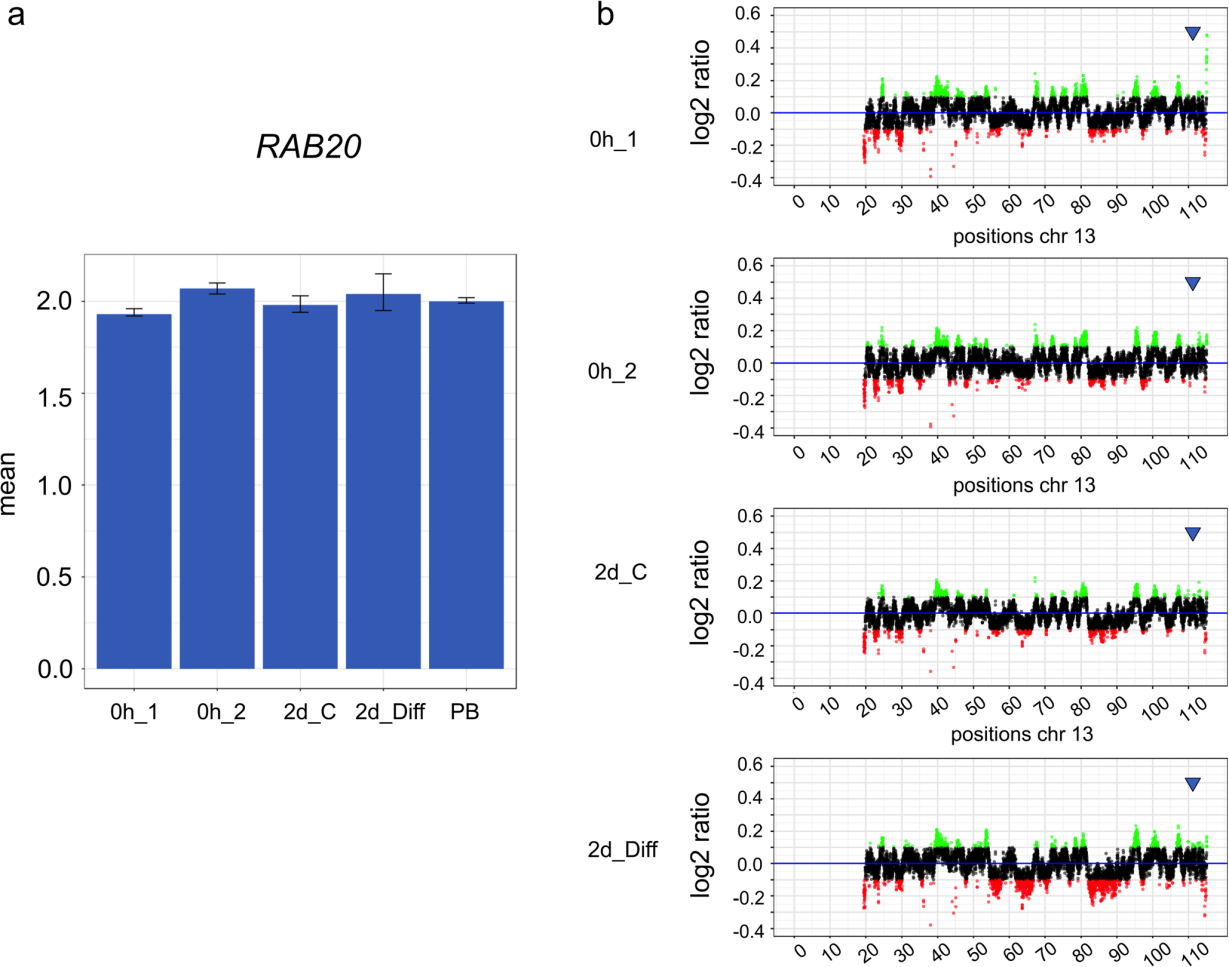
**a**, **b** Comparative analysis of copy numbers of the gene *RAB20* on chromosome 13 isolated from human neuronal stem cells (hNSCs) by qPCR (a) and WGS (b). The culture conditions of hNSCs, the source of the peripheral blood lymphocytes, and data display were the same as in Fig. 4. The chromosomal position of gene *RAB20* is indicated by a blue arrow

As for chromosome 12, WGS also indicated several copy number elevations along chromosome 16 (Fig. 5). In contrast, the low copy number elevation of the gene *RAB20* was only detected for specific cell culture conditions of hNSCs. As shown in Fig. 6, elevated copy number levels of *RAB20* were found in hNSCs without culturing by both WGS and qPCR, whereas elevated levels of *RAB20* that were found in hNSCs after 2 days of culturing without EGF/bFGF were detected only by qPCR but not by WGS.

**Fig. 7:**
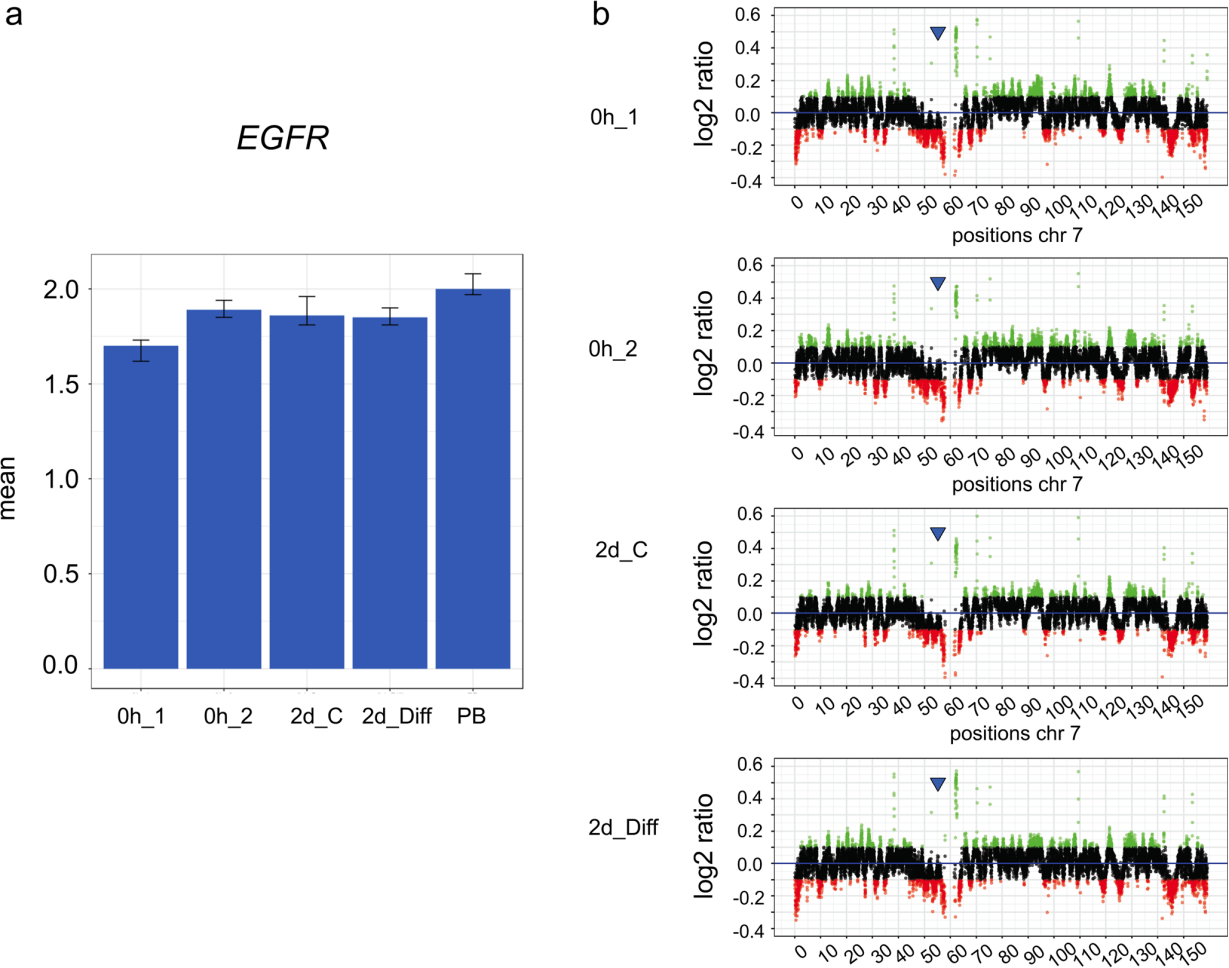
**a**, **b** Comparative analysis of copy numbers of the gene *EGFR* on chromosome 7 isolated from human neuronal stem cells (hNSCs) by qPCR and WGS. The culture conditions of hNSCs and data display were the same as in Fig. 4. The chromosomal position of gene *EGFR* is indicated by a blue arrow

**Fig. 8:**
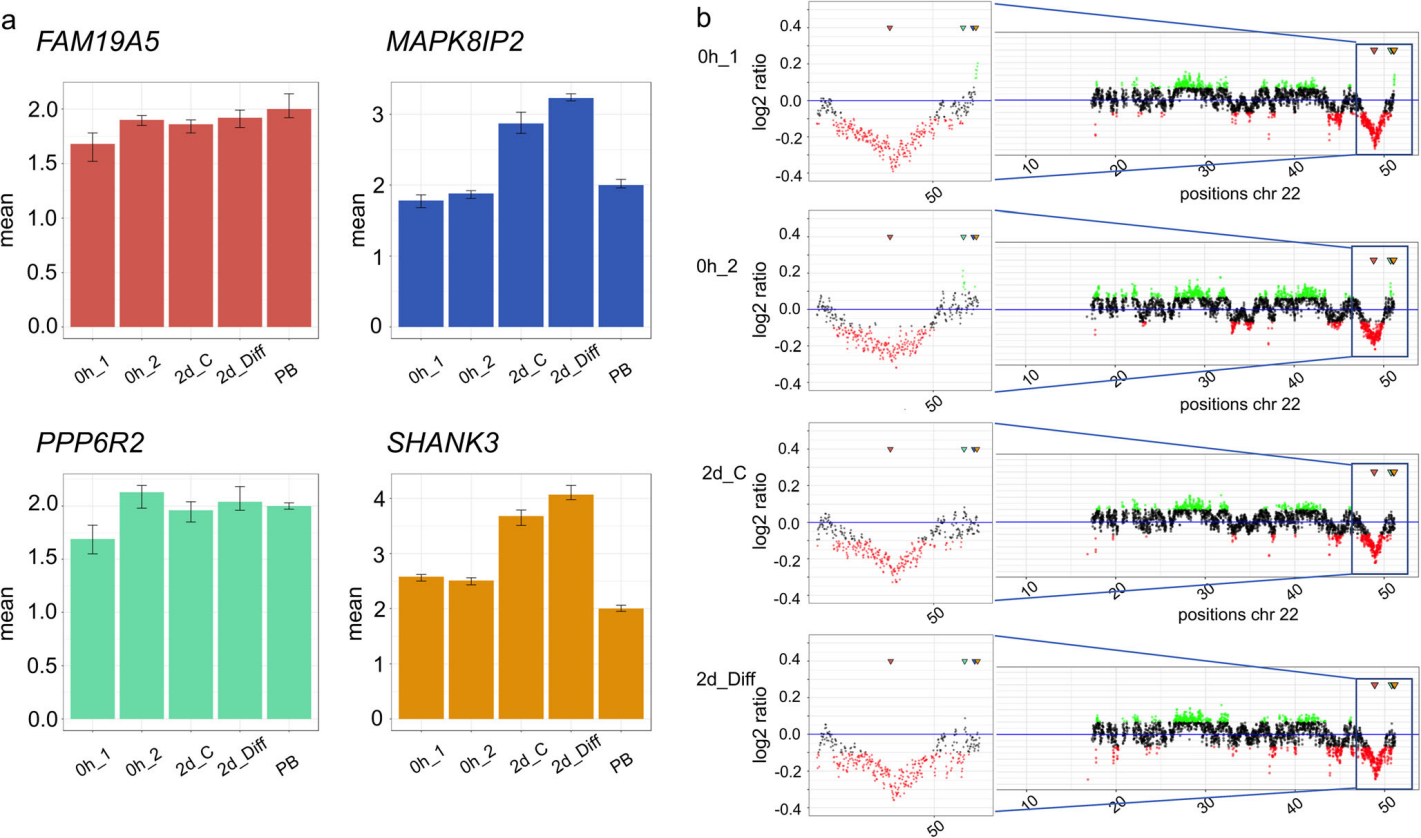
**a**, **b** Comparative analysis of copy numbers of the genes *FAM19A5*, *PPP6R2*, *MAPK8IP2*, and *SHANK3* on chromosome 22 isolated from human neuronal stem cells (hNSCs) by qPCR and WGS. The culture conditions of hNSCs and data display were as in Fig. 4. The region at 50 Mb is shown as an enlarged section. The chromosomal positions of the genes *FAM19A5*, *PPP6R2*, *MAPK8IP2*, and *SHANK3* are indicated by red, green, blue, and orange arrows, respectively

As shown in Fig. 7, reduced gene copy numbers can also be detected by WGS. qPCR showed a reduced copy number level of *EGFR* in hNSCs for all cell culture conditions, with the most prominent decrease for hNSCs, which were analyzed prior to culturing. Figure 8 shows examples with both elevated and reduced levels of copy numbers of genes that are located in close vicinity to each other on chromosome 22. qPCR revealed amplifications for *MAPK8IP2* and *SHANK3* in both hNSCs after 2 days of culturing with and without EGF/bFGF. Neither amplifications were detected using WGS. Amplification of *SHANK3*, however, was detected by both qPCR and WGS in hNSC without culturing. A strongly reduced copy number of *FAM19A5*, i.e., an under-replication, was found by WGS and qPCR for all cell culture conditions analyzed. An elevated copy number of *PPP6R2* was found by WGS and qPCR in hNSCs that have not been cultured.

A summary of the WGS analysis and direct comparison to the qPCR data is given in Table 3. The copy numbers are indicated by log_2_ ratio values 10 kb encompassing regions of the genes that have been analyzed by qPCR. Regions with copy number gains (> 0.05) are indicated by light green, regions with amplifications (≥ 0.1) by dark green, regions with reduced copy number (< − 0.05) by orange, and regions with under-replication by red (≤ − 0.1). Altogether, we tested amplification in nine genes and four sample, totaling 36 tests. As Table 3 highlights, the results between both technologies matched in 25 of the 36 cases (69.4%). If we include also these cases where the direction was concordant and the WGS results were thus not contradicting the qPCR results or vice versa, the concordance jumped to 30 of 36 tested cases (83.3%). In the absolute majority of cases that do not match, WGS did not report amplification events or losses where they were present according to the qPCR data, meaning that WGS is highly specific but has a slightly decreased sensitivity.

## Discussion

We tested several in silico strategies for an optimized detection of dynamic copy number increases in hMSC and hNSC by WGS. We used human whole genome sequencing 50-fold BGI SEQ 500 service. This includes rolling circle replication and a novel DNA nanoball (DNB™) technology that leads to less CG bias compared to PCR assays and assures high signal to noise ratio (SNR) imaging for accurate base calling. BGISEQ is a reliable sequencing platform as recently reported by Xu and colleagues who compared BGISEQ to HiSEQ4000 platform and BGISEQ demonstrated similarly high reproducibility as HiSeq for variation detection [20]. In addition, Mak and colleagues reported a comparative performance of the BGISEQ-500 versus Illumina HiSeq2500 for WGS of ancient DNA [21].

To this end, we used aCGH, qPCR, and FISH as reference methods.

### The need of complementary methods to identify dynamic copy number increases

The detection of dynamic copy number changes by high-throughput approaches is a major challenge that requires careful choice and adjustment of the appropriate methods. Our results show that both genome-wide approaches, aCGH and WGS, identify different copy number profiles. We demonstrated that several copy number changes on chromosome 22 of hMSCs were found by either aCGH or by WGS, but not by both methods. Hence, both methods require that each locus, for which a copy number change is identified, has to be confirmed by independent methods. Ideally, this has been done by methods that allow the analysis of single cells like the in situ hybridization. However, this method is rather time consuming and requires the selection of a suitable hybridization probe and the optimization of the hybridization conditions, rendering it less suitable for high-throughput analysis. qPCR is a second method that can also be tailored to the analysis of copy number changes of single loci. In contrast to in situ hybridization, qPCR allows the simultaneous analysis of a larger number of cells. This advantage impinges on the sensitivity of the detection of copy number changes that can be masked by a larger number of cells that do not carry an increased copy number of the analyzed locus.

### The threshold settings to efficiently identify copy number increases

Overall, the detection of dynamic copy number changes requires not only the complementary use of several techniques but also adapted in silico methods. Standard software for amplification and CNV detection with highly stringent thresholds failed to detect amplifications in the WGS data from the stem cells hMSC and hNSC. Using a less stringent threshold, we detected gene amplifications in all samples. While the genome-wide methods aCGH and WGS allow the identification of yet unknown copy number changes, both methods may readily miss an elevated copy number such as the copy number increase at 24.3 Mb of chromosome 22 in hMSCs (not detected by WGS) or copy number increase at 32 Mb of chromosome 22 (not detected by aCGH). Recently, gene amplifications could not be demonstrated by whole genome sequencing including DNASeq and BICSeq strategies in EVT (extravillous trophoblast) cells during placenta development, despite small EVT subpopulations are likely to contain elevated gene copy numbers [11]. Although qPCR focuses on changes of single loci for copy number changes, the sensitivity of the qPCR approach depends, similar to the sensitivity of aCGH and WGS, on the ratio between cells that carry the elevated copy number of a given locus and cells that carry the normal diploid copy number of this locus. Hence, the setting of a suitable threshold is a major challenge for qPCR, aCGH, and WGS. While too low thresholds will yield too many false-positive hints for copy number changes, too high thresholds will miss real copy number changes. Since the ratio between cells with an increased copy number of a given locus and the cells without such copy number increase is a prior unknown, the threshold setting is largely to be determined by trial and error. Repeated analyses of data sets with known copy number increases confirmed by in situ hybridization or other methods allow the analysis of single loci in single cells, which can in the long run certainly help to optimize threshold settings.

### The need to gain overall knowledge of increased copy numbers in stem cells

The difficulties of obtaining a better picture of dynamic copy number changes can hardly be underestimated. As aforementioned, the increased gene copy numbers in mammalian stem cells seem to be restricted to specific tissue areas and to specific time windows. The most challenging will be scenarios with a rather low copy number increase in a relatively low number of cells within a tissue that is otherwise normal, with regard to the copy number of the loci under investigation. The present analysis is based on substantial preparatory work that identified the copy number increases in human stem cells, including human mesenchymal stem cells and neural stem cells, under specific conditions that were found to prompt the development of copy number increases. Comparable studies need to be done on an extended number of cell types, including different types of stem cells. Here, the use of genome-wide high-throughput approaches, most notably WGS, can tremendously help to identify dynamic copy number changes. A more comprehensive knowledge of the landscape of dynamic copy number increases will yield new reference values that in turn can be used to further optimize approaches to identify dynamic copy number changes.

### The inherent limitations of WGS for the detection of copy number increases

It is well established that gene amplifications in tumor cells can be reliably detected by various methods, including WGS. There are several reasons that amplifications in stem cells are far more difficult to trace by WGS. As aforementioned, small numbers of cells with amplifications within narrow time windows complicate the detection via WGS. In addition, myoblast differentiation is accompanied by multiple double-strand breaks, as shown in mouse myoblasts that develop into contractile myotubes [22, 23]. We recently showed that myotube differentiation is not only accompanied by double-strand breaks, but also by gene amplifications [6]. The double-strand breaks lead to broken DNA fragments that can easily be lost during the size selection of WGS library preparation. As a result, the amplified fragments are also lost and are not detected in stem cells by WGS. This effect was most obvious for *SHANK3* amplification in differentiating hNSC cells and could not be detected with WGS, but *SHANK3* amplification could be detected in undifferentiated hNSCs. This assumption may in addition explain why WGS revealed higher log_2_ ratio values for *CDK4* in undifferentiated hNSC samples, as compared to differentiating hNSCs. Notably, hNSCs which differentiate by depletion of growth factors carry higher copy numbers of *CDK4* than undifferentiated hNSCs, as shown by qPCR analysis. This is supported by the observation of Wang et al. that growth factor depletion leads to differentiation in neural stem cells [24]. Possibly, these inherent limitations of WGS on the BGISEQ-500RS can in the future be overcome by using WGS approaches that allow longer reads that are less vulnerable to omission during library preparation.

We show that WGS allows for the identification of dynamic copy number changes in human stem cells. We would like to stress that WGS and aCGH are complementary methods and identified changes require confirmation by other methods like qPCR, or FISH. Our results suggest that optimized experimental and in silico strategies for amplification detection will further help to identify copy number increases, even under circumstances in which the copy number increase is limited to a numbered group of cells and to a narrow time frame.

## Electronic supplementary material

ESM 1 (PDF 69536 kb)
