# Supplementary material for: Prospect and challenge of detecting dynamic gene copy number increases in stem cells by whole genome sequencing
Source: J Mol Med (Berl). 2019 May 27;97(8):1099–111. doi: 10.1007/s00109-019-01792-y (PMC6647207; doi:10.1007/s00109-019-01792-y)
Supplement: Supplementary file 1 — (PDF 69536 kb) [file 109_2019_1792_MOESM1_ESM.pdf]

0h_1 window: 25kb threshold: 0.1

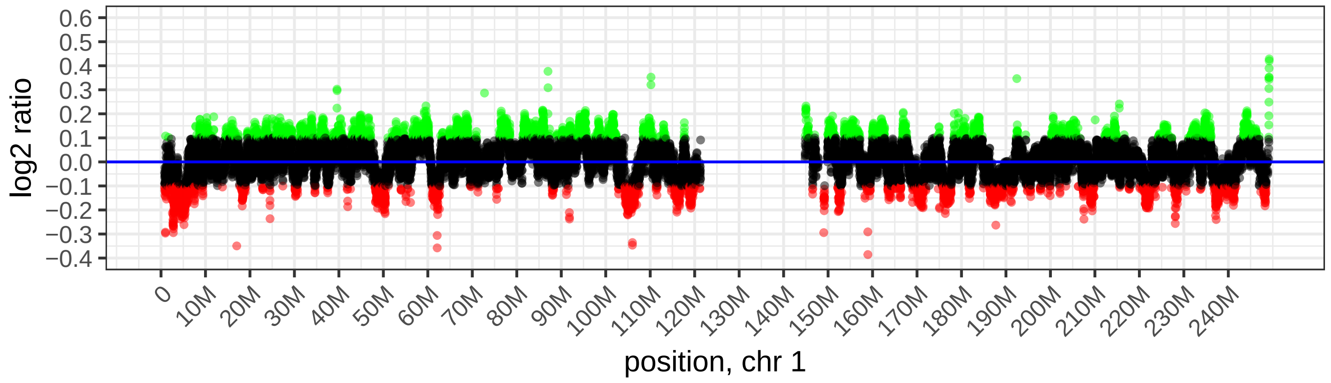

0h_2 window: 25kb threshold: 0.1

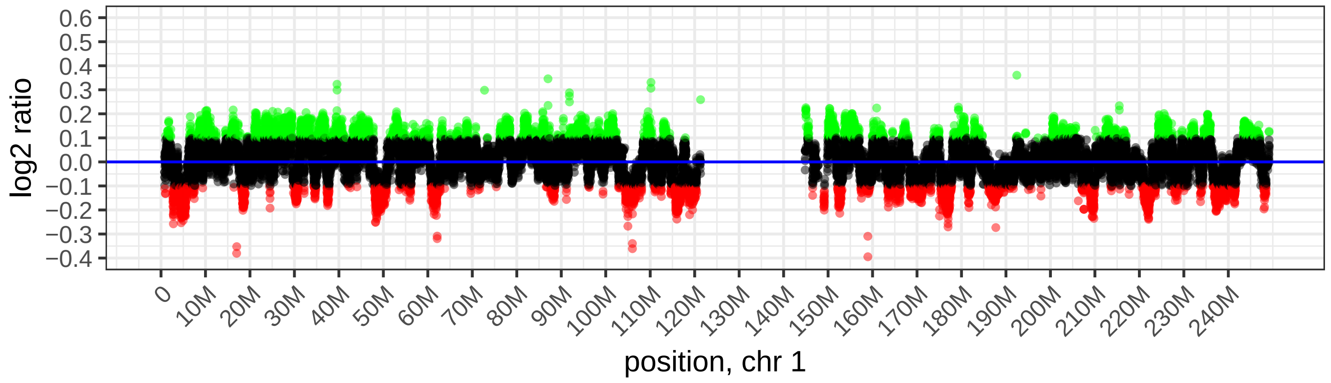

2d_C window: 25kb threshold: 0.1

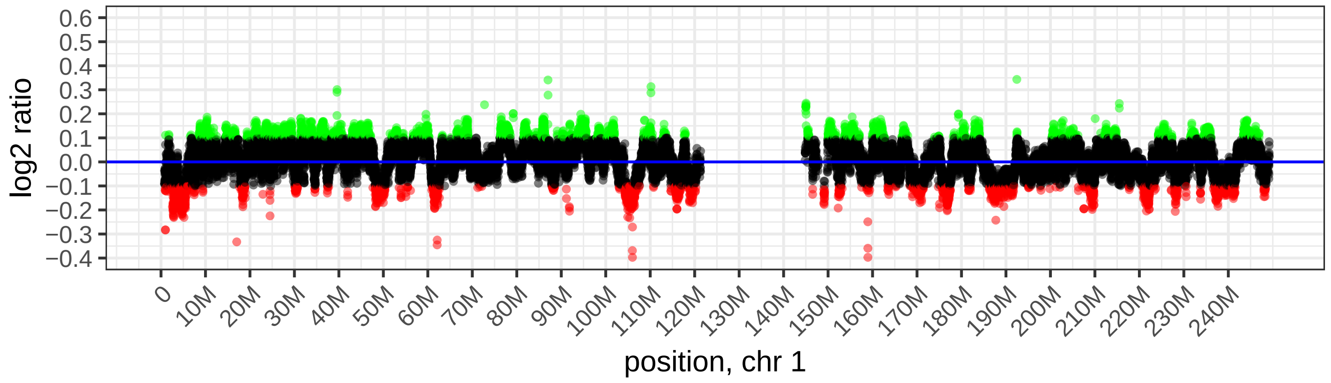

2d_Diff window: 25kb threshold: 0.1

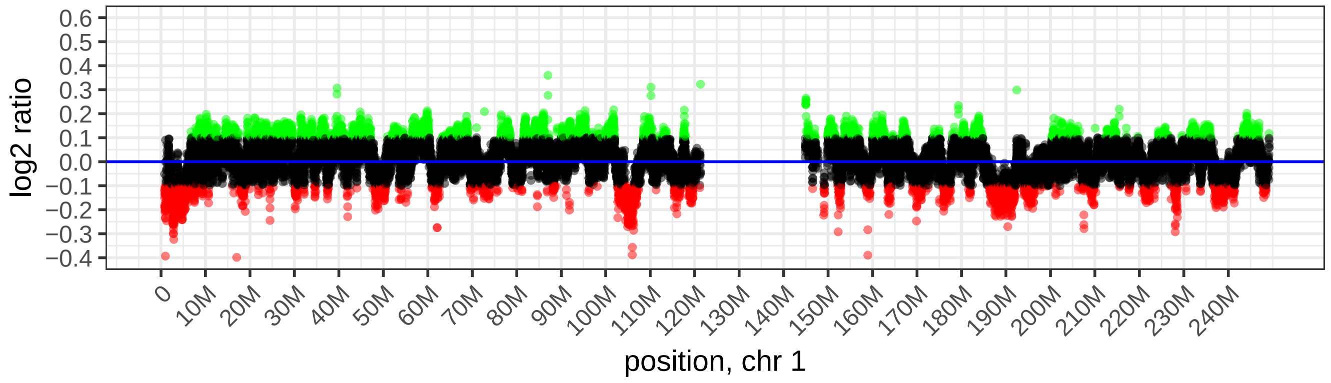

hMSC window: 25kb threshold: 0.1

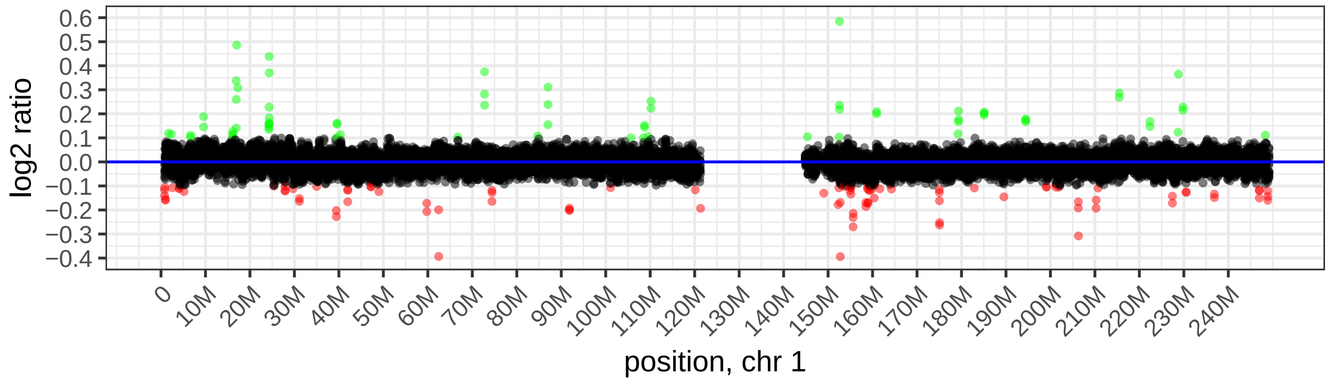

hMSC_aCGH window: 25kb threshold: 0.1

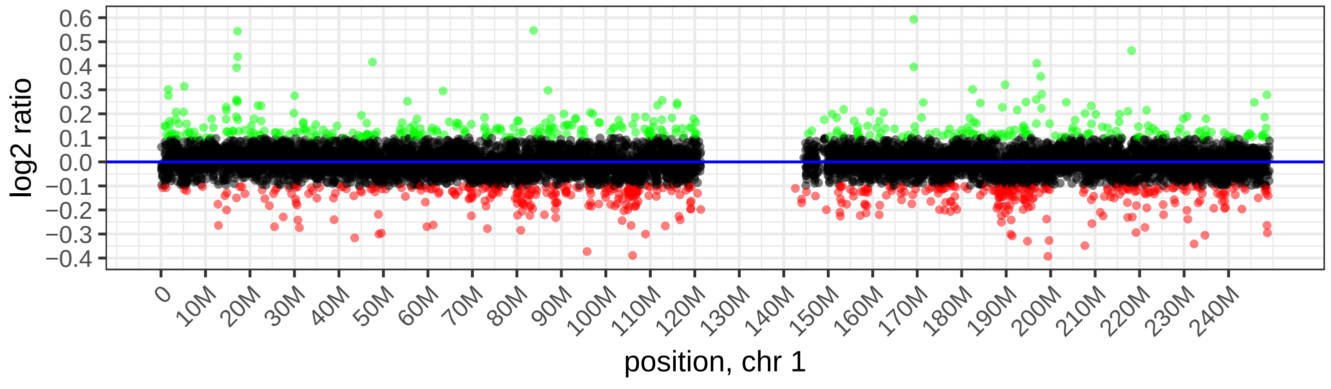

0h_1 window: 25kb threshold: 0.1

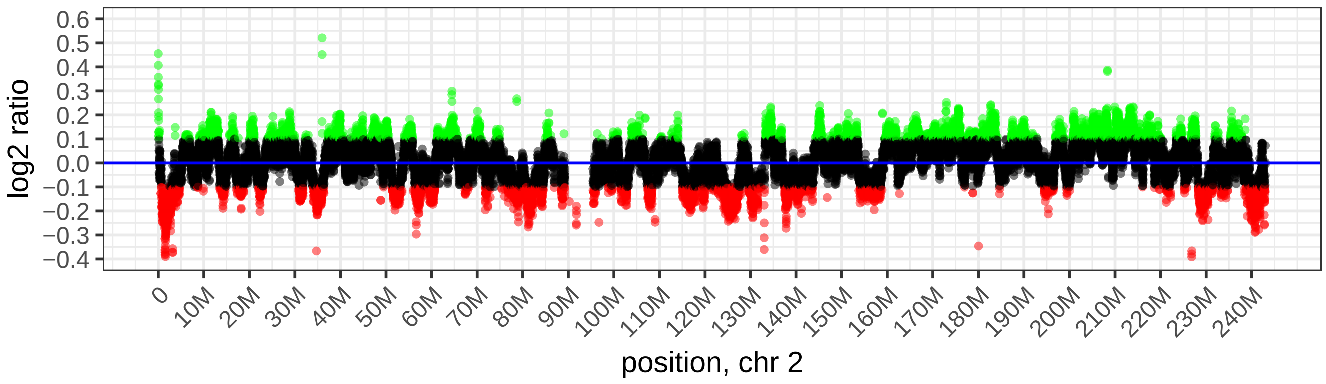

0h_2 window: 25kb threshold: 0.1

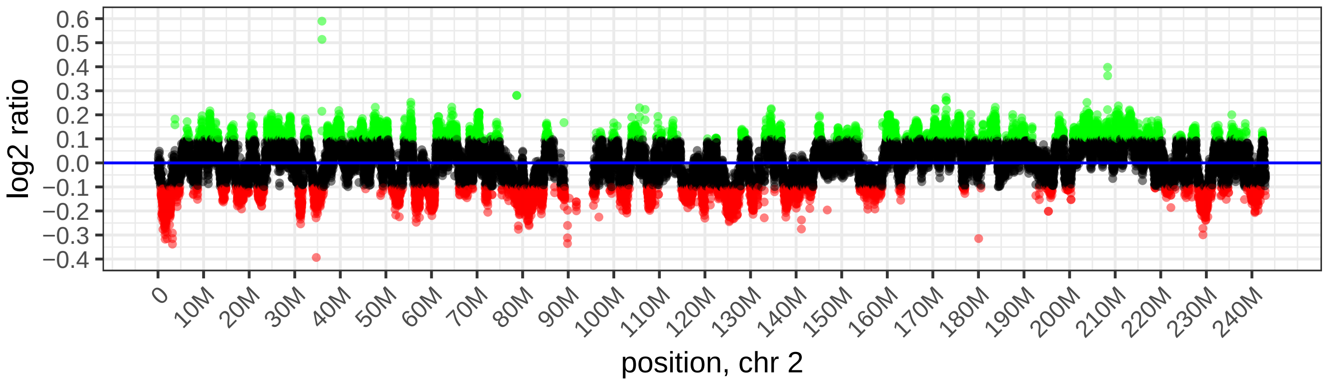

2d_C window: 25kb threshold: 0.1

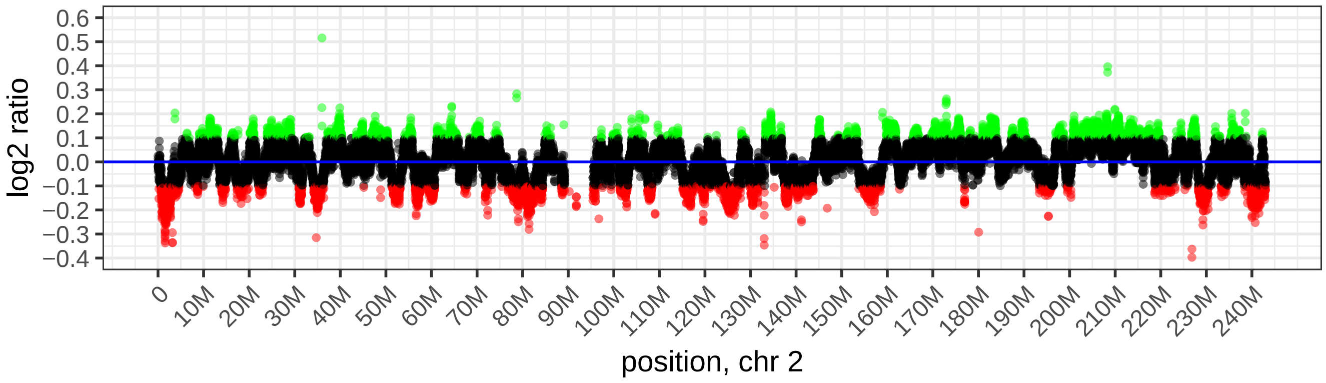

2d_Diff window: 25kb threshold: 0.1

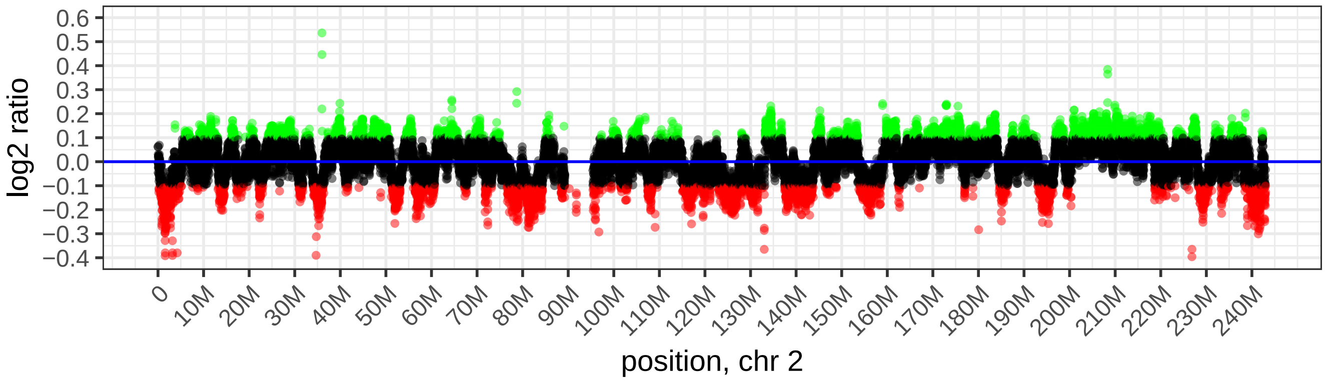

hMSC window: 25kb threshold: 0.1

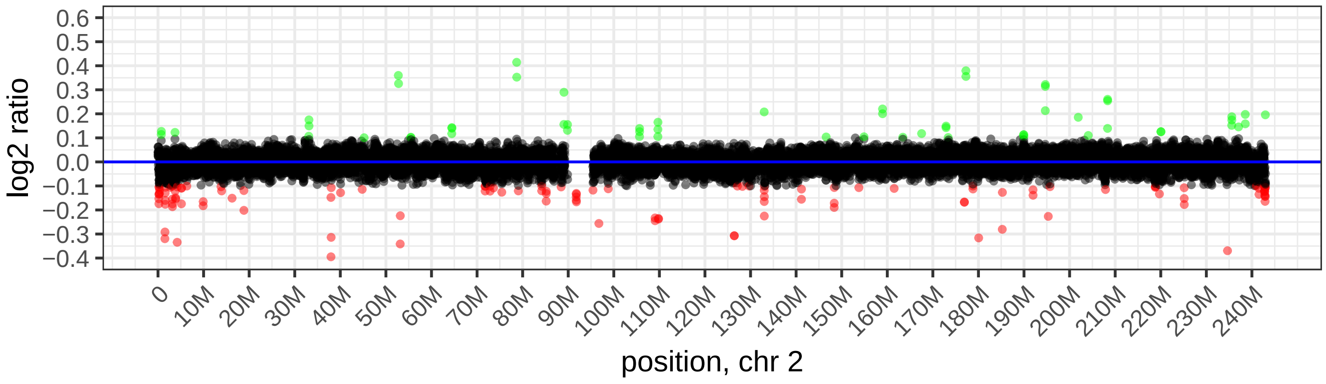

hMSC_aCGH window: 25kb threshold: 0.1

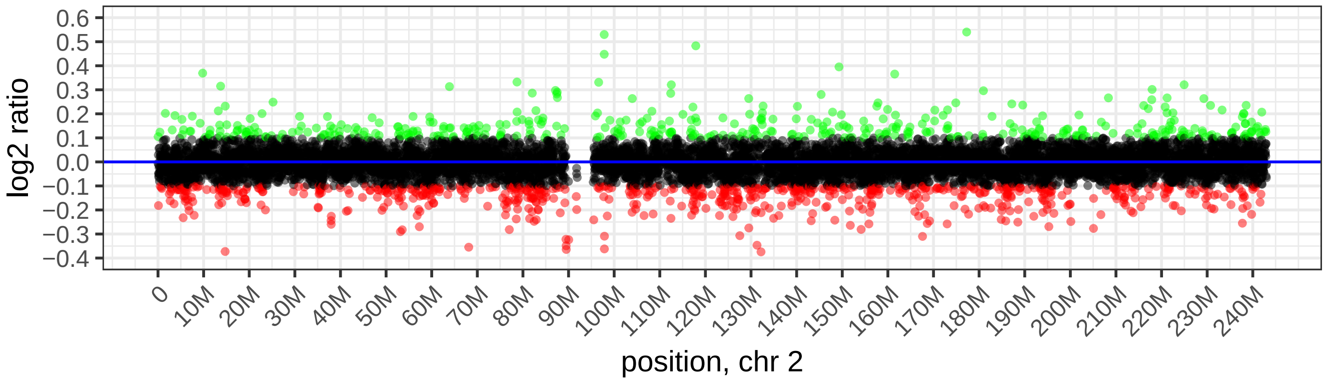

0h_1 window: 25kb threshold: 0.1

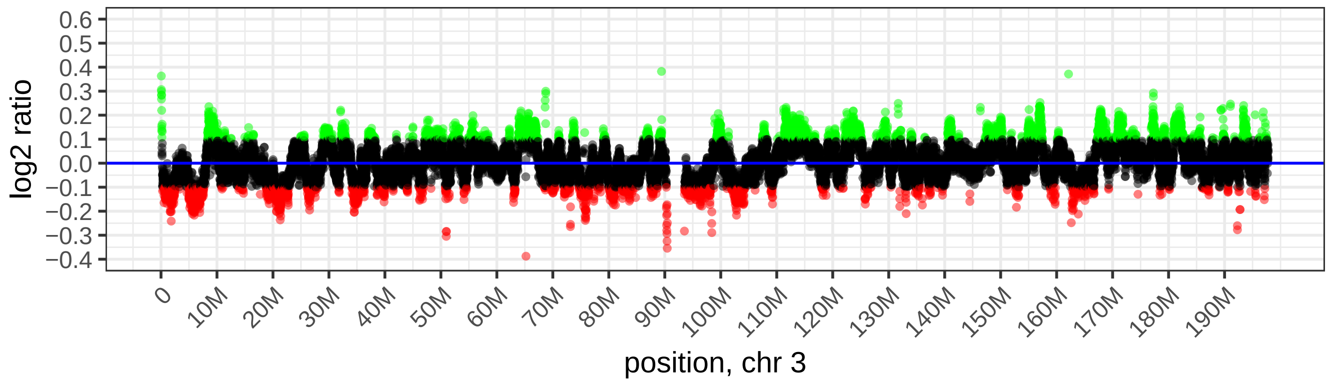

0h_2 window: 25kb threshold: 0.1

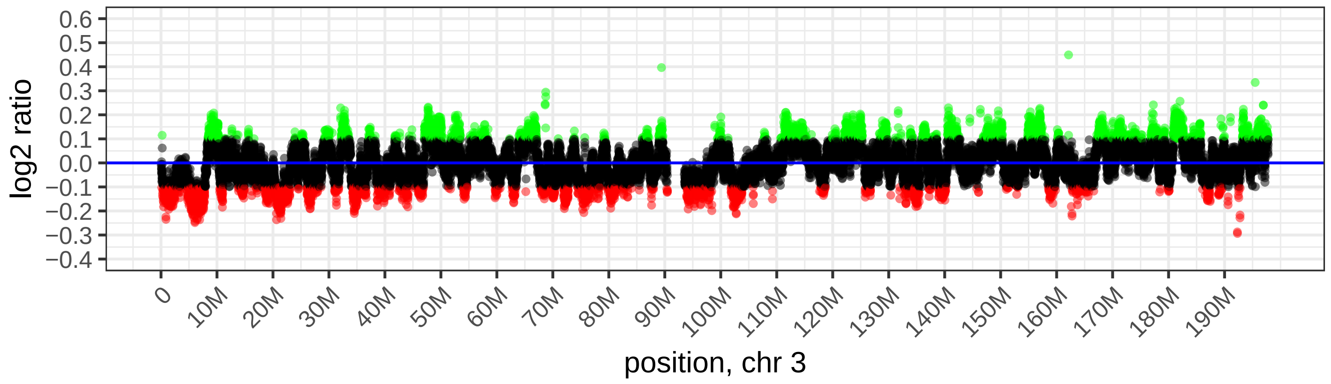

2d_C window: 25kb threshold: 0.1

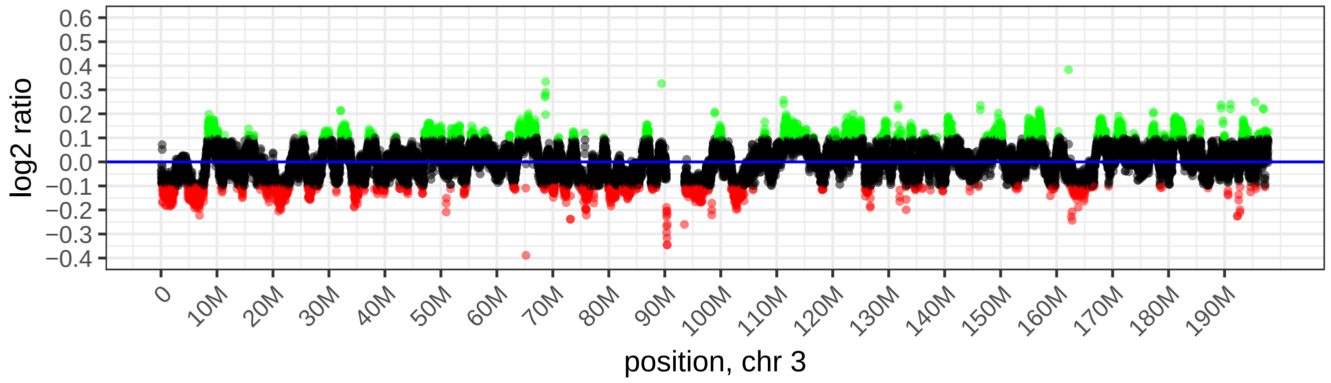

2d_Diff window: 25kb threshold: 0.1

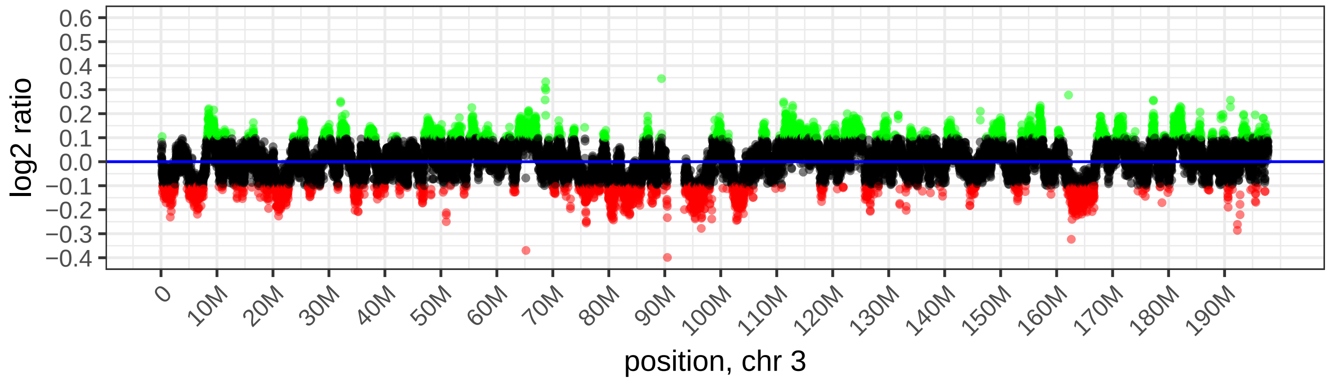

hMSC window: 25kb threshold: 0.1

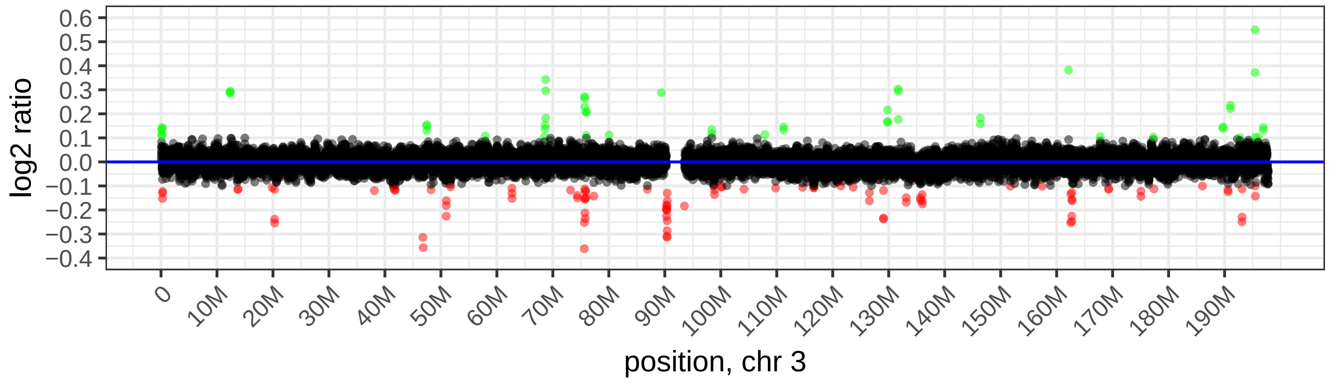

hMSC_aCGH window: 25kb threshold: 0.1

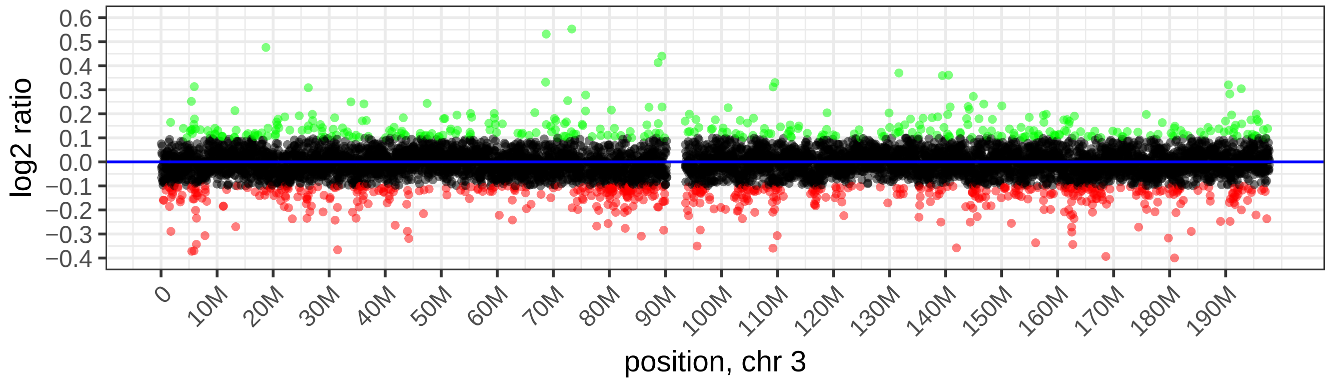

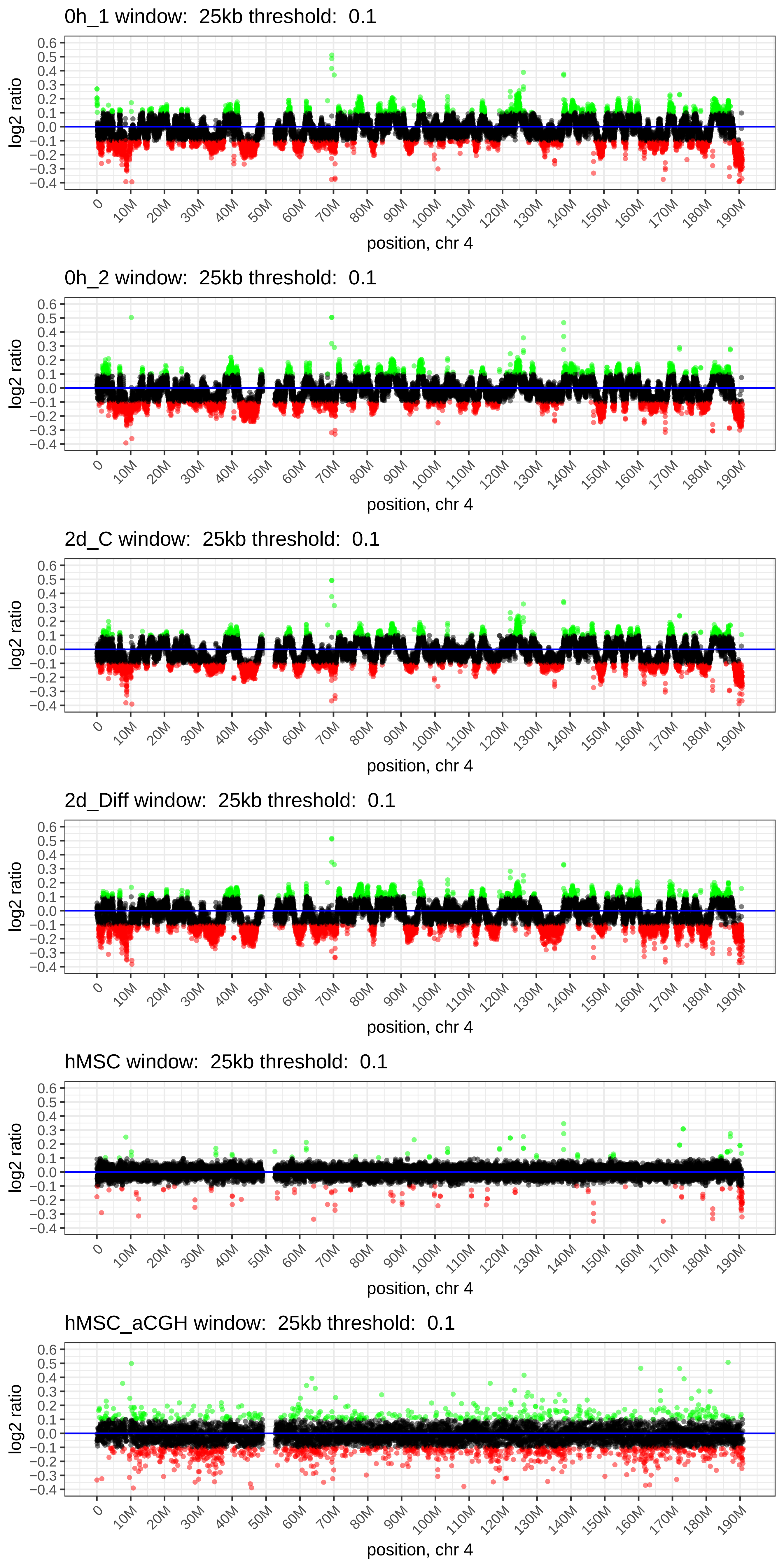
0h_1 window: 25kb threshold: 0.1
0h_2 window: 25kb threshold: 0.1
2d_C window: 25kb threshold: 0.1
2d_Diff window: 25kb threshold: 0.1
hMSC window: 25kb threshold: 0.1
hMSC_aCGH window: 25kb threshold: 0.1
log2 ratio
position, chr 4
0.6
0.5
0.4
0.3
0.2
0.1
0.0
-0.1
-0.2
-0.3
-0.4
0
10M
20M
30M
40M
50M
60M
70M
80M
90M
100M
110M
120M
130M
140M
150M
160M
170M
180M
190M

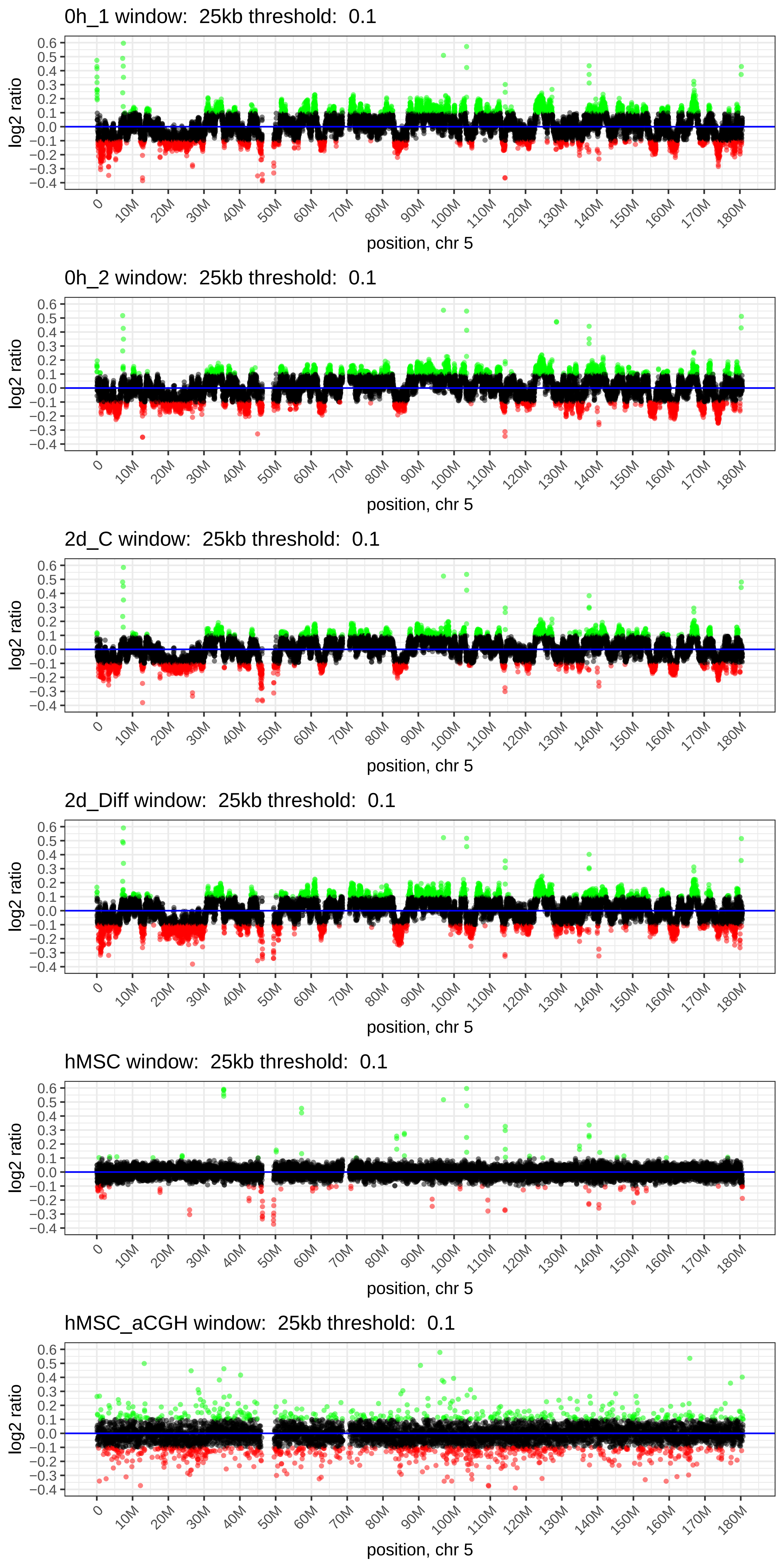
0h_1 window: 25kb threshold: 0.1
log2 ratio
0.6
0.5
0.4
0.3
0.2
0.1
0.0
-0.1
-0.2
-0.3
-0.4
0
10M
20M
30M
40M
50M
60M
70M
80M
90M
100M
110M
120M
130M
140M
150M
160M
170M
180M
position, chr 5
0h_2 window: 25kb threshold: 0.1
log2 ratio
position, chr 5
2d_C window: 25kb threshold: 0.1
log2 ratio
position, chr 5
2d_Diff window: 25kb threshold: 0.1
log2 ratio
position, chr 5
hMSC window: 25kb threshold: 0.1
log2 ratio
position, chr 5
hMSC_aCGH window: 25kb threshold: 0.1
log2 ratio
position, chr 5

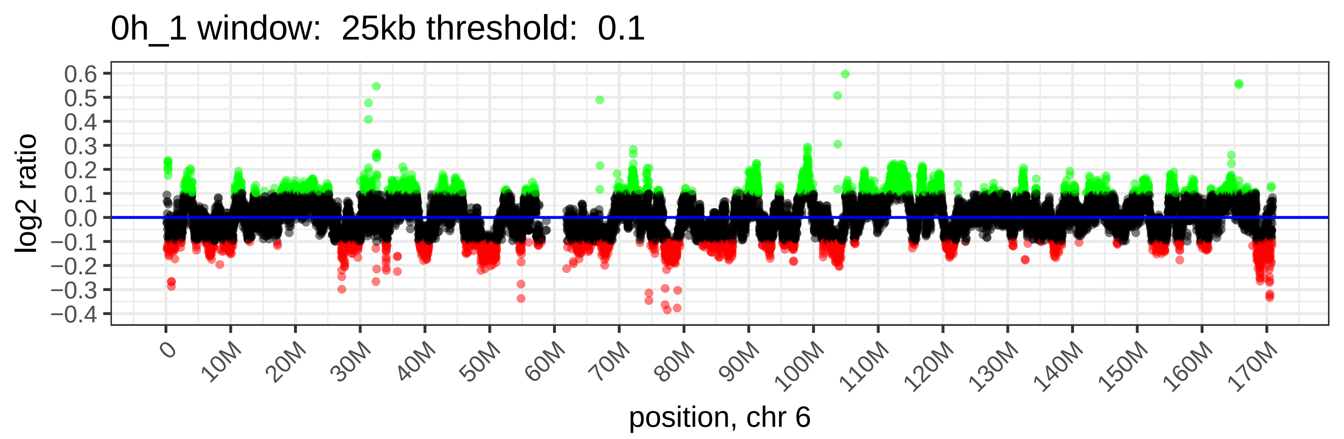
0h_1 window: 25kb threshold: 0.1
log2 ratio
position, chr 6

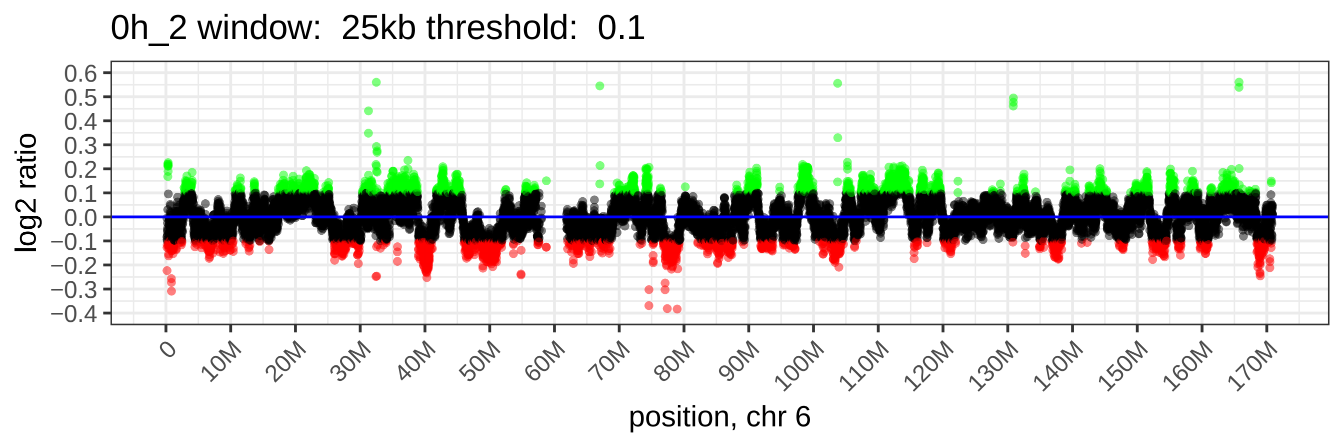
0h_2 window: 25kb threshold: 0.1
log2 ratio
position, chr 6

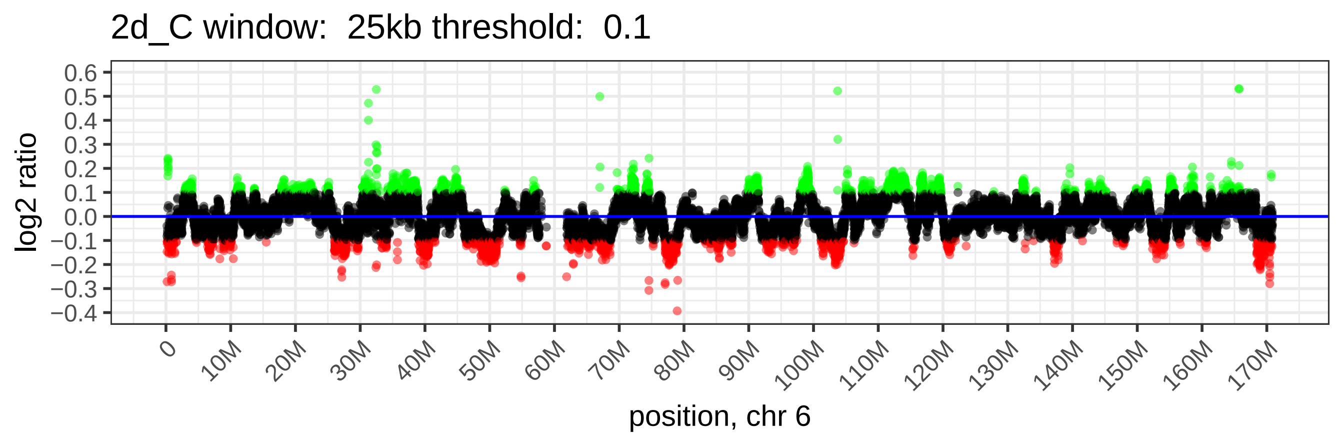
2d_C window: 25kb threshold: 0.1
log2 ratio
position, chr 6

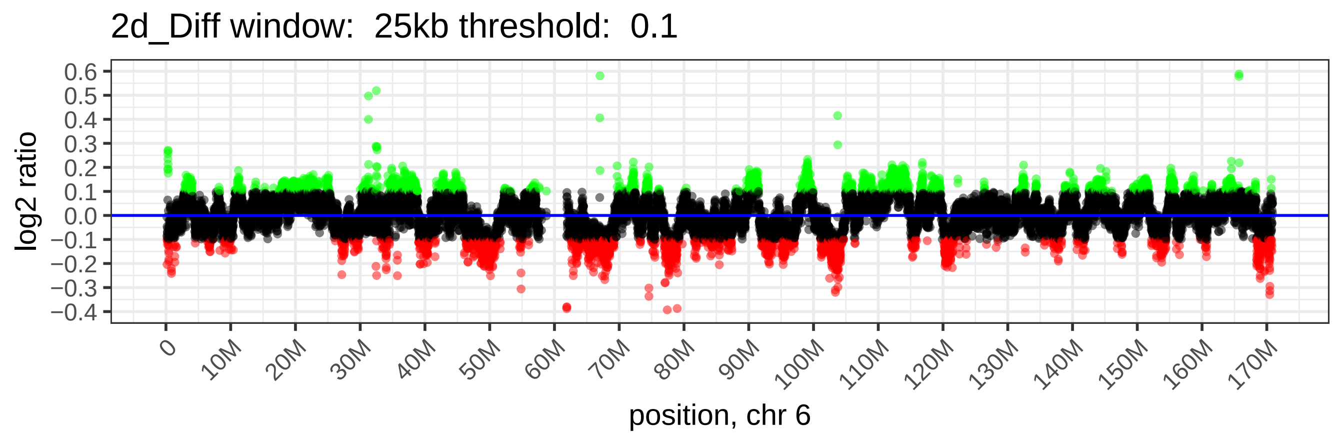
2d_Diff window: 25kb threshold: 0.1
log2 ratio
position, chr 6

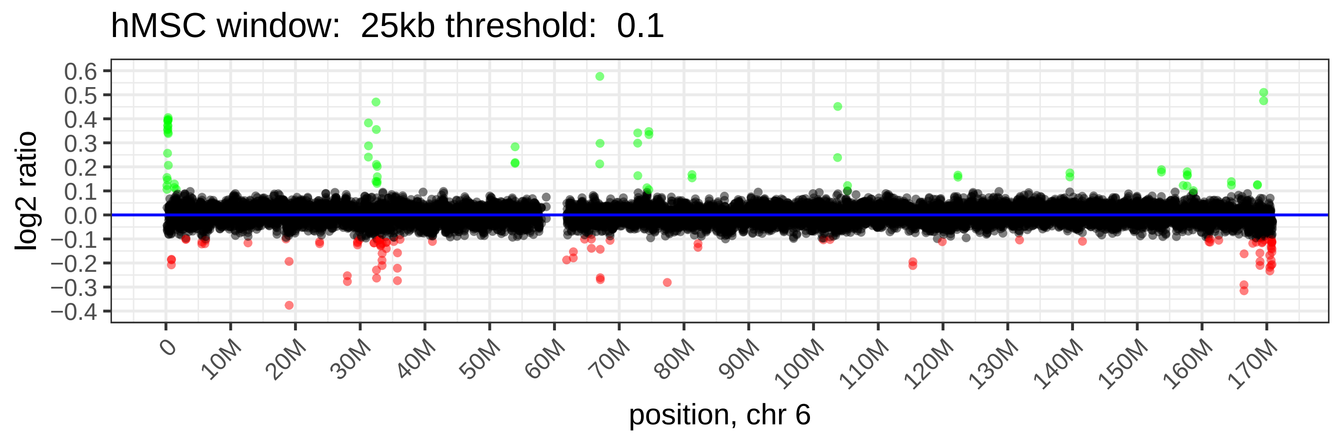
hMSC window: 25kb threshold: 0.1
log2 ratio
position, chr 6

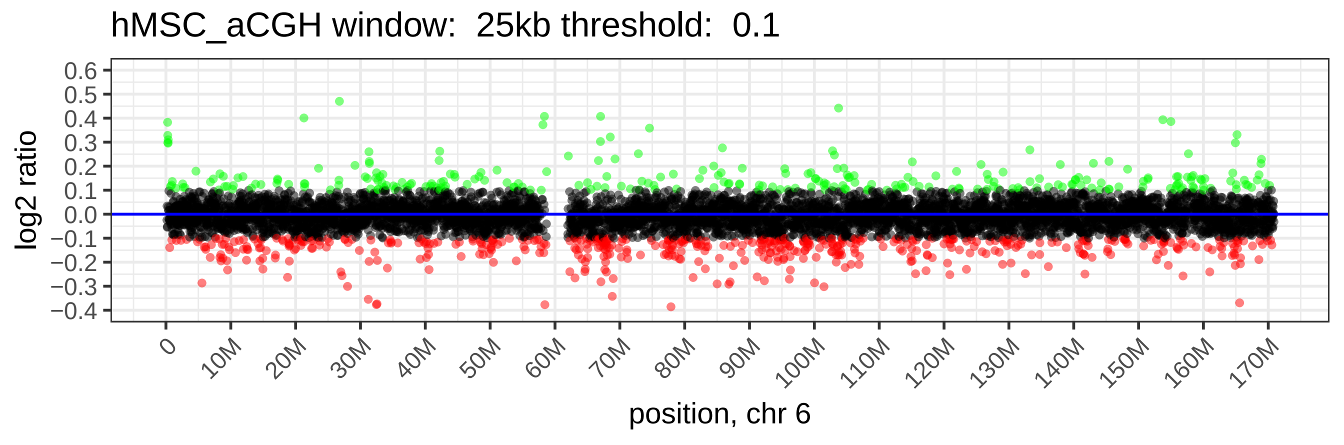
hMSC_aCGH window: 25kb threshold: 0.1
log2 ratio
position, chr 6

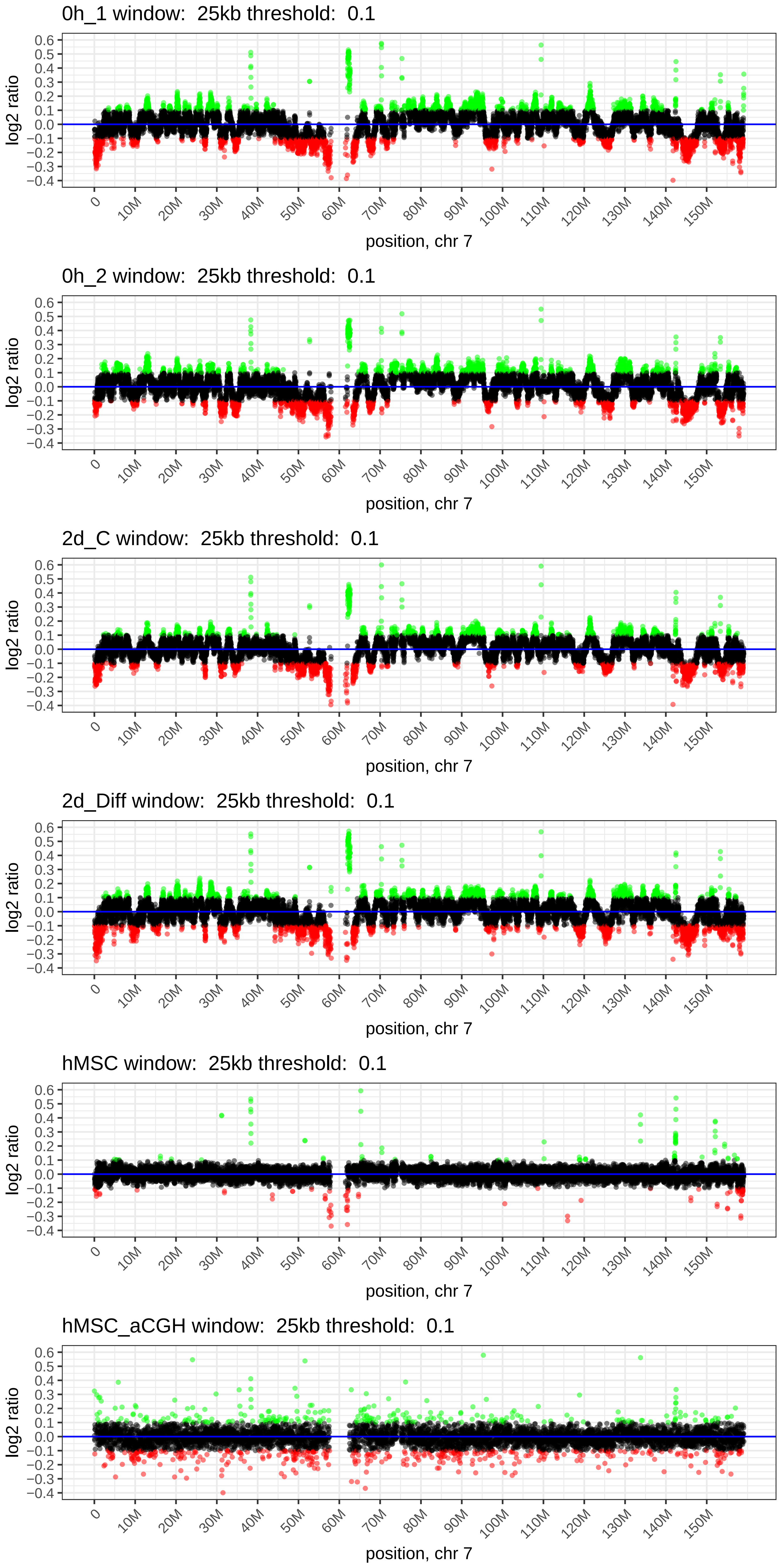
0h_1 window: 25kb threshold: 0.1
0h_2 window: 25kb threshold: 0.1
2d_C window: 25kb threshold: 0.1
2d_Diff window: 25kb threshold: 0.1
hMSC window: 25kb threshold: 0.1
hMSC_aCGH window: 25kb threshold: 0.1
log2 ratio
0.6
0.5
0.4
0.3
0.2
0.1
0.0
-0.1
-0.2
-0.3
-0.4
0
10M
20M
30M
40M
50M
60M
70M
80M
90M
100M
110M
120M
130M
140M
150M
position, chr 7

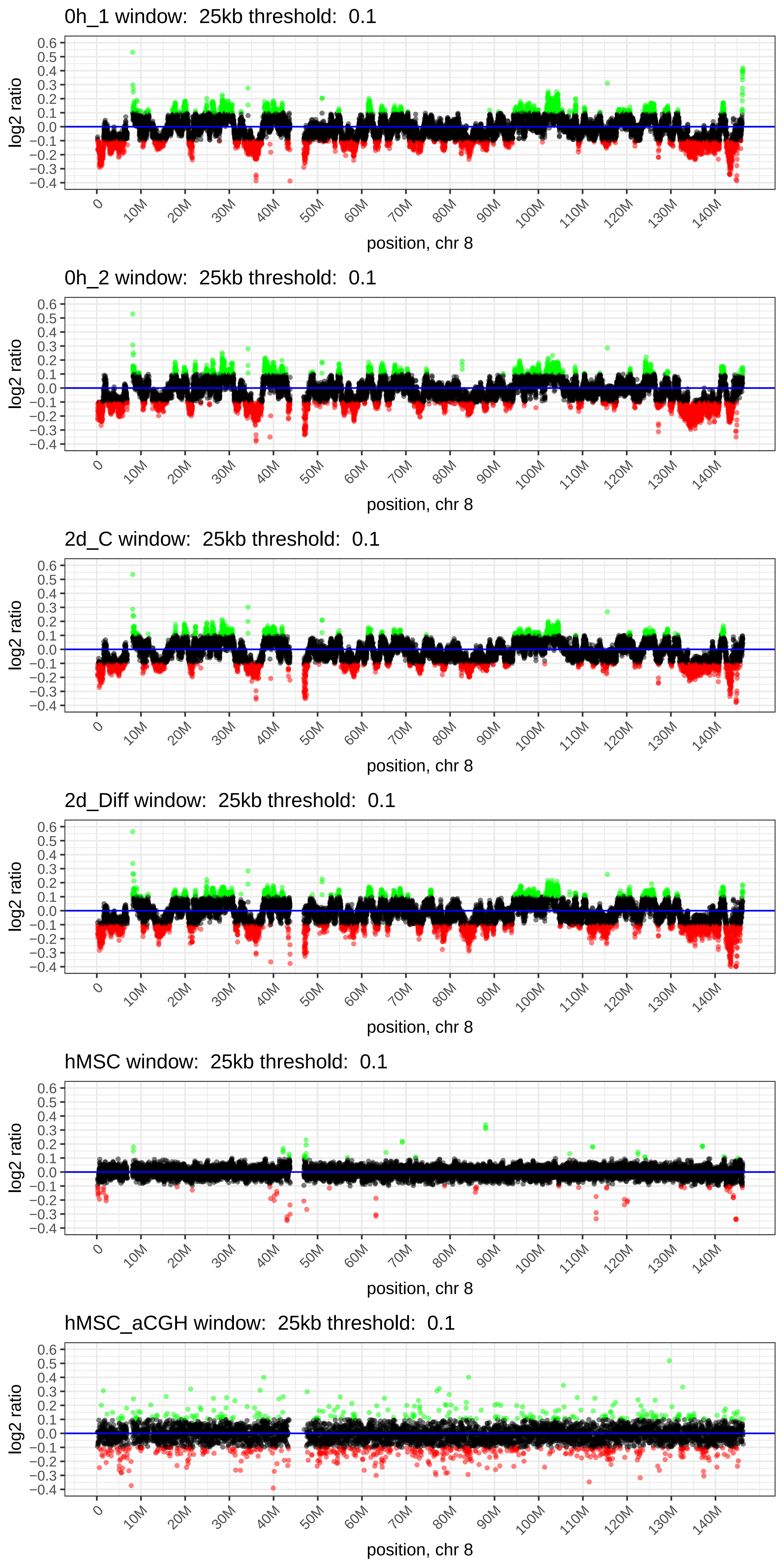
0h_1 window: 25kb threshold: 0.1
0h_2 window: 25kb threshold: 0.1
2d_C window: 25kb threshold: 0.1
2d_Diff window: 25kb threshold: 0.1
hMSC window: 25kb threshold: 0.1
hMSC_aCGH window: 25kb threshold: 0.1
log2 ratio
position, chr 8
0.6
0.5
0.4
0.3
0.2
0.1
0.0
-0.1
-0.2
-0.3
-0.4
0
10M
20M
30M
40M
50M
60M
70M
80M
90M
100M
110M
120M
130M
140M

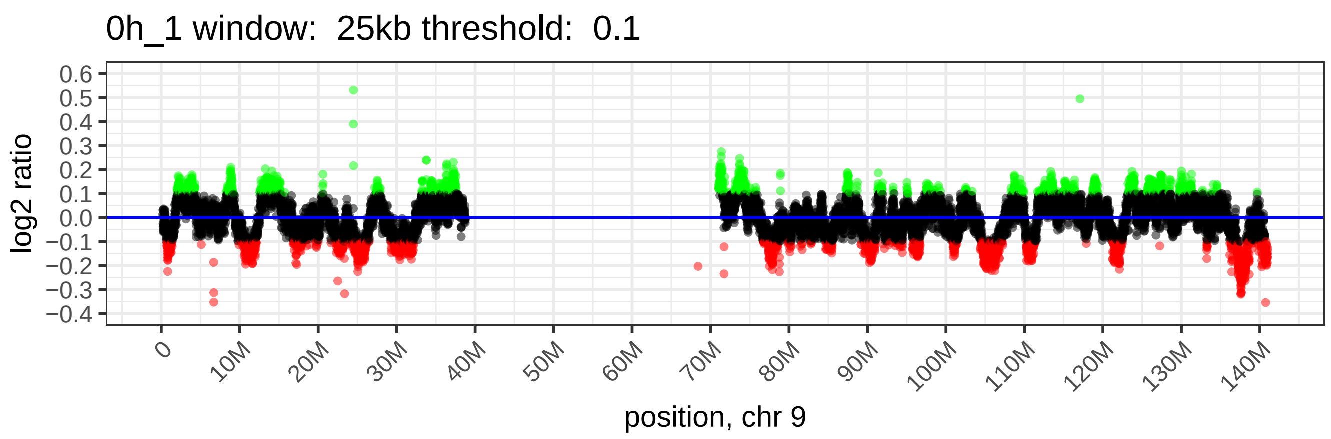
0h_1 window: 25kb threshold: 0.1
log2 ratio
0.6
0.5
0.4
0.3
0.2
0.1
0.0
-0.1
-0.2
-0.3
-0.4
0
10M
20M
30M
40M
50M
60M
70M
80M
90M
100M
110M
120M
130M
140M
position, chr 9

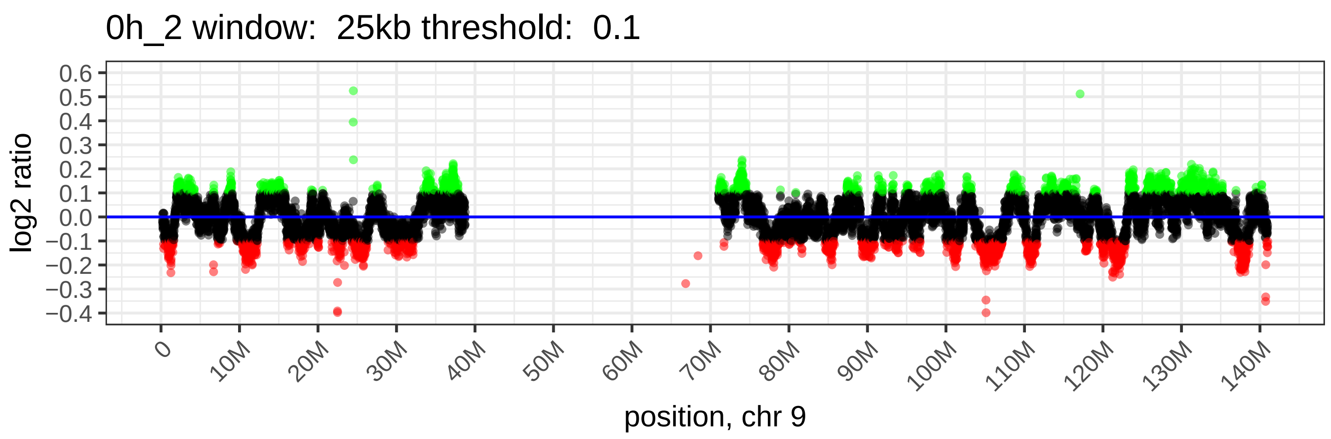
0h_2 window: 25kb threshold: 0.1
log2 ratio
0.6
0.5
0.4
0.3
0.2
0.1
0.0
-0.1
-0.2
-0.3
-0.4
0
10M
20M
30M
40M
50M
60M
70M
80M
90M
100M
110M
120M
130M
140M
position, chr 9

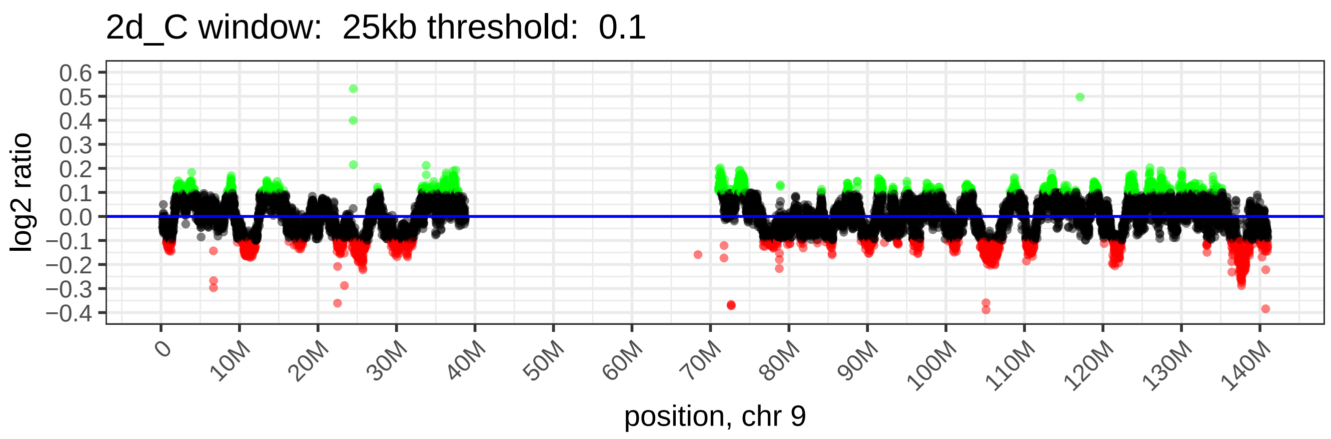
2d_C window: 25kb threshold: 0.1
log2 ratio
0.6
0.5
0.4
0.3
0.2
0.1
0.0
-0.1
-0.2
-0.3
-0.4
0
10M
20M
30M
40M
50M
60M
70M
80M
90M
100M
110M
120M
130M
140M
position, chr 9

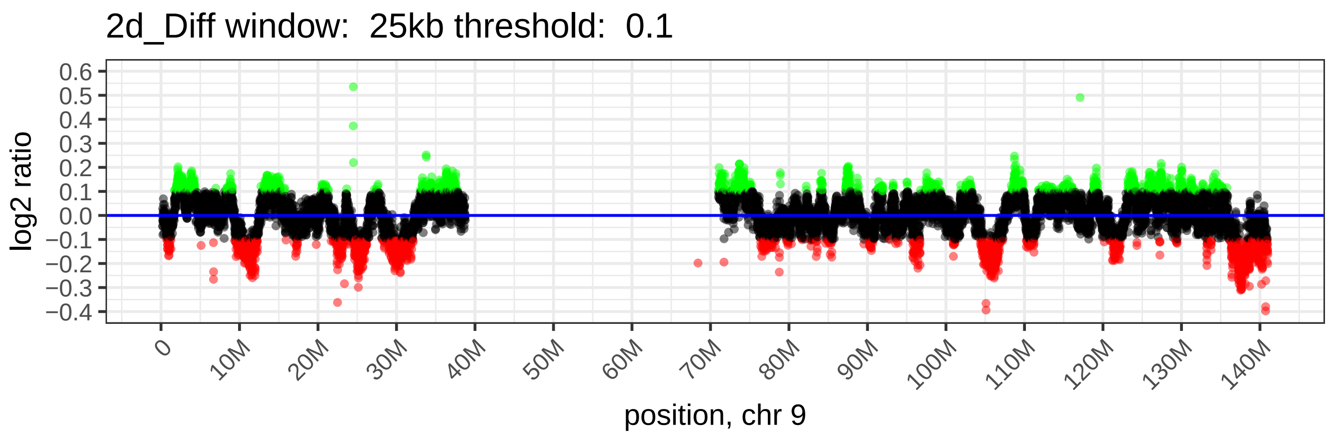
2d_Diff window: 25kb threshold: 0.1
log2 ratio
0.6
0.5
0.4
0.3
0.2
0.1
0.0
-0.1
-0.2
-0.3
-0.4
0
10M
20M
30M
40M
50M
60M
70M
80M
90M
100M
110M
120M
130M
140M
position, chr 9

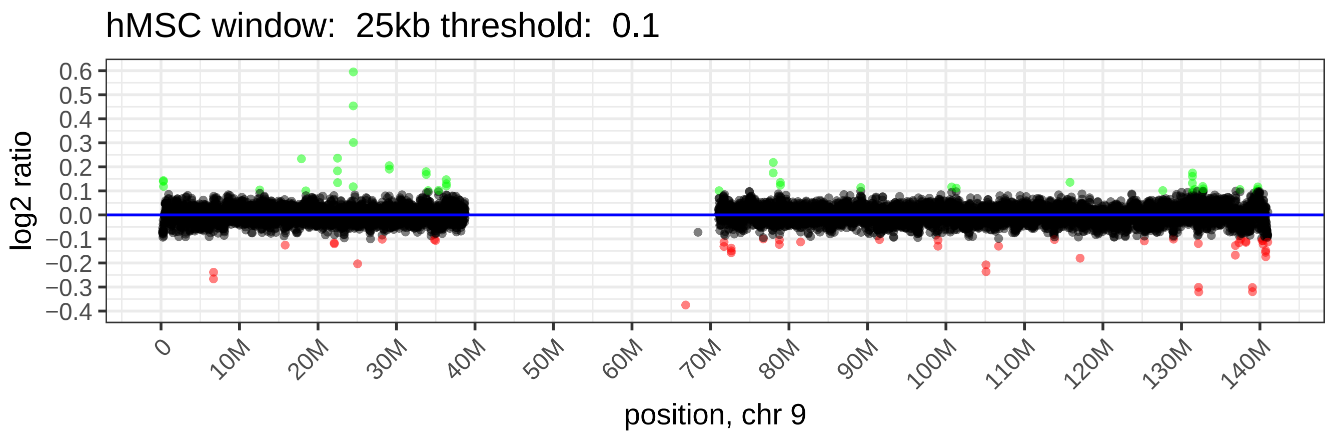
hMSC window: 25kb threshold: 0.1
log2 ratio
0.6
0.5
0.4
0.3
0.2
0.1
0.0
-0.1
-0.2
-0.3
-0.4
0
10M
20M
30M
40M
50M
60M
70M
80M
90M
100M
110M
120M
130M
140M
position, chr 9

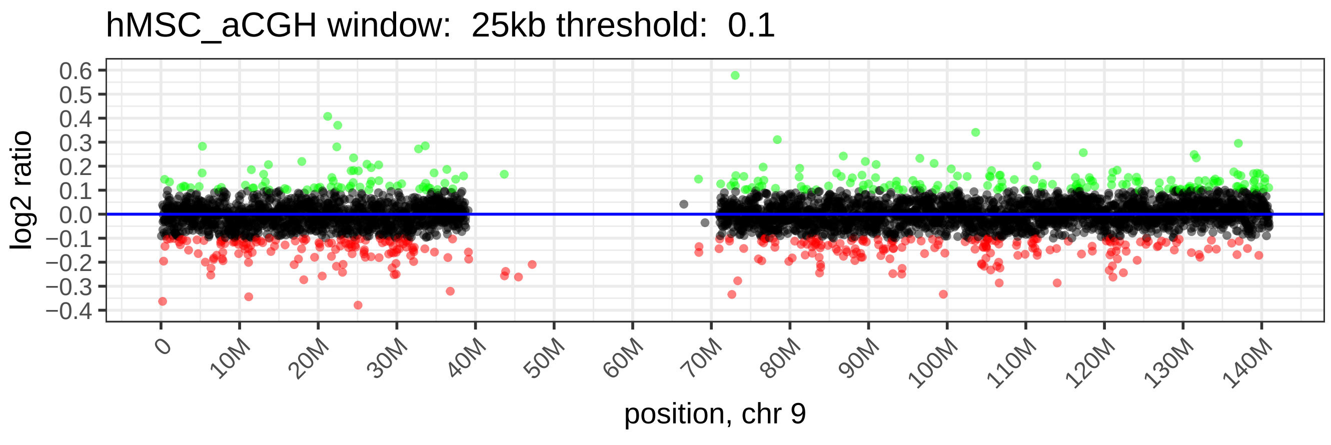
hMSC_aCGH window: 25kb threshold: 0.1
log2 ratio
0.6
0.5
0.4
0.3
0.2
0.1
0.0
-0.1
-0.2
-0.3
-0.4
0
10M
20M
30M
40M
50M
60M
70M
80M
90M
100M
110M
120M
130M
140M
position, chr 9

0h_1 window: 25kb threshold: 0.1

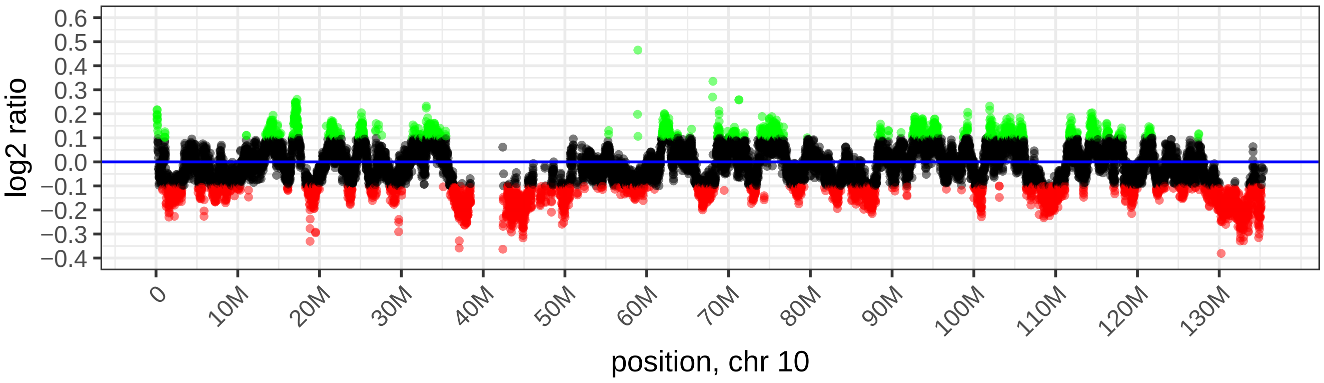

0h_2 window: 25kb threshold: 0.1

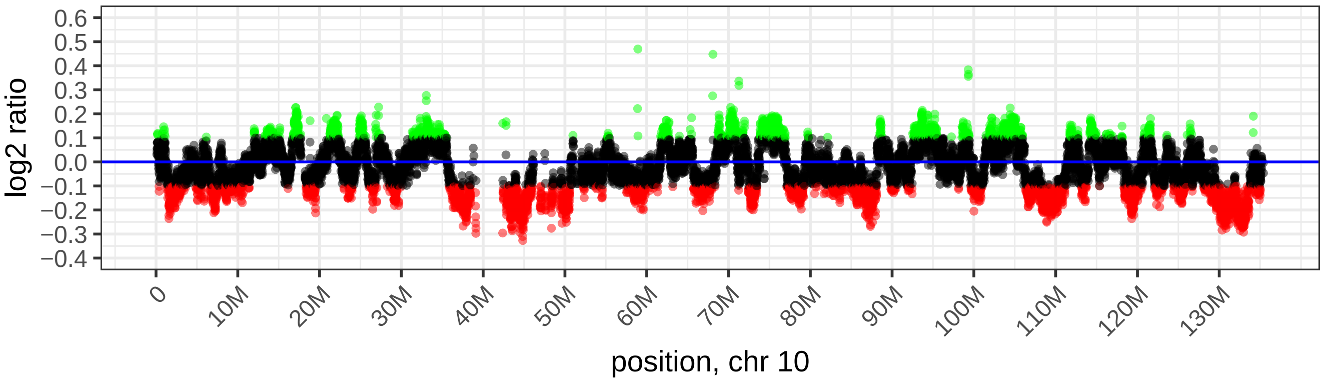

2d_C window: 25kb threshold: 0.1

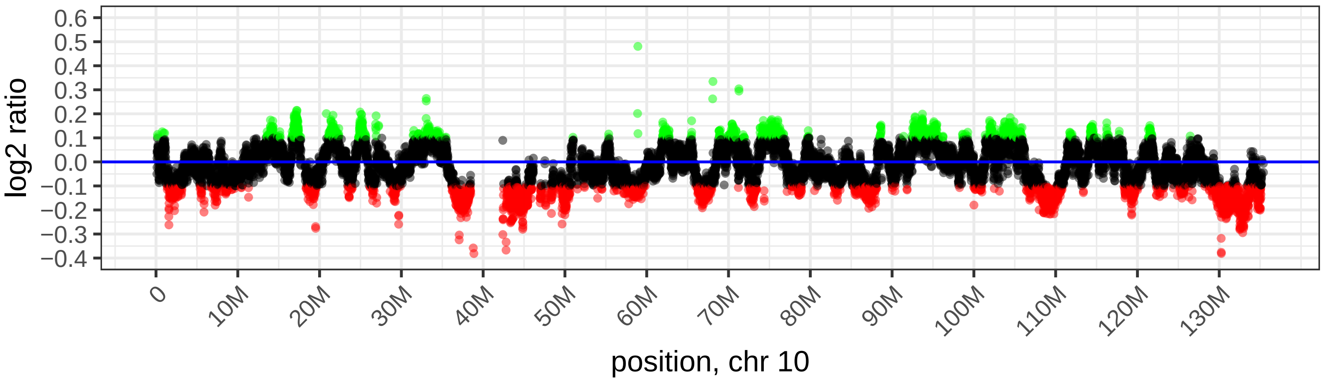

2d_Diff window: 25kb threshold: 0.1

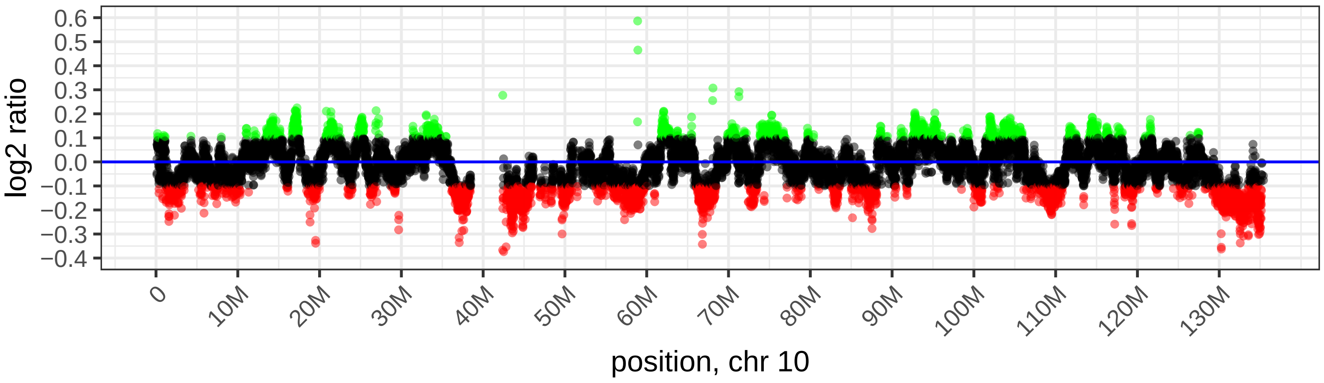

hMSC window: 25kb threshold: 0.1

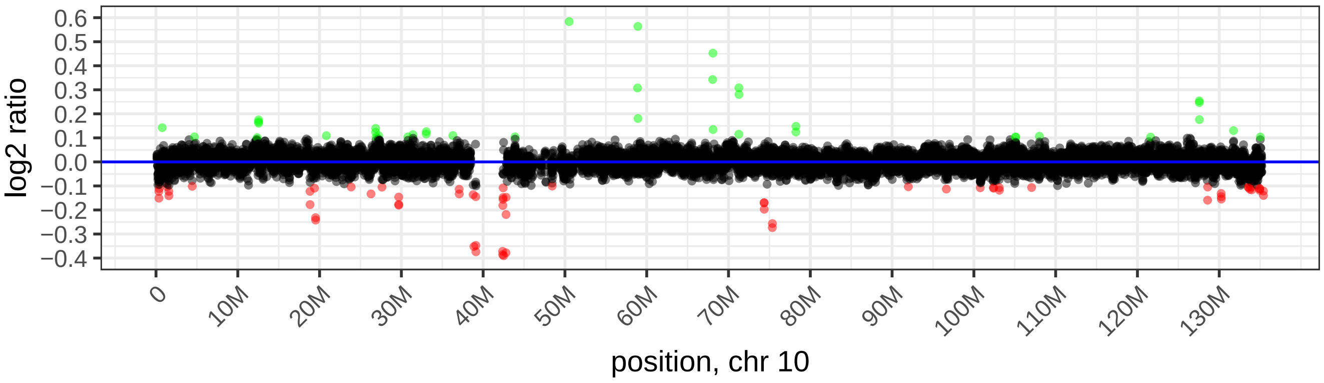

hMSC_aCGH window: 25kb threshold: 0.1

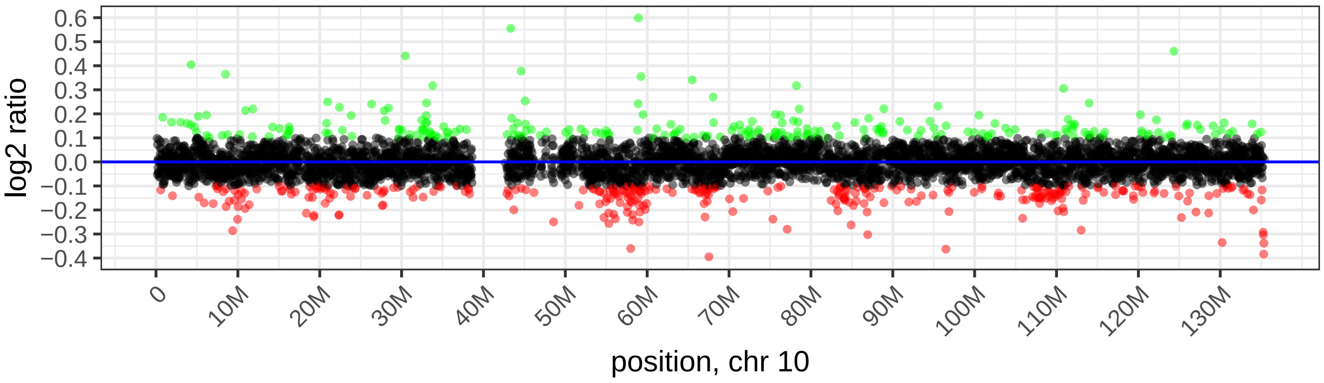

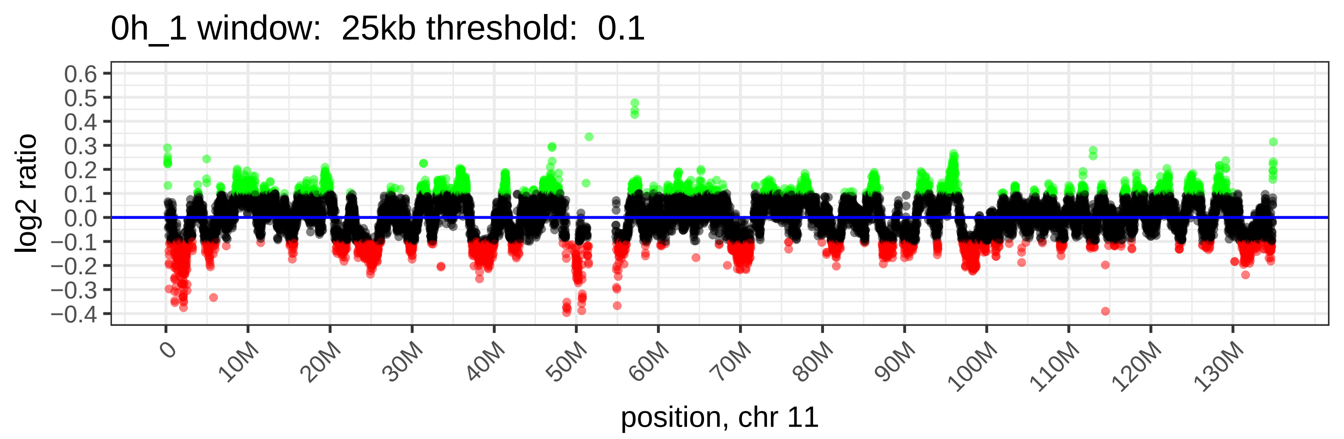
0h_1 window: 25kb threshold: 0.1
log2 ratio
0.6
0.5
0.4
0.3
0.2
0.1
0.0
-0.1
-0.2
-0.3
-0.4
0
10M
20M
30M
40M
50M
60M
70M
80M
90M
100M
110M
120M
130M
position, chr 11

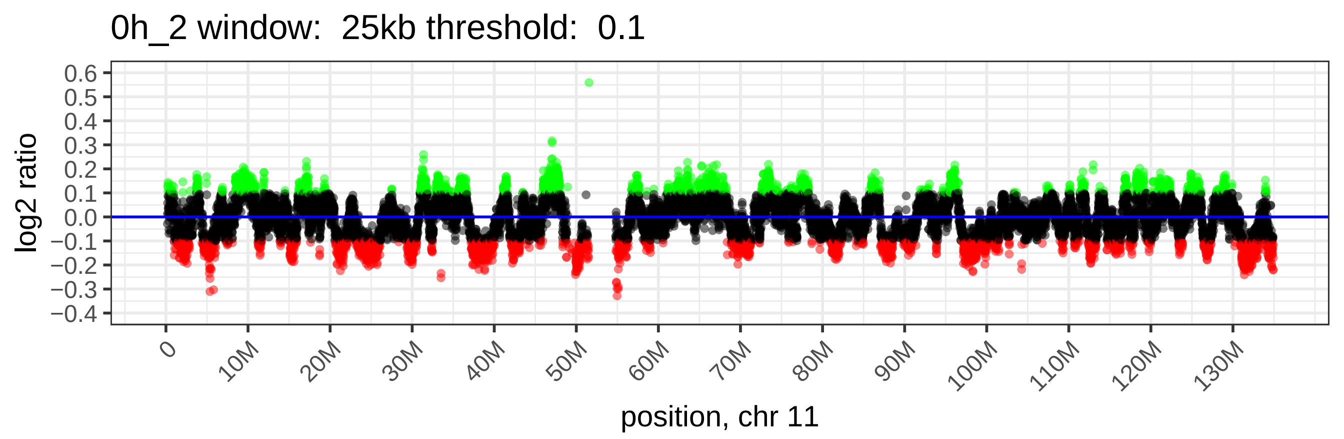
0h_2 window: 25kb threshold: 0.1
log2 ratio
0.6
0.5
0.4
0.3
0.2
0.1
0.0
-0.1
-0.2
-0.3
-0.4
0
10M
20M
30M
40M
50M
60M
70M
80M
90M
100M
110M
120M
130M
position, chr 11

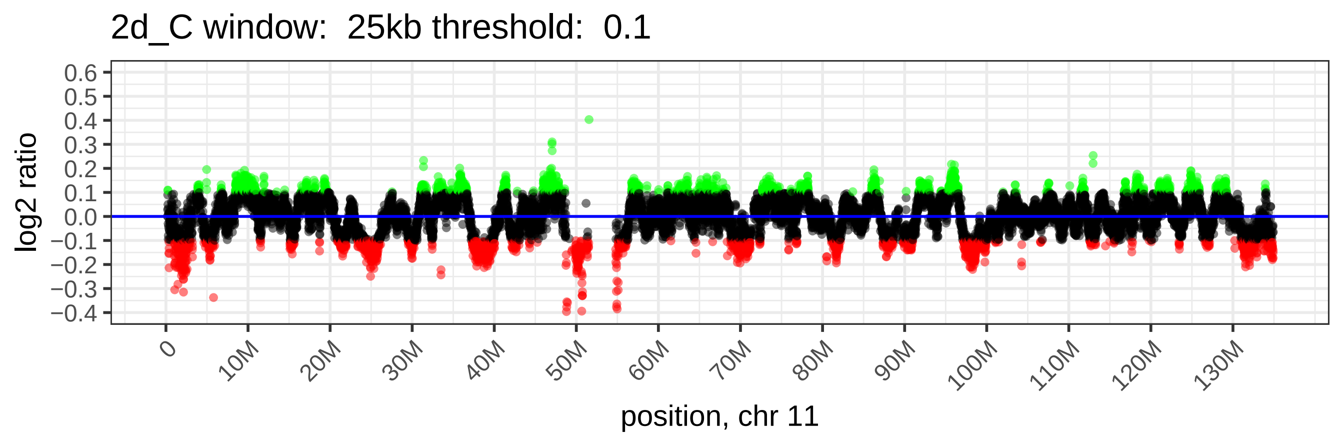
2d_C window: 25kb threshold: 0.1
log2 ratio
0.6
0.5
0.4
0.3
0.2
0.1
0.0
-0.1
-0.2
-0.3
-0.4
0
10M
20M
30M
40M
50M
60M
70M
80M
90M
100M
110M
120M
130M
position, chr 11

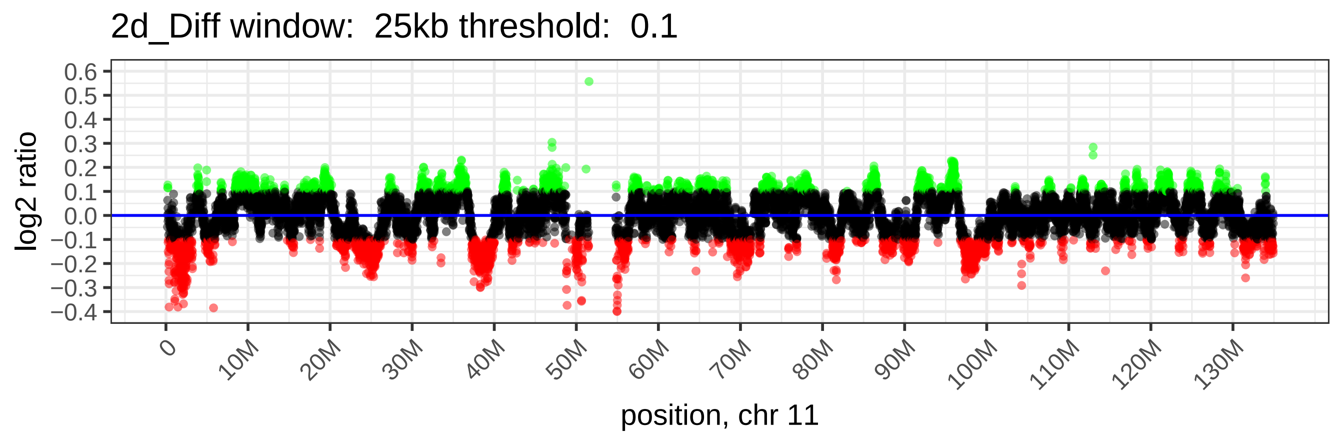
2d_Diff window: 25kb threshold: 0.1
log2 ratio
0.6
0.5
0.4
0.3
0.2
0.1
0.0
-0.1
-0.2
-0.3
-0.4
0
10M
20M
30M
40M
50M
60M
70M
80M
90M
100M
110M
120M
130M
position, chr 11

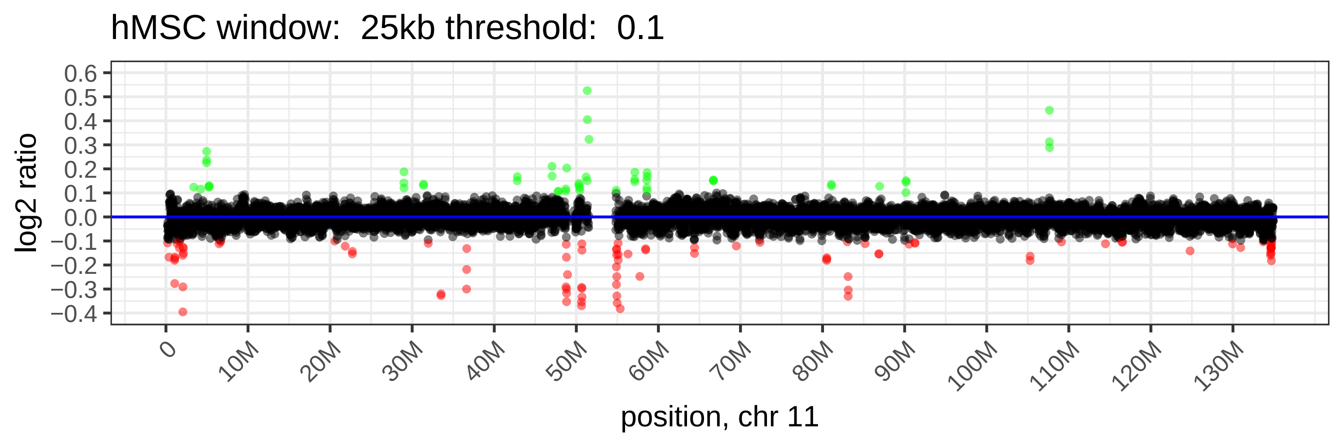
hMSC window: 25kb threshold: 0.1
log2 ratio
0.6
0.5
0.4
0.3
0.2
0.1
0.0
-0.1
-0.2
-0.3
-0.4
0
10M
20M
30M
40M
50M
60M
70M
80M
90M
100M
110M
120M
130M
position, chr 11

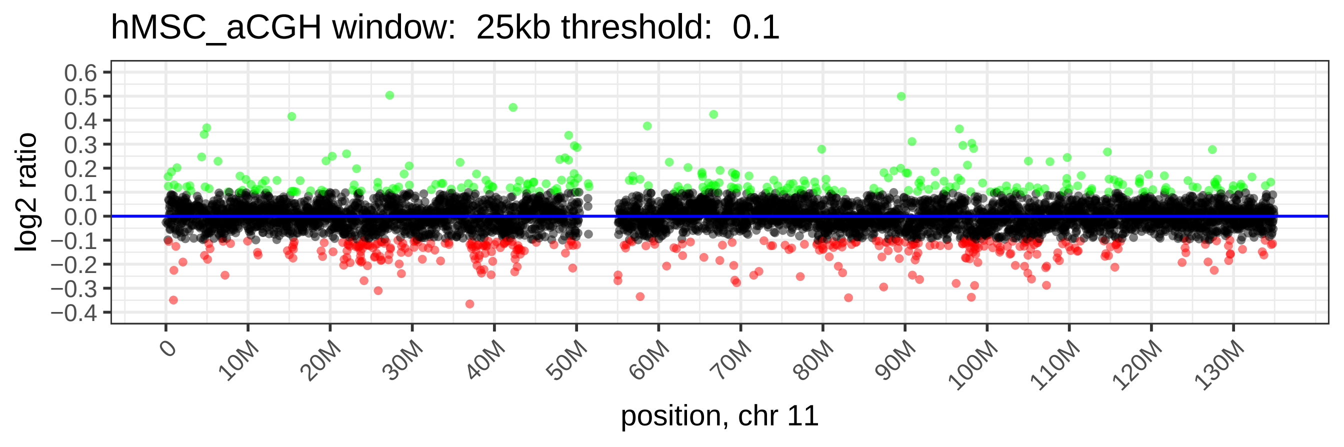
hMSC_aCGH window: 25kb threshold: 0.1
log2 ratio
0.6
0.5
0.4
0.3
0.2
0.1
0.0
-0.1
-0.2
-0.3
-0.4
0
10M
20M
30M
40M
50M
60M
70M
80M
90M
100M
110M
120M
130M
position, chr 11

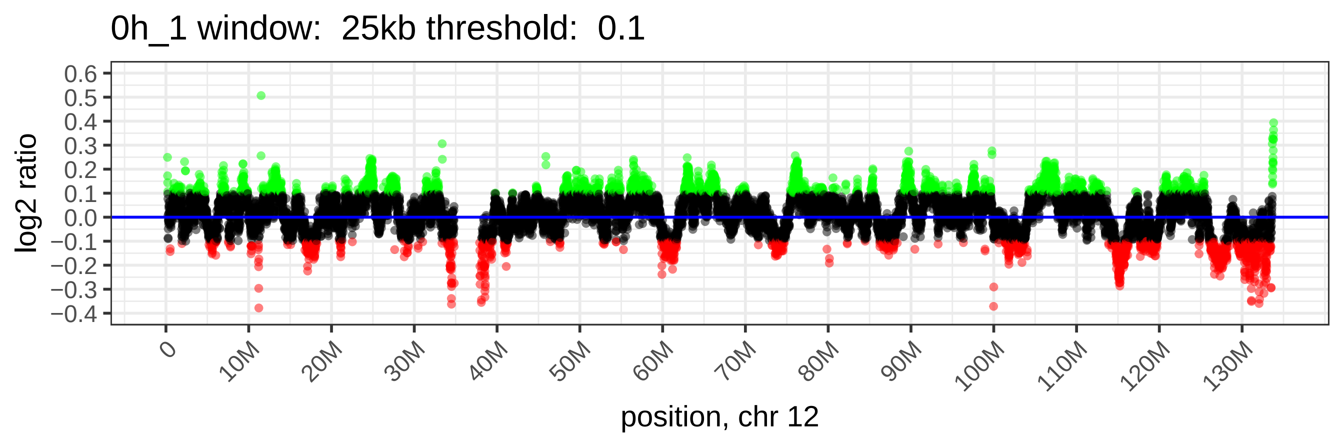
0h_1 window: 25kb threshold: 0.1
log2 ratio
0.6
0.5
0.4
0.3
0.2
0.1
0.0
-0.1
-0.2
-0.3
-0.4
0
10M
20M
30M
40M
50M
60M
70M
80M
90M
100M
110M
120M
130M
position, chr 12

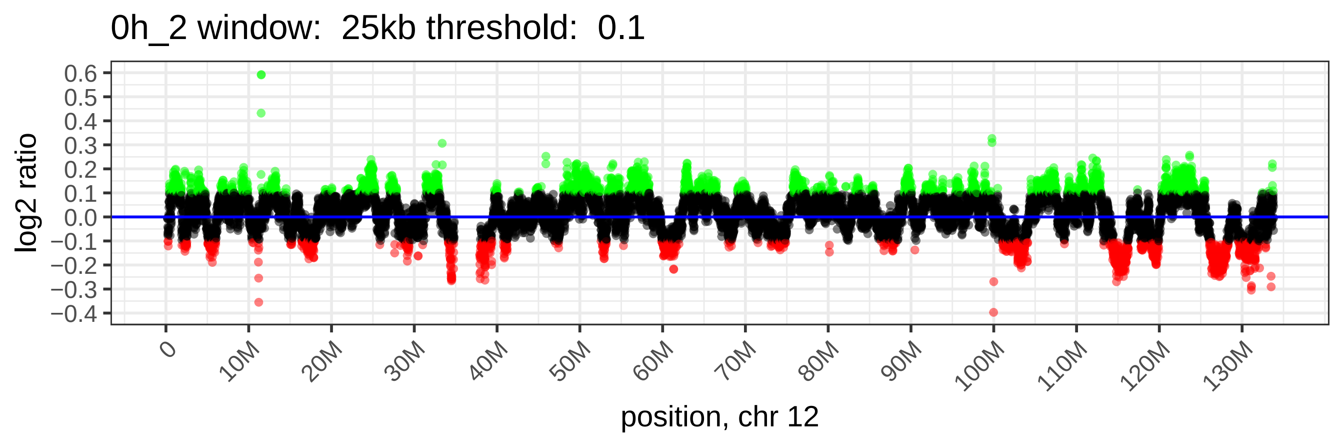
0h_2 window: 25kb threshold: 0.1
log2 ratio
0.6
0.5
0.4
0.3
0.2
0.1
0.0
-0.1
-0.2
-0.3
-0.4
0
10M
20M
30M
40M
50M
60M
70M
80M
90M
100M
110M
120M
130M
position, chr 12

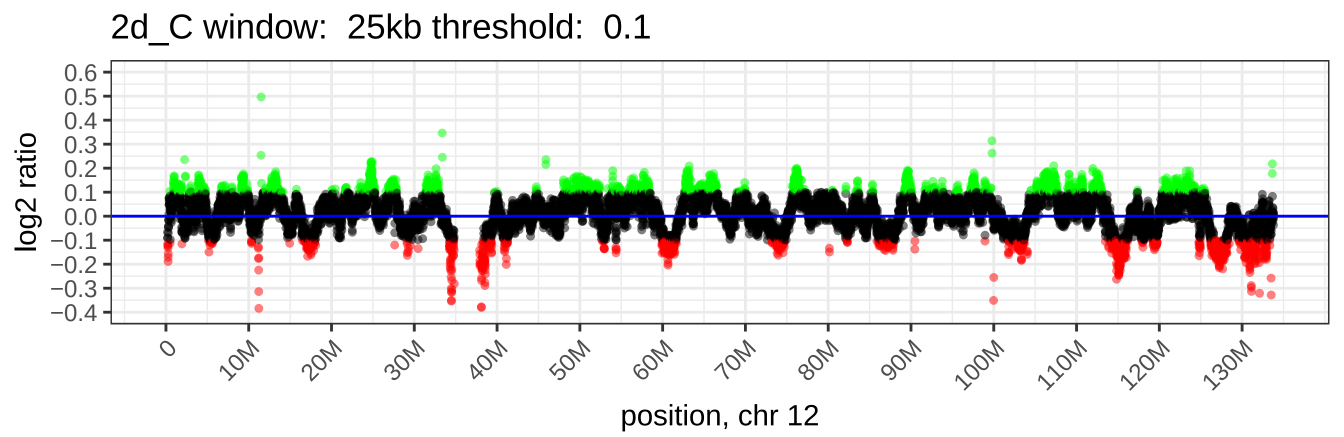
2d_C window: 25kb threshold: 0.1
log2 ratio
0.6
0.5
0.4
0.3
0.2
0.1
0.0
-0.1
-0.2
-0.3
-0.4
0
10M
20M
30M
40M
50M
60M
70M
80M
90M
100M
110M
120M
130M
position, chr 12

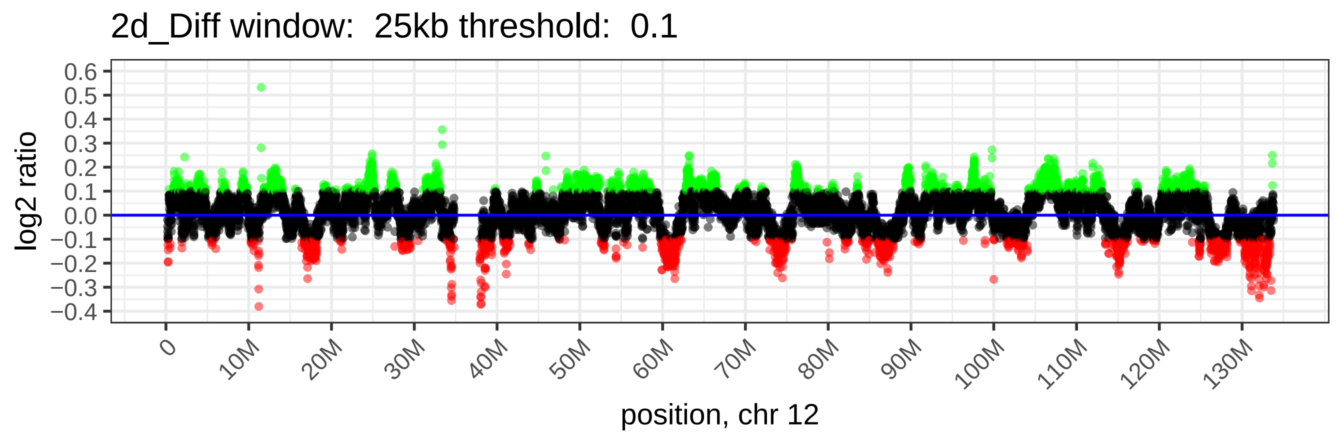
2d_Diff window: 25kb threshold: 0.1
log2 ratio
0.6
0.5
0.4
0.3
0.2
0.1
0.0
-0.1
-0.2
-0.3
-0.4
0
10M
20M
30M
40M
50M
60M
70M
80M
90M
100M
110M
120M
130M
position, chr 12

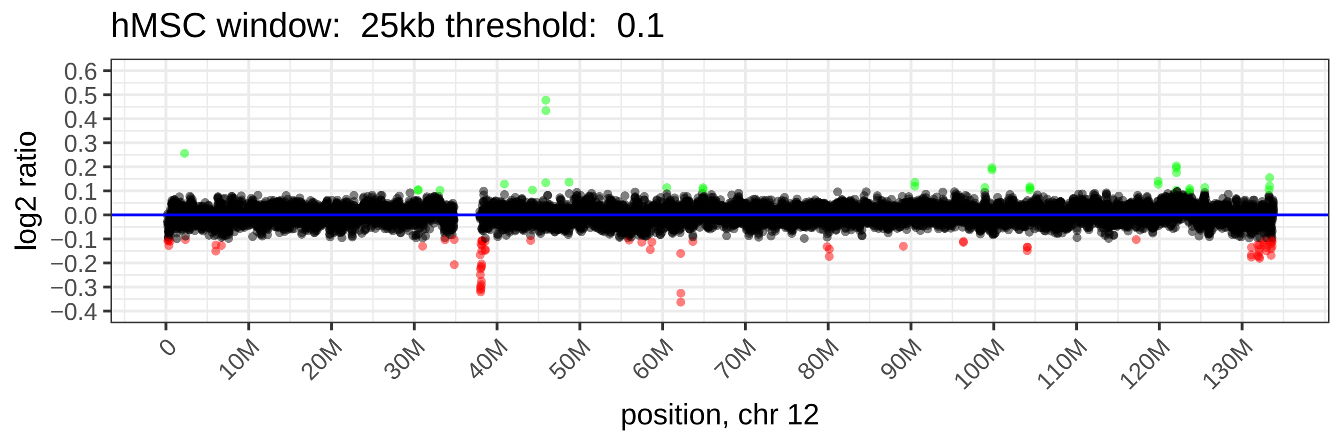
hMSC window: 25kb threshold: 0.1
log2 ratio
0.6
0.5
0.4
0.3
0.2
0.1
0.0
-0.1
-0.2
-0.3
-0.4
0
10M
20M
30M
40M
50M
60M
70M
80M
90M
100M
110M
120M
130M
position, chr 12

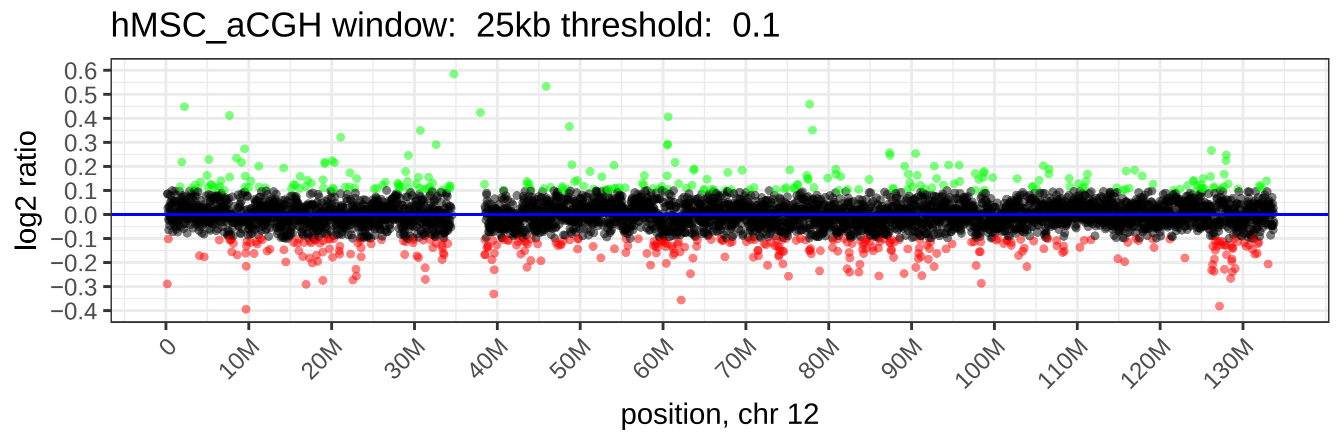
hMSC_aCGH window: 25kb threshold: 0.1
log2 ratio
0.6
0.5
0.4
0.3
0.2
0.1
0.0
-0.1
-0.2
-0.3
-0.4
0
10M
20M
30M
40M
50M
60M
70M
80M
90M
100M
110M
120M
130M
position, chr 12

0h_1 window: 25kb threshold: 0.1

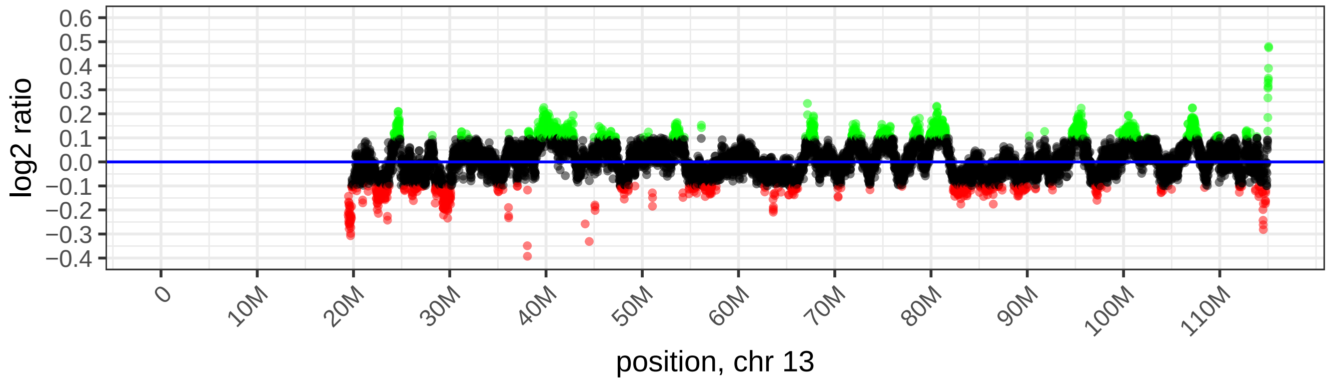

0h_2 window: 25kb threshold: 0.1

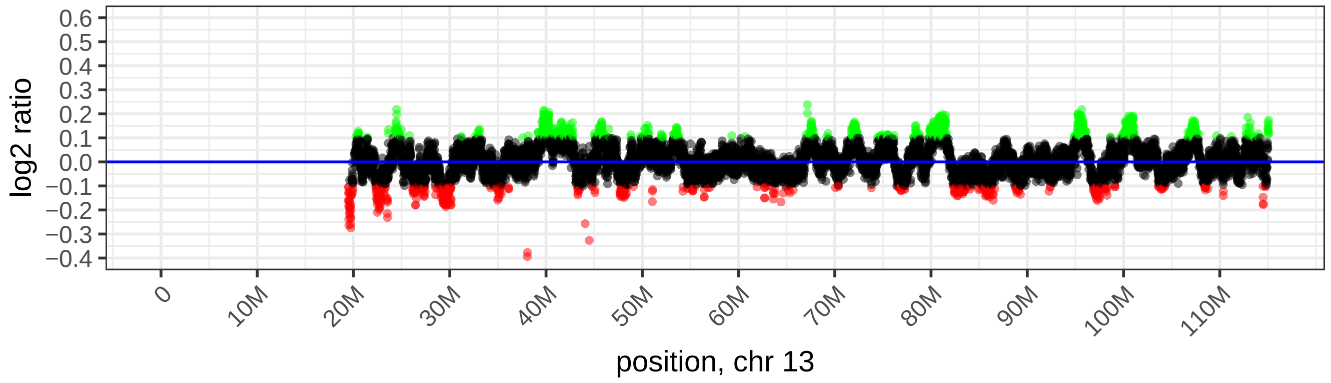

2d_C window: 25kb threshold: 0.1

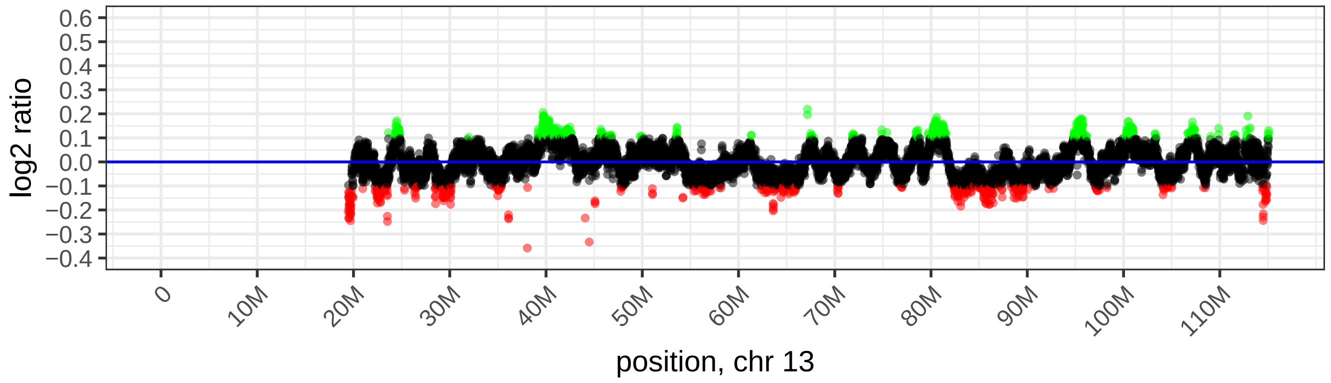

2d_Diff window: 25kb threshold: 0.1

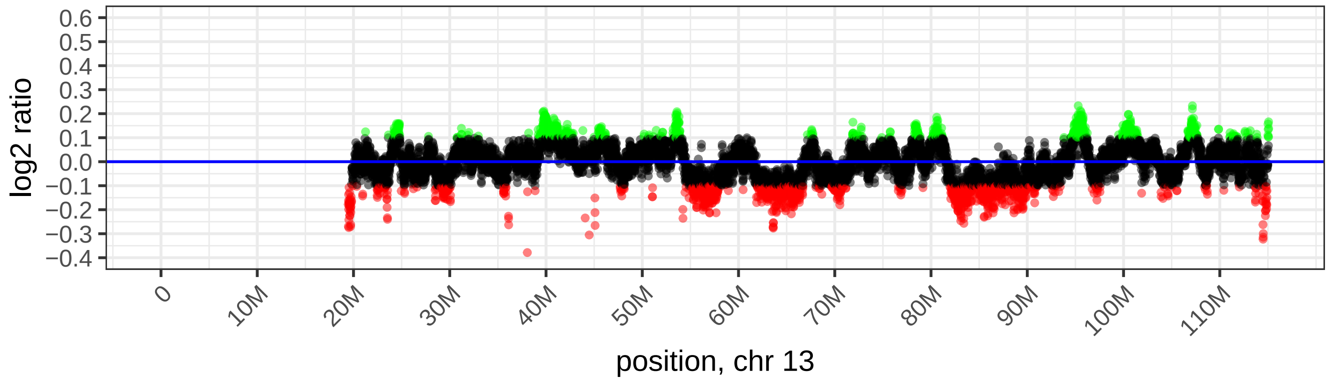

hMSC window: 25kb threshold: 0.1

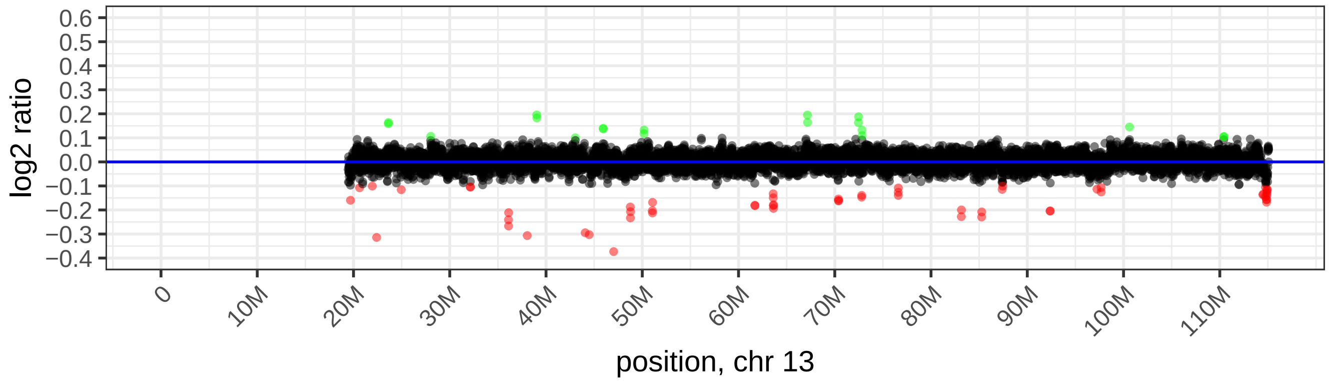

hMSC_aCGH window: 25kb threshold: 0.1

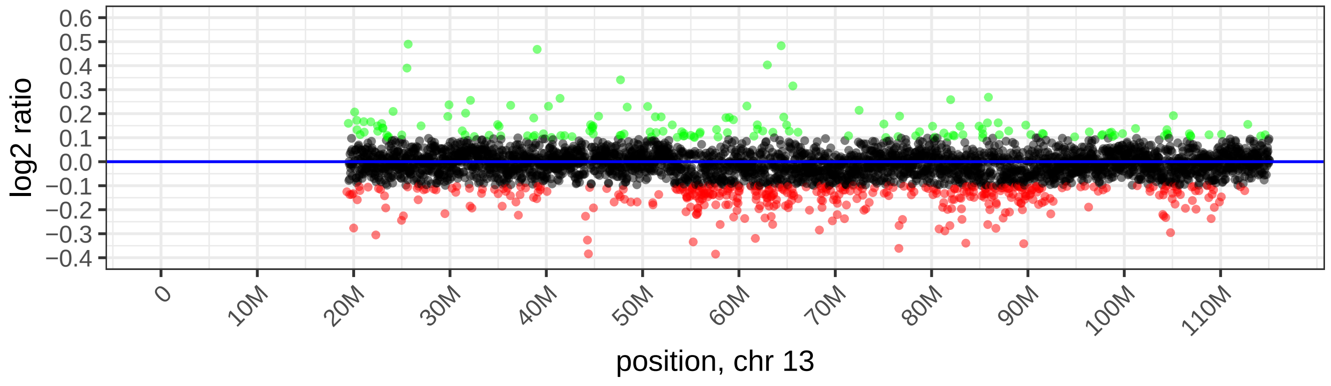

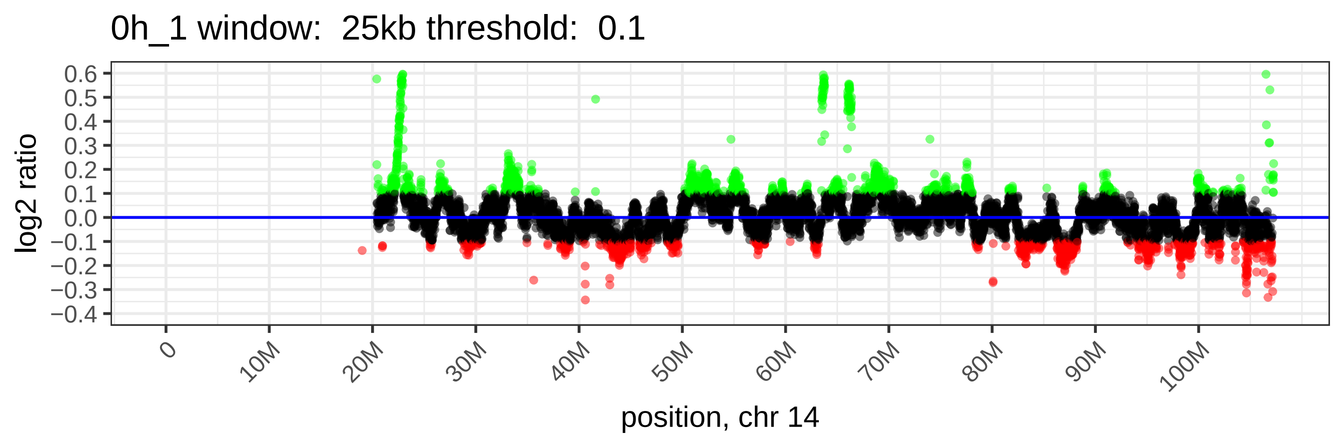
0h_1 window: 25kb threshold: 0.1
log2 ratio
0.6
0.5
0.4
0.3
0.2
0.1
0.0
-0.1
-0.2
-0.3
-0.4
0
10M
20M
30M
40M
50M
60M
70M
80M
90M
100M
position, chr 14

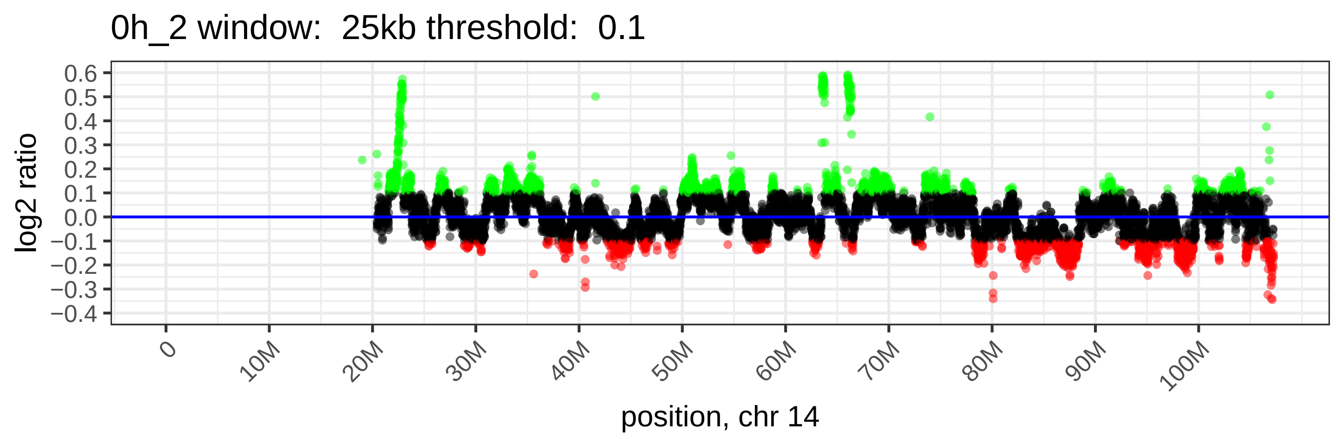
0h_2 window: 25kb threshold: 0.1
log2 ratio
0.6
0.5
0.4
0.3
0.2
0.1
0.0
-0.1
-0.2
-0.3
-0.4
0
10M
20M
30M
40M
50M
60M
70M
80M
90M
100M
position, chr 14

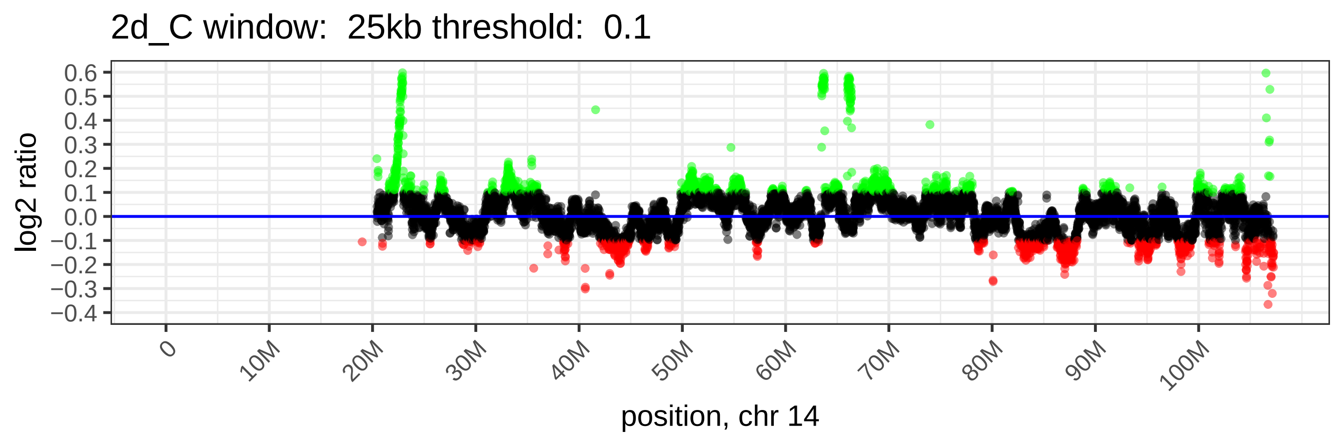
2d_C window: 25kb threshold: 0.1
log2 ratio
0.6
0.5
0.4
0.3
0.2
0.1
0.0
-0.1
-0.2
-0.3
-0.4
0
10M
20M
30M
40M
50M
60M
70M
80M
90M
100M
position, chr 14

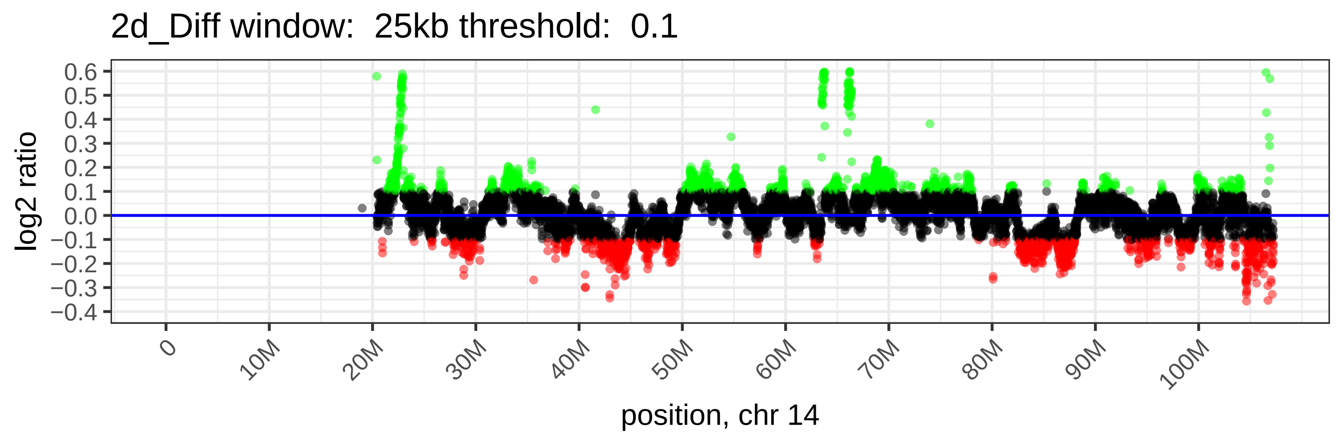
2d_Diff window: 25kb threshold: 0.1
log2 ratio
0.6
0.5
0.4
0.3
0.2
0.1
0.0
-0.1
-0.2
-0.3
-0.4
0
10M
20M
30M
40M
50M
60M
70M
80M
90M
100M
position, chr 14

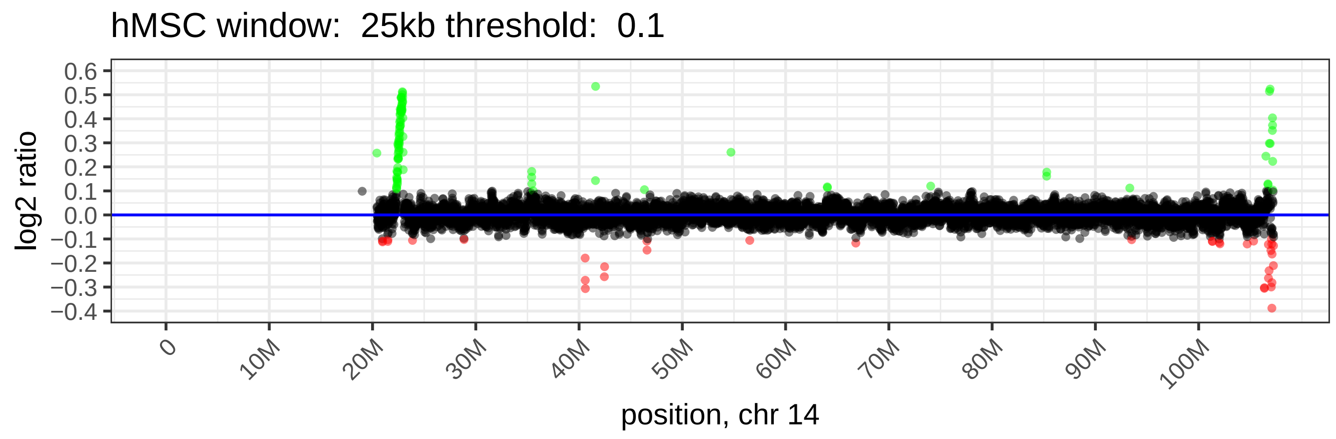
hMSC window: 25kb threshold: 0.1
log2 ratio
0.6
0.5
0.4
0.3
0.2
0.1
0.0
-0.1
-0.2
-0.3
-0.4
0
10M
20M
30M
40M
50M
60M
70M
80M
90M
100M
position, chr 14

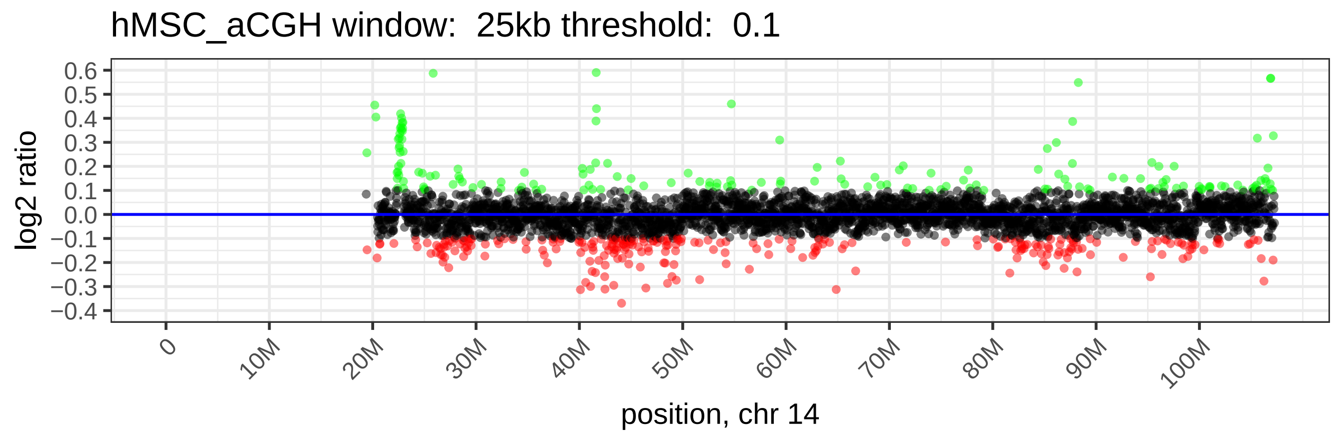
hMSC_aCGH window: 25kb threshold: 0.1
log2 ratio
0.6
0.5
0.4
0.3
0.2
0.1
0.0
-0.1
-0.2
-0.3
-0.4
0
10M
20M
30M
40M
50M
60M
70M
80M
90M
100M
position, chr 14

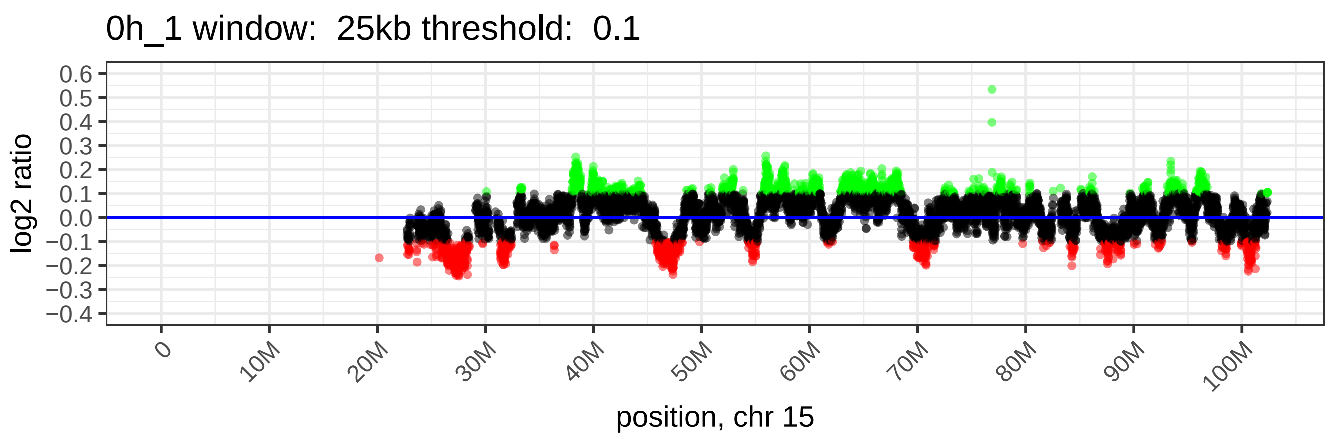
0h_1 window: 25kb threshold: 0.1
log2 ratio
position, chr 15

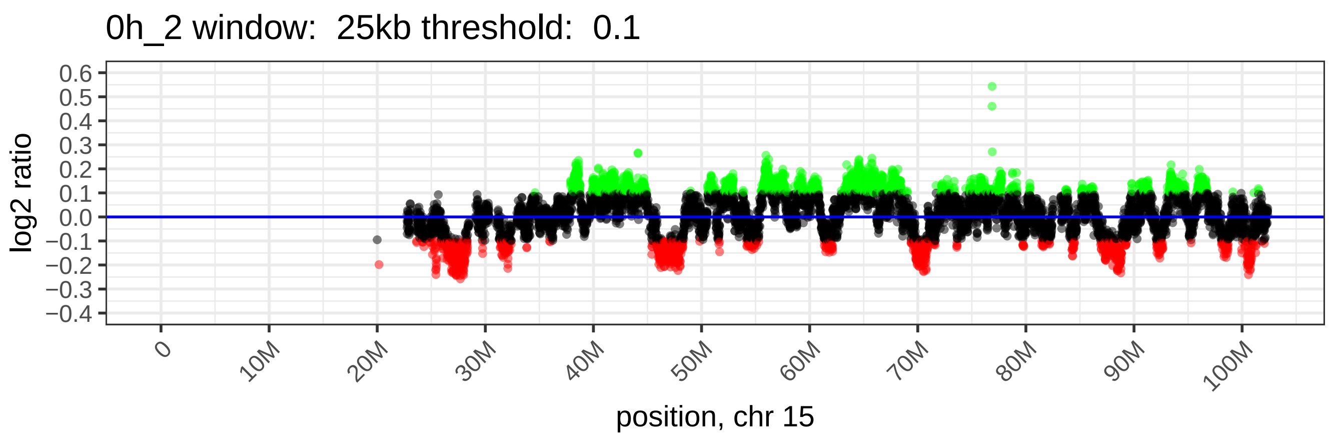
0h_2 window: 25kb threshold: 0.1
log2 ratio
position, chr 15

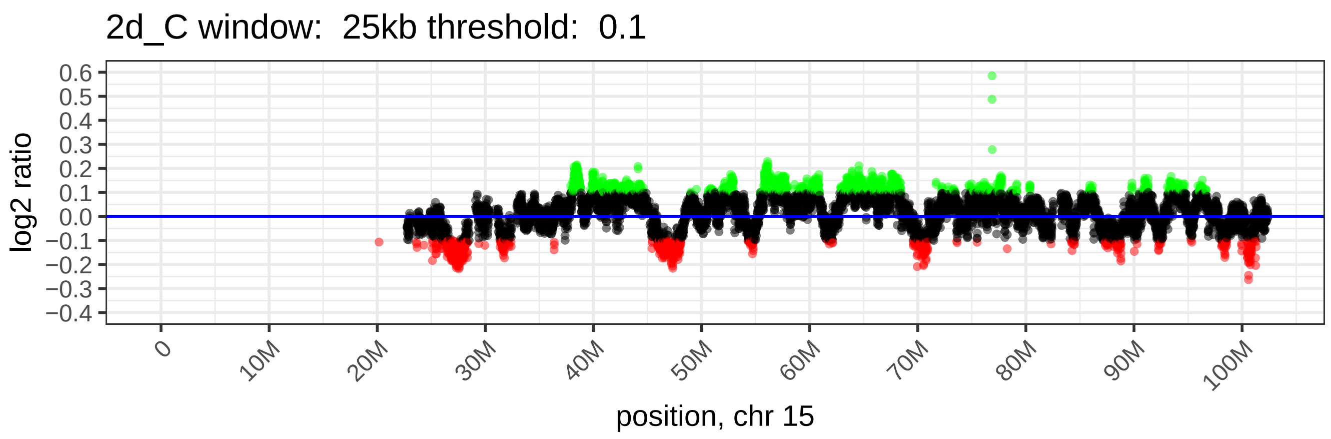
2d_C window: 25kb threshold: 0.1
log2 ratio
position, chr 15

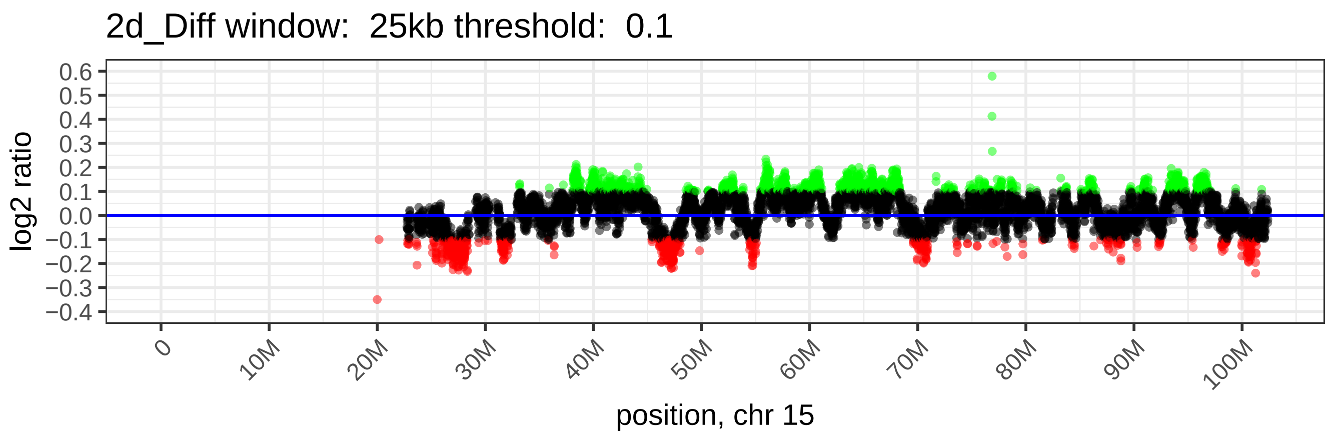
2d_Diff window: 25kb threshold: 0.1
log2 ratio
position, chr 15

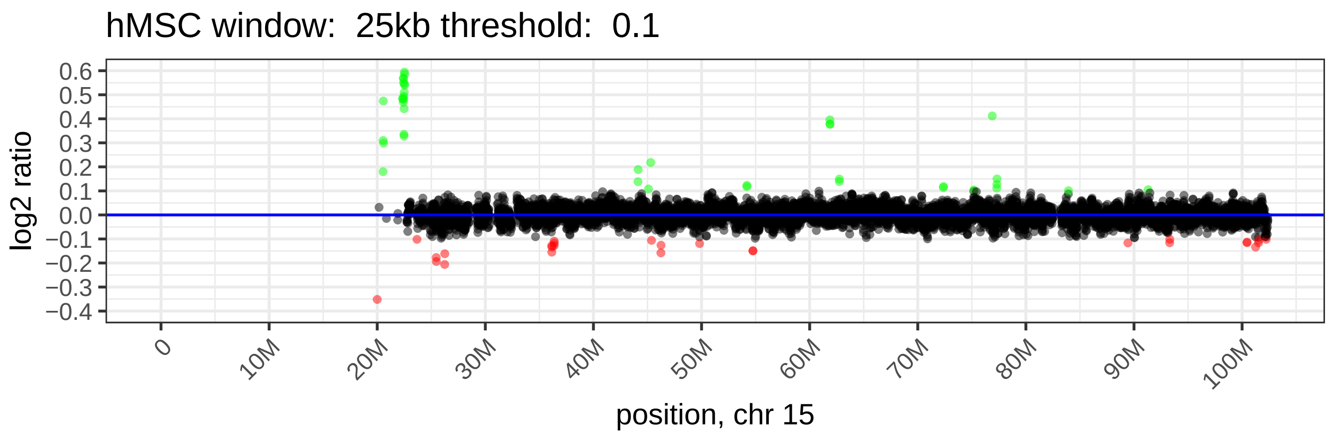
hMSC window: 25kb threshold: 0.1
log2 ratio
position, chr 15

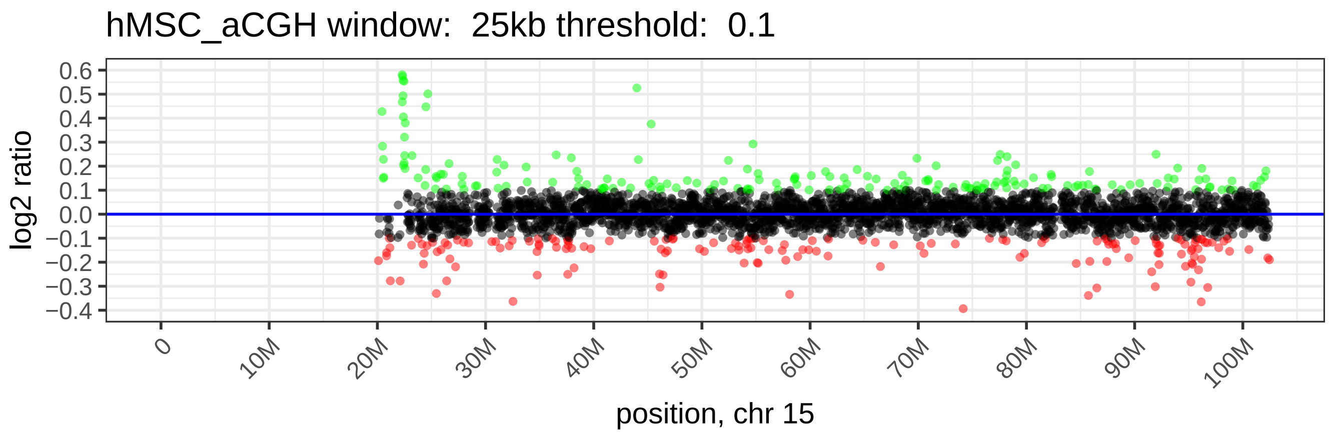
hMSC_aCGH window: 25kb threshold: 0.1
log2 ratio
position, chr 15

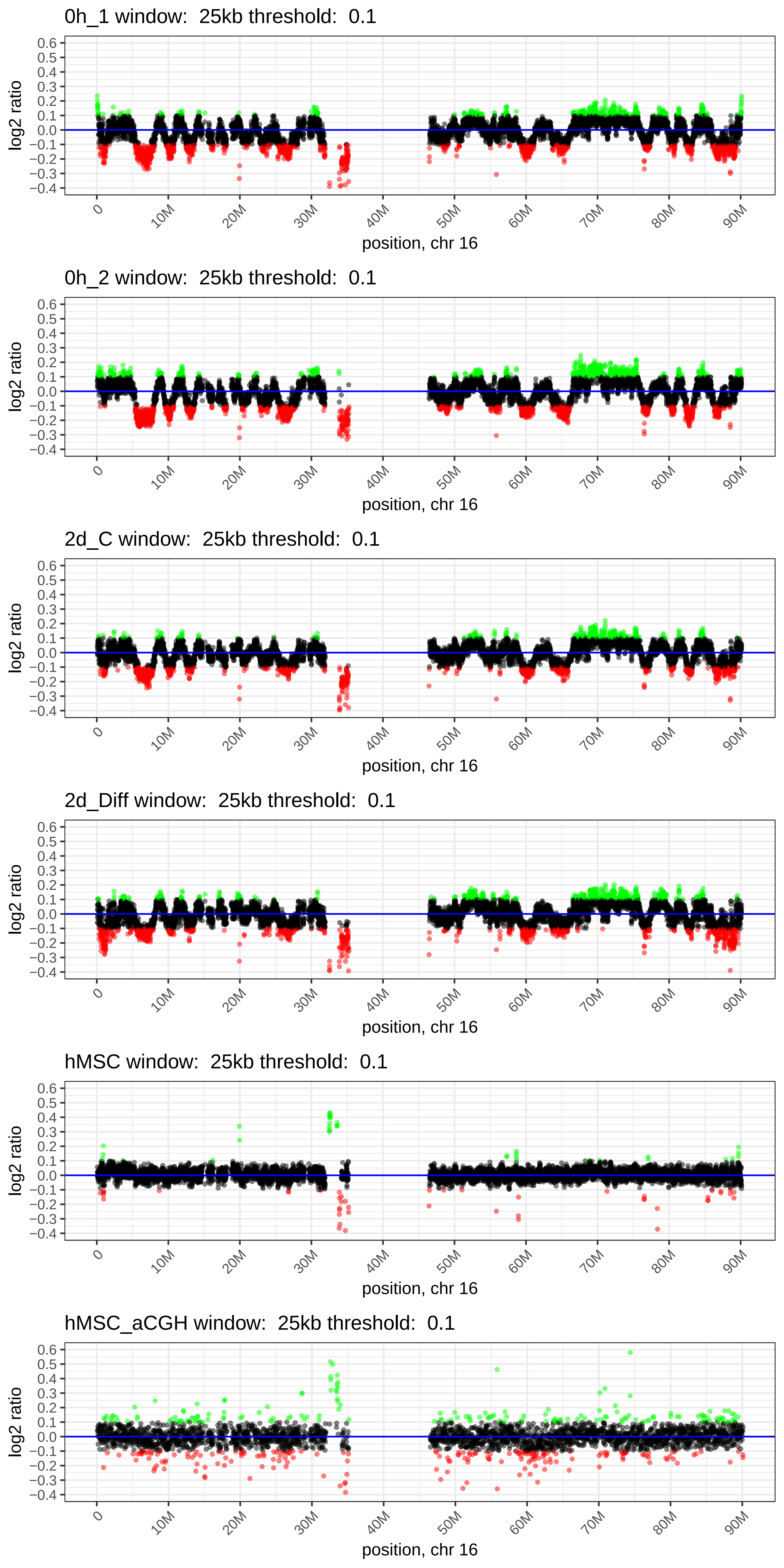
0h_1 window: 25kb threshold: 0.1
log2 ratio
position, chr 16
0h_2 window: 25kb threshold: 0.1
log2 ratio
position, chr 16
2d_C window: 25kb threshold: 0.1
log2 ratio
position, chr 16
2d_Diff window: 25kb threshold: 0.1
log2 ratio
position, chr 16
hMSC window: 25kb threshold: 0.1
log2 ratio
position, chr 16
hMSC_aCGH window: 25kb threshold: 0.1
log2 ratio
position, chr 16

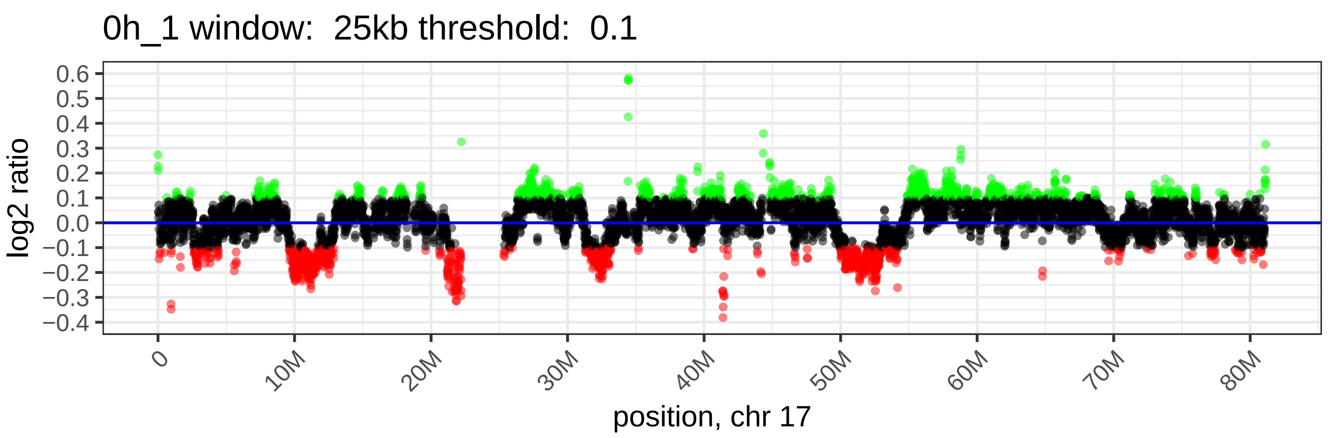
0h_1 window: 25kb threshold: 0.1
log2 ratio
0.6
0.5
0.4
0.3
0.2
0.1
0.0
-0.1
-0.2
-0.3
-0.4
0
10M
20M
30M
40M
50M
60M
70M
80M
position, chr 17

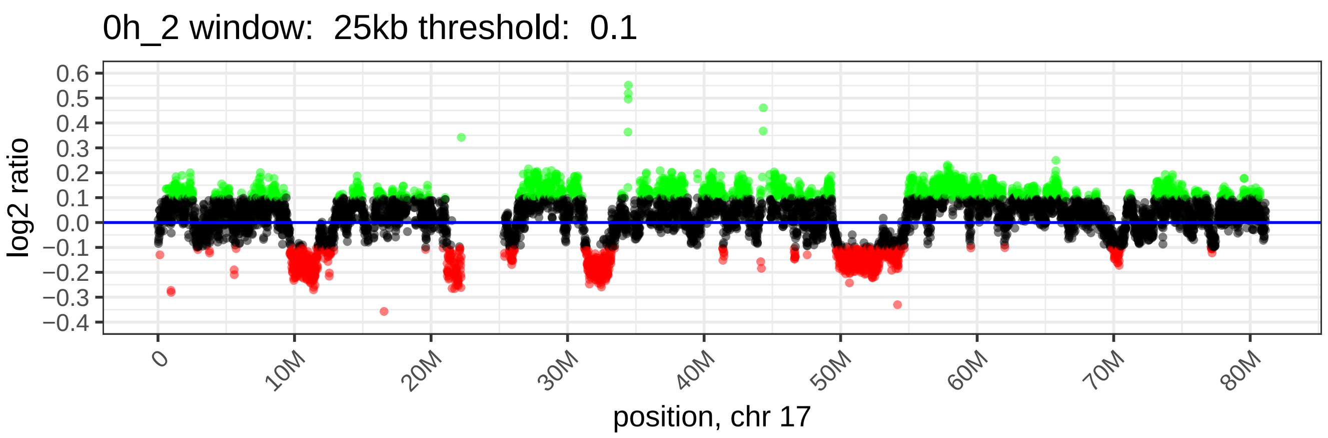
0h_2 window: 25kb threshold: 0.1
log2 ratio
0.6
0.5
0.4
0.3
0.2
0.1
0.0
-0.1
-0.2
-0.3
-0.4
0
10M
20M
30M
40M
50M
60M
70M
80M
position, chr 17

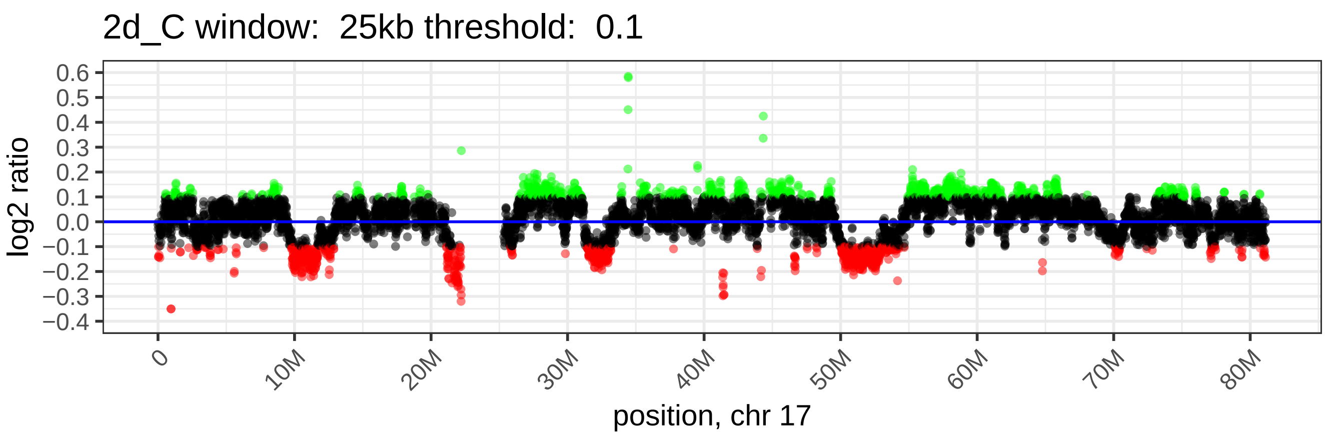
2d_C window: 25kb threshold: 0.1
log2 ratio
0.6
0.5
0.4
0.3
0.2
0.1
0.0
-0.1
-0.2
-0.3
-0.4
0
10M
20M
30M
40M
50M
60M
70M
80M
position, chr 17

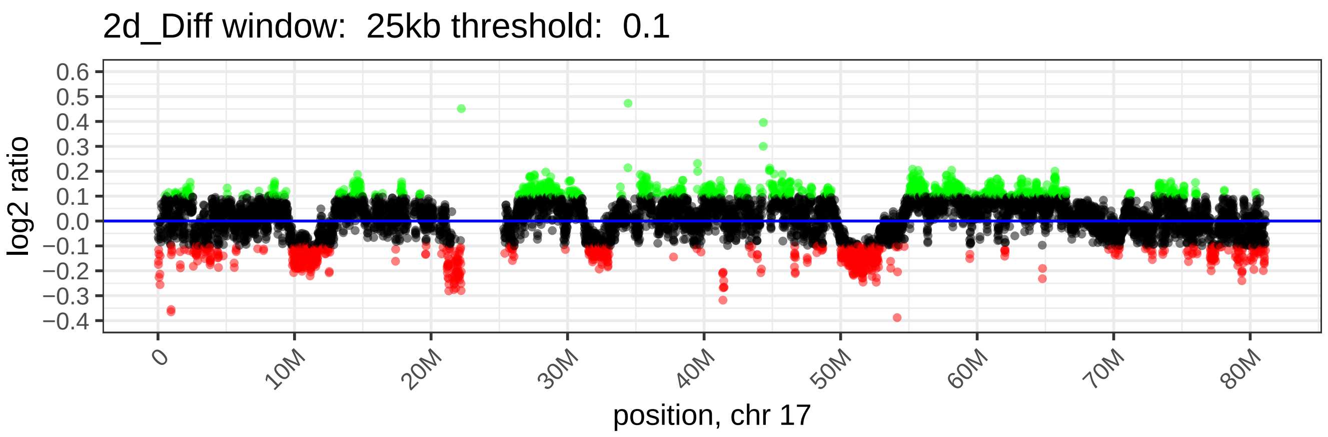
2d_Diff window: 25kb threshold: 0.1
log2 ratio
0.6
0.5
0.4
0.3
0.2
0.1
0.0
-0.1
-0.2
-0.3
-0.4
0
10M
20M
30M
40M
50M
60M
70M
80M
position, chr 17

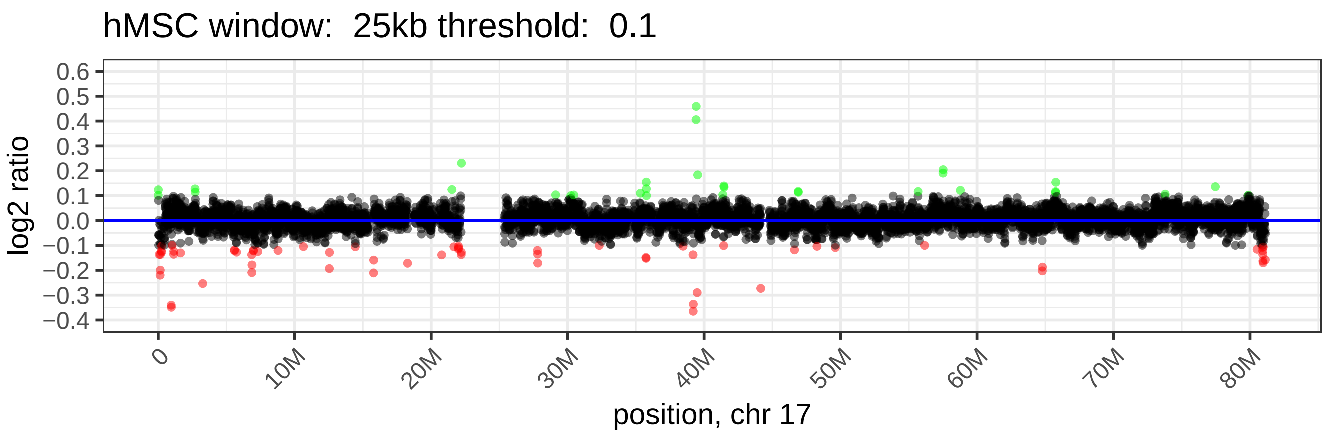
hMSC window: 25kb threshold: 0.1
log2 ratio
0.6
0.5
0.4
0.3
0.2
0.1
0.0
-0.1
-0.2
-0.3
-0.4
0
10M
20M
30M
40M
50M
60M
70M
80M
position, chr 17

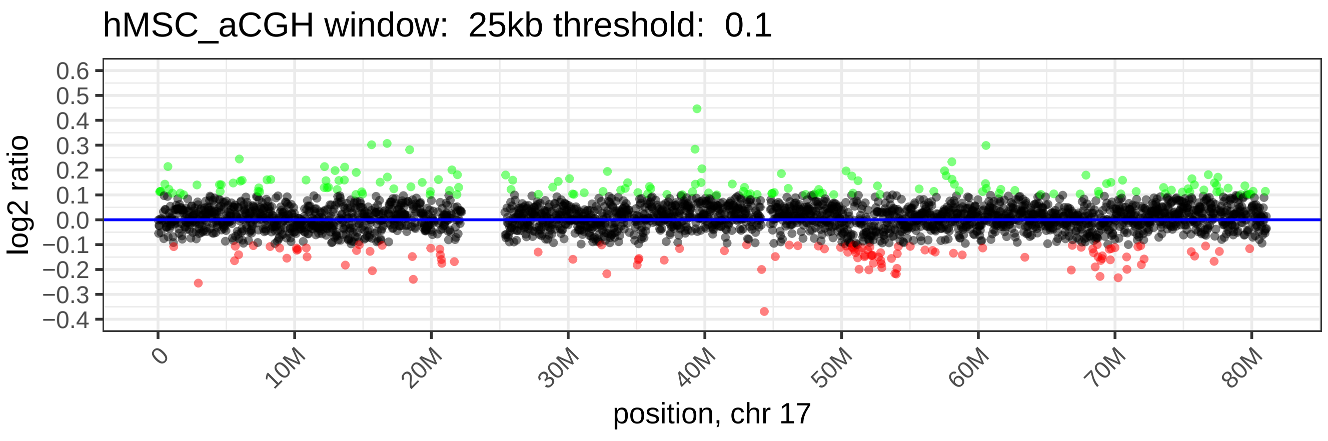
hMSC_aCGH window: 25kb threshold: 0.1
log2 ratio
0.6
0.5
0.4
0.3
0.2
0.1
0.0
-0.1
-0.2
-0.3
-0.4
0
10M
20M
30M
40M
50M
60M
70M
80M
position, chr 17

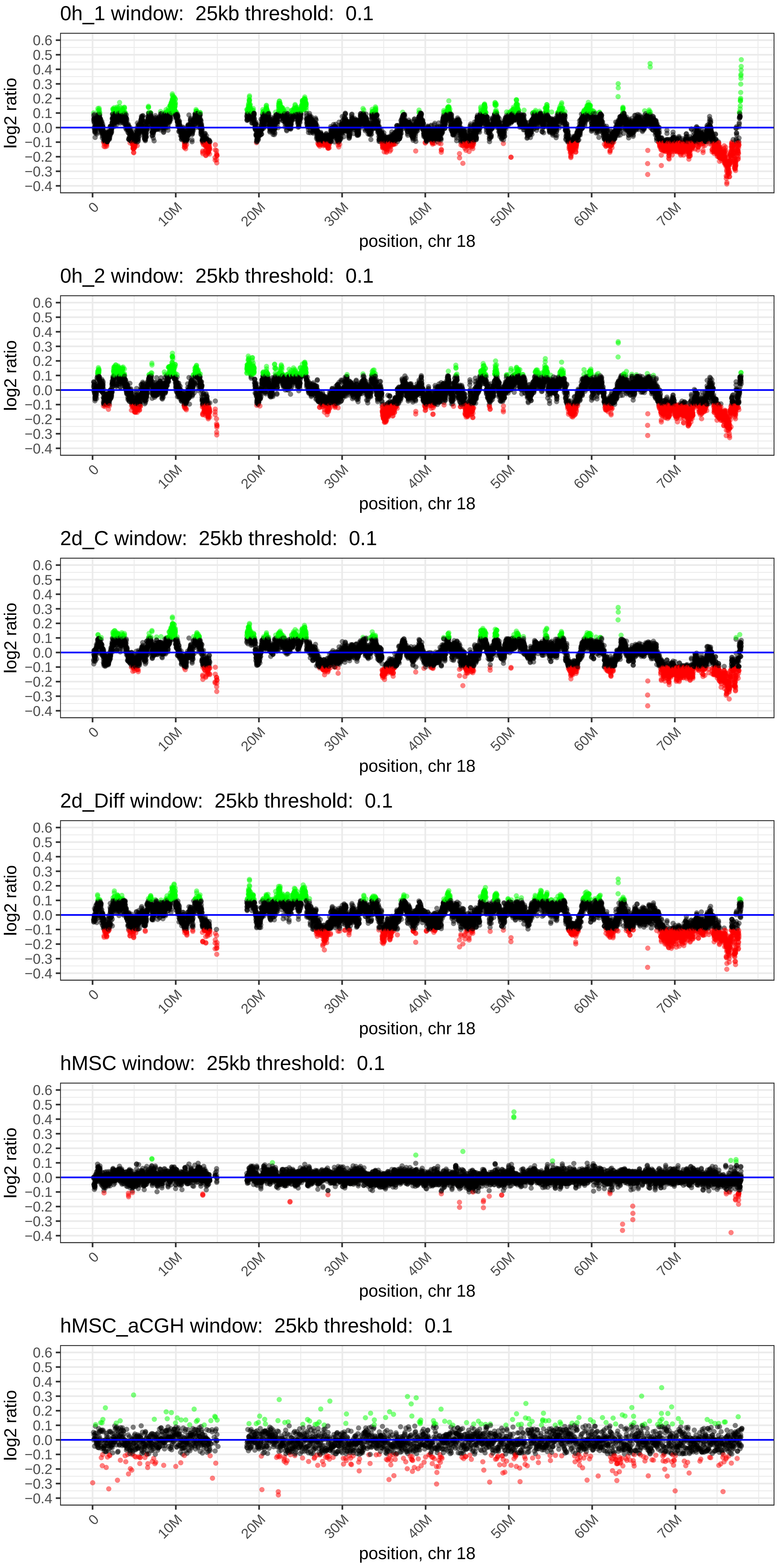
0h_1 window: 25kb threshold: 0.1
log2 ratio
0.6
0.5
0.4
0.3
0.2
0.1
0.0
-0.1
-0.2
-0.3
-0.4
0
10M
20M
30M
40M
50M
60M
70M
position, chr 18
0h_2 window: 25kb threshold: 0.1
log2 ratio
position, chr 18
2d_C window: 25kb threshold: 0.1
log2 ratio
position, chr 18
2d_Diff window: 25kb threshold: 0.1
log2 ratio
position, chr 18
hMSC window: 25kb threshold: 0.1
log2 ratio
position, chr 18
hMSC_aCGH window: 25kb threshold: 0.1
log2 ratio
position, chr 18

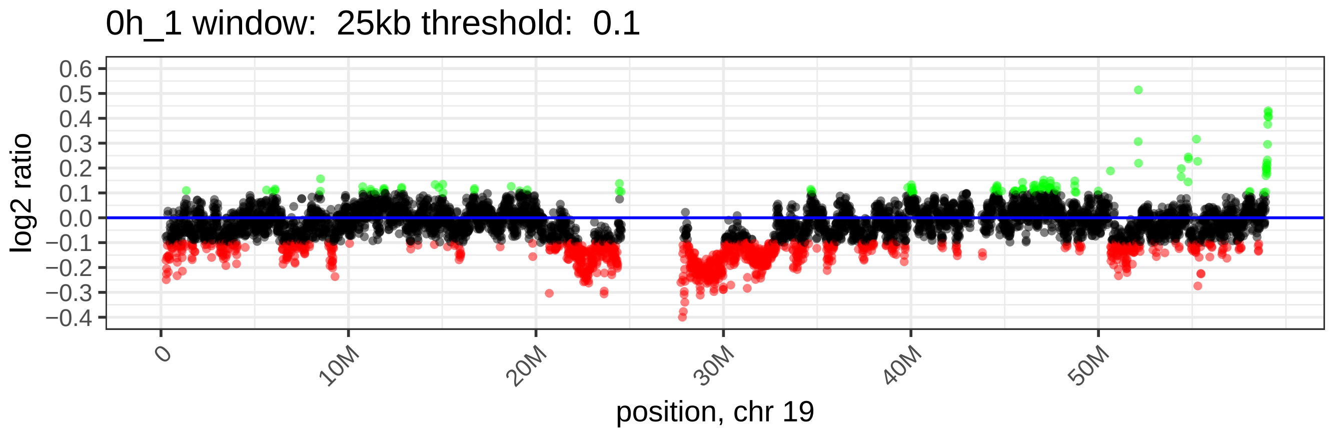
0h_1 window: 25kb threshold: 0.1
log2 ratio
position, chr 19

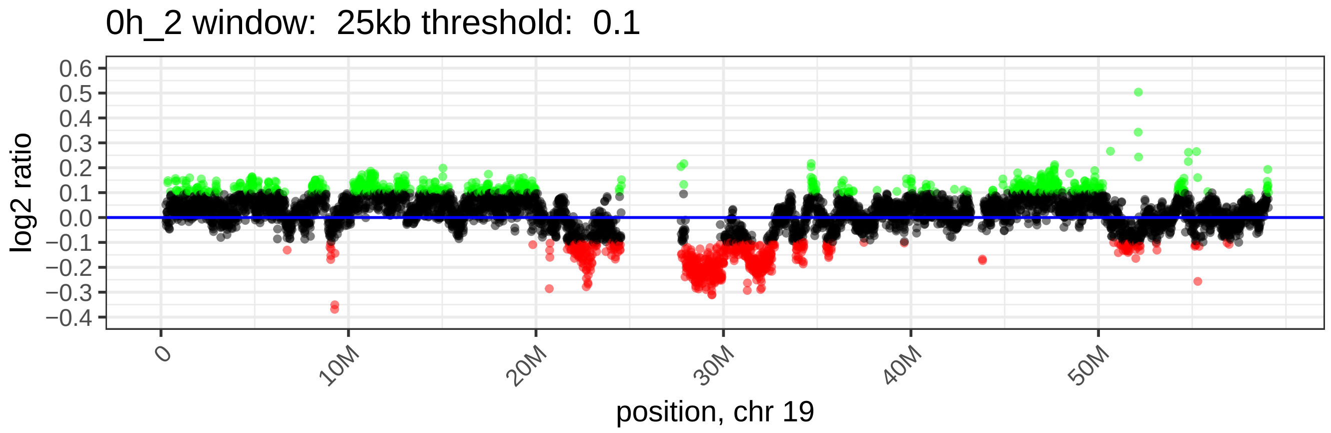
0h_2 window: 25kb threshold: 0.1
log2 ratio
position, chr 19

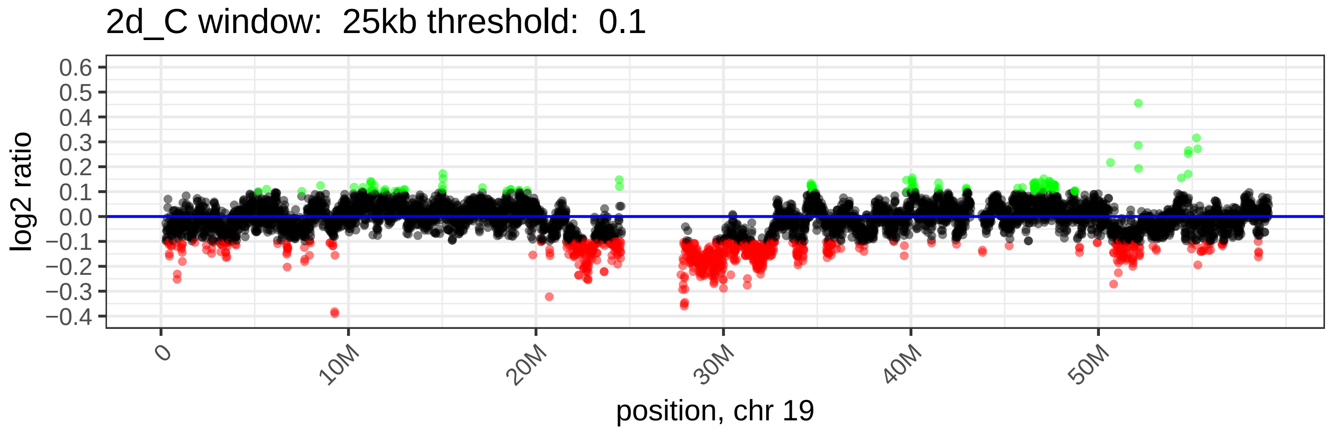
2d_C window: 25kb threshold: 0.1
log2 ratio
position, chr 19

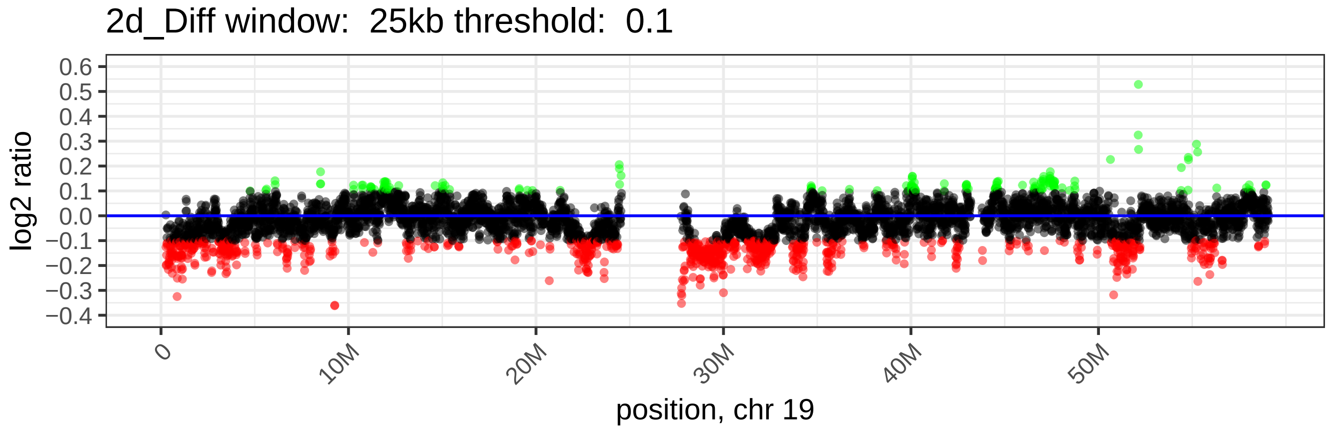
2d_Diff window: 25kb threshold: 0.1
log2 ratio
position, chr 19

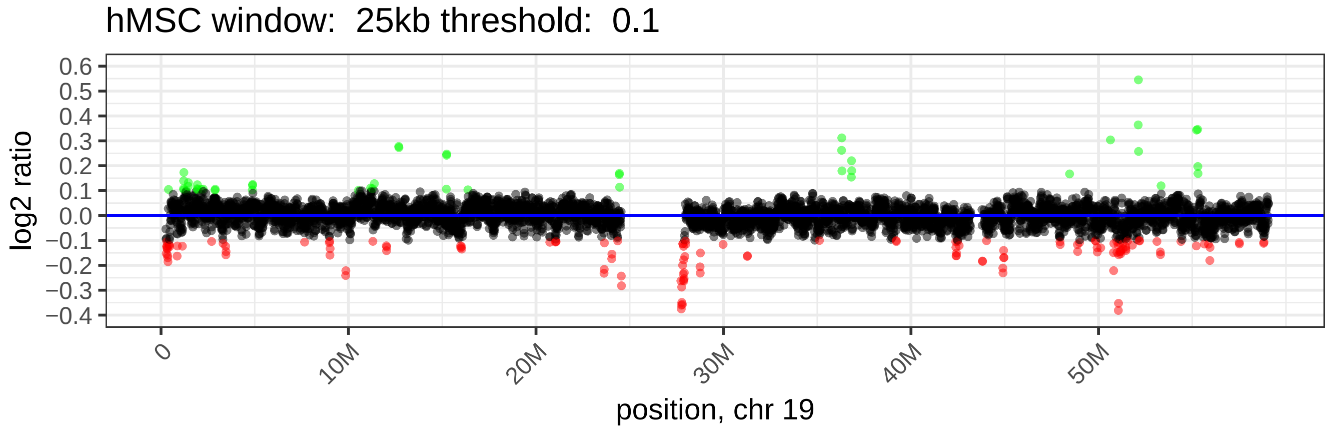
hMSC window: 25kb threshold: 0.1
log2 ratio
position, chr 19

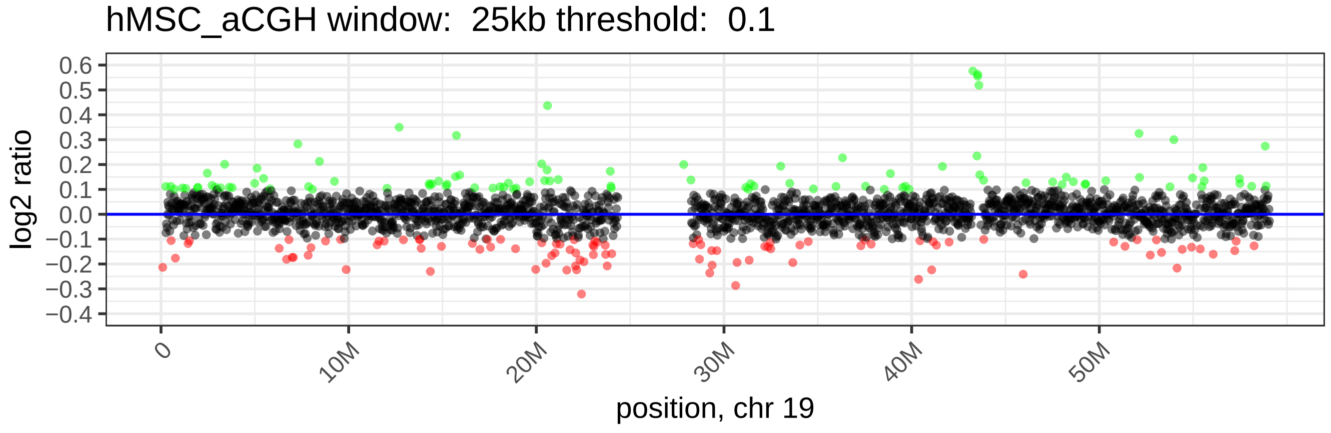
hMSC_aCGH window: 25kb threshold: 0.1
log2 ratio
position, chr 19

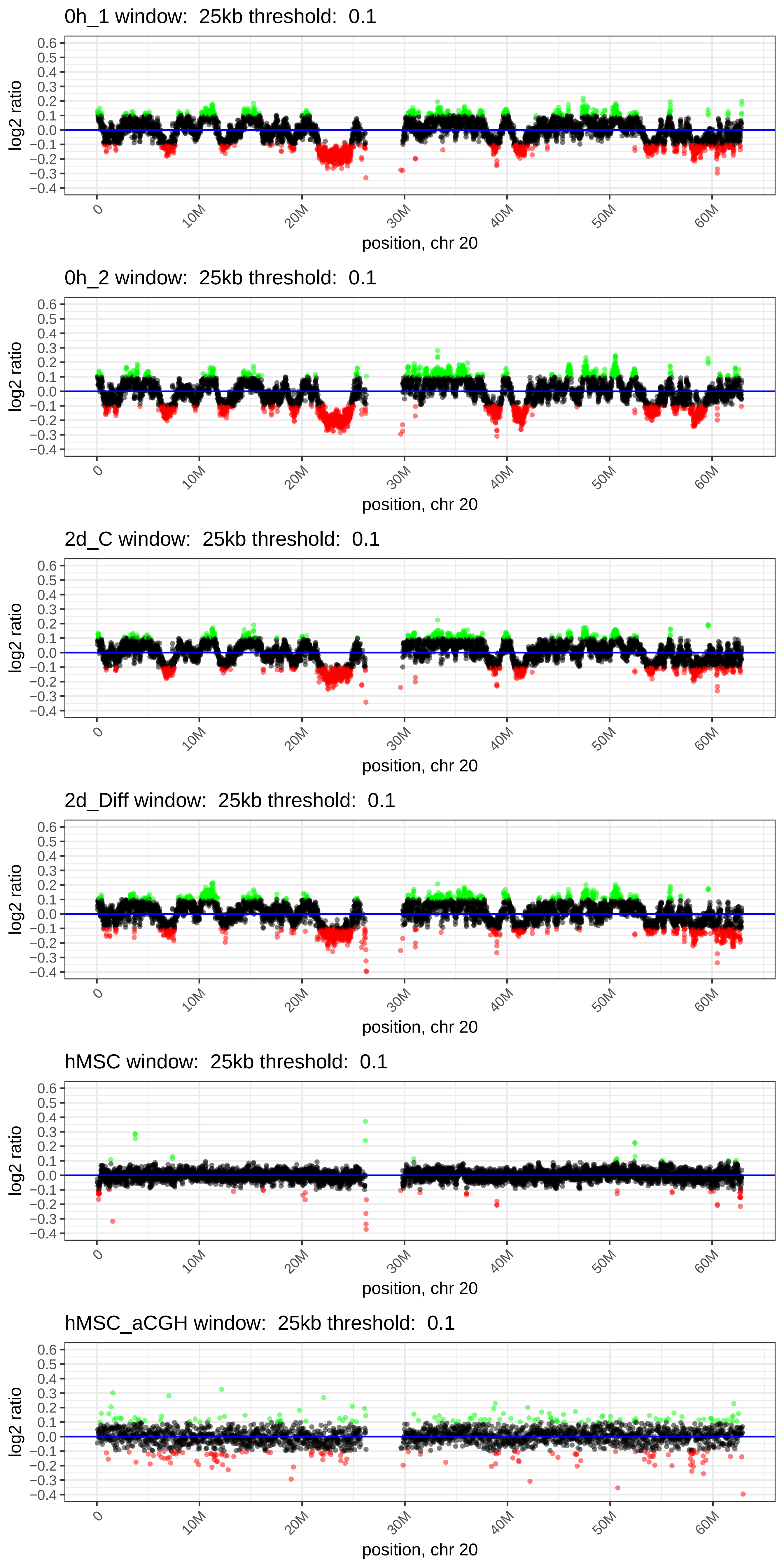

0h_1 window: 25kb threshold: 0.1
0h_2 window: 25kb threshold: 0.1
2d_C window: 25kb threshold: 0.1
2d_Diff window: 25kb threshold: 0.1
hMSC window: 25kb threshold: 0.1
hMSC_aCGH window: 25kb threshold: 0.1
log2 ratio
0.6
0.5
0.4
0.3
0.2
0.1
0.0
−0.1
−0.2
−0.3
−0.4
0
10M
20M
30M
40M
50M
60M
position, chr 20

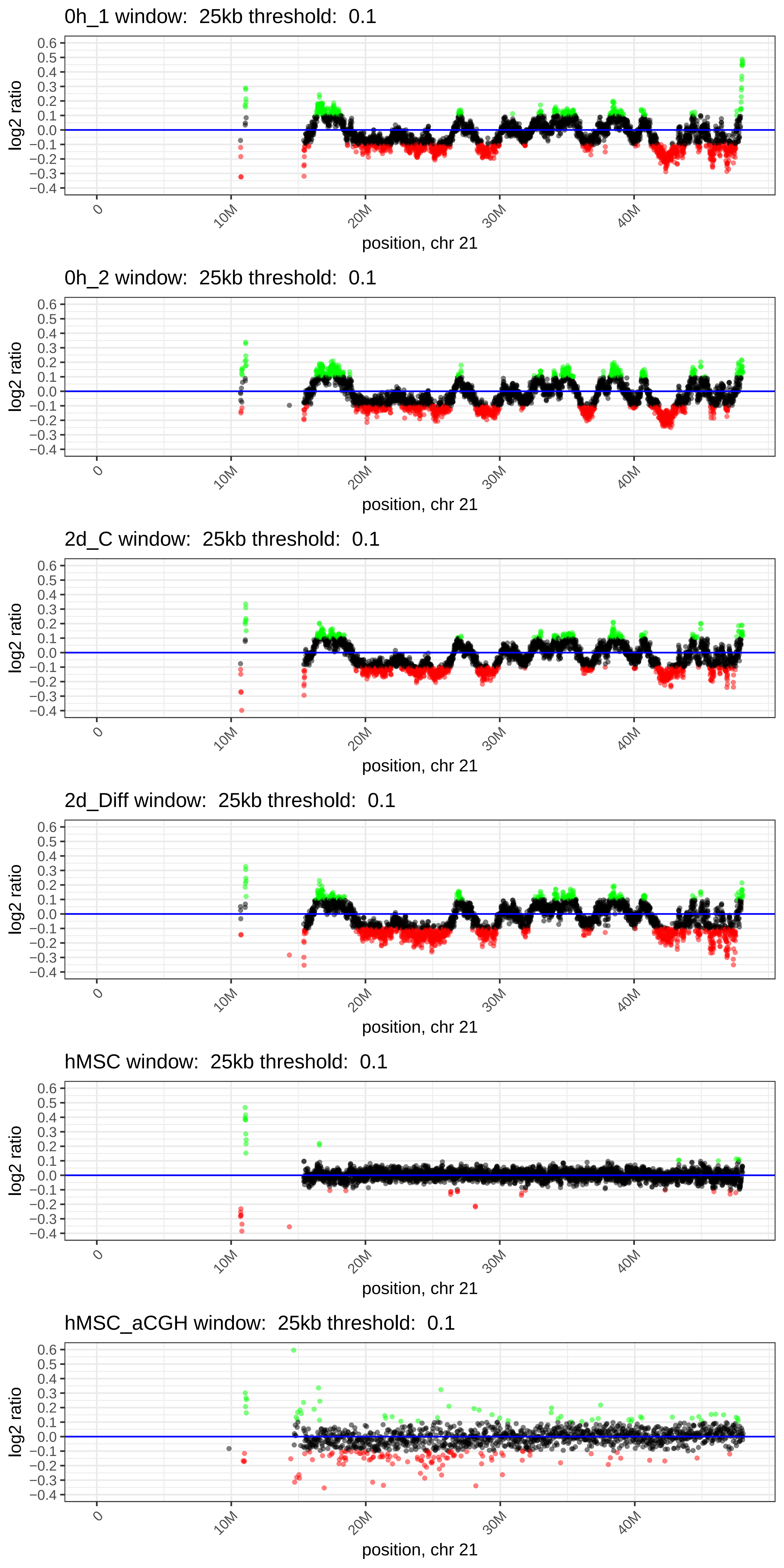

0h_1 window: 25kb threshold: 0.1
0h_2 window: 25kb threshold: 0.1
2d_C window: 25kb threshold: 0.1
2d_Diff window: 25kb threshold: 0.1
hMSC window: 25kb threshold: 0.1
hMSC_aCGH window: 25kb threshold: 0.1
log2 ratio
0.6
0.5
0.4
0.3
0.2
0.1
0.0
−0.1
−0.2
−0.3
−0.4
0
10M
20M
30M
40M
position, chr 21

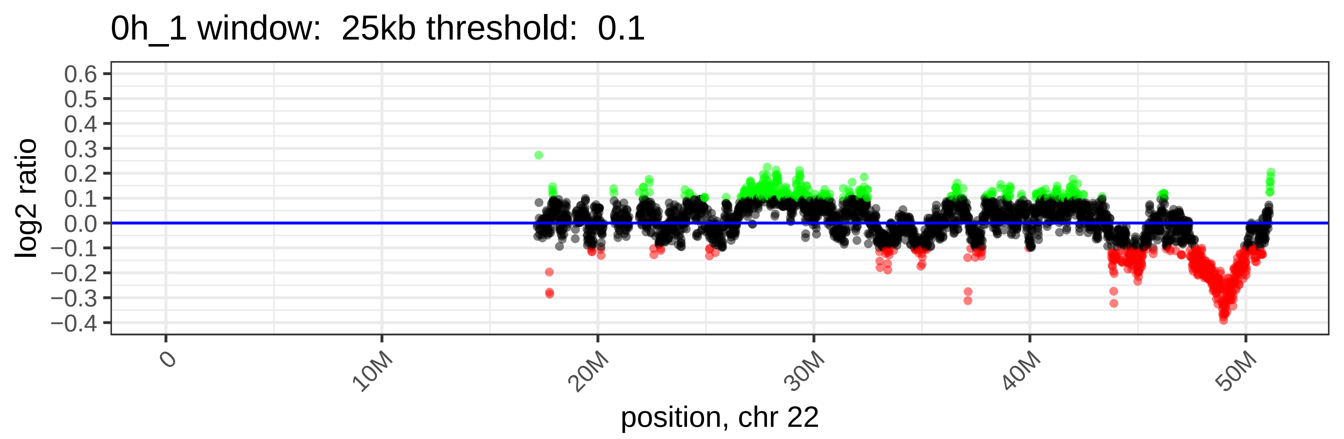
0h_1 window: 25kb threshold: 0.1
log2 ratio
0.6
0.5
0.4
0.3
0.2
0.1
0.0
−0.1
−0.2
−0.3
−0.4
0
10M
20M
30M
40M
50M
position, chr 22

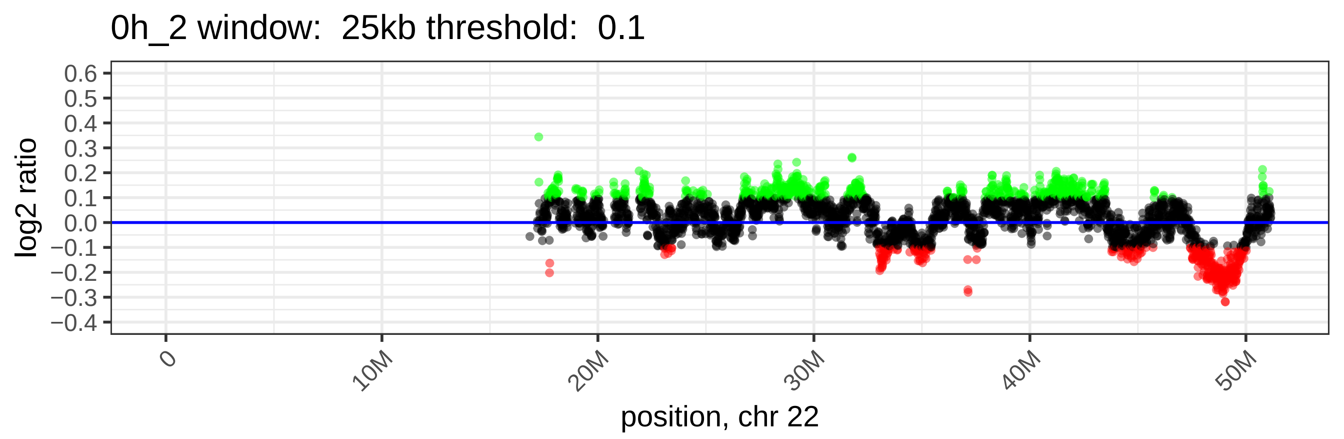
0h_2 window: 25kb threshold: 0.1
log2 ratio
0.6
0.5
0.4
0.3
0.2
0.1
0.0
−0.1
−0.2
−0.3
−0.4
0
10M
20M
30M
40M
50M
position, chr 22

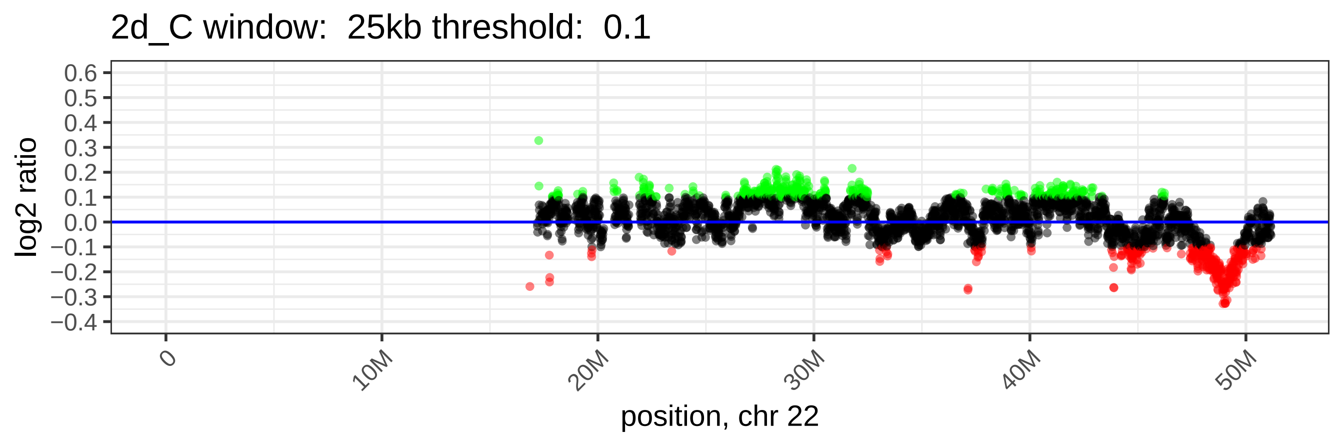
2d_C window: 25kb threshold: 0.1
log2 ratio
0.6
0.5
0.4
0.3
0.2
0.1
0.0
−0.1
−0.2
−0.3
−0.4
0
10M
20M
30M
40M
50M
position, chr 22

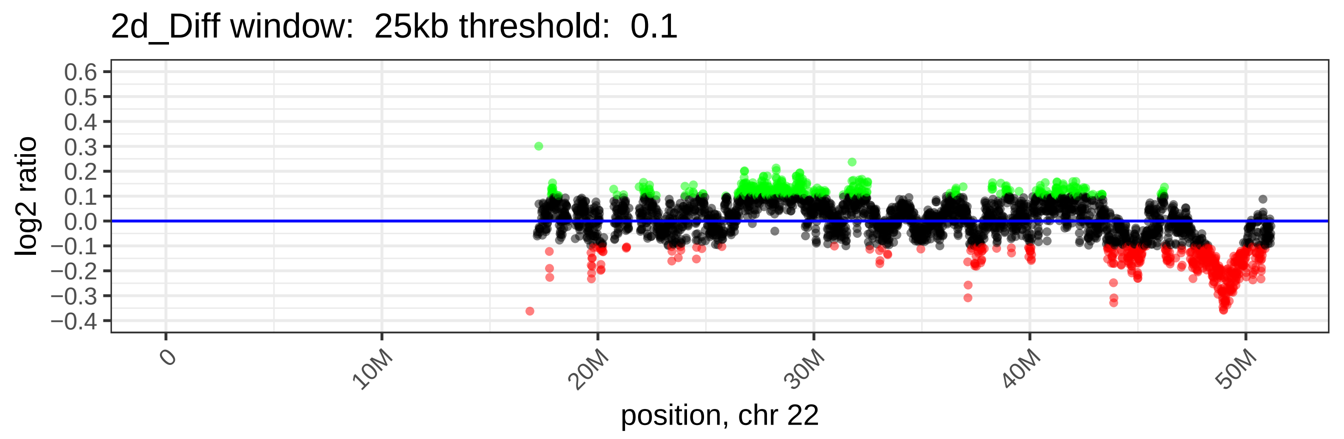
2d_Diff window: 25kb threshold: 0.1
log2 ratio
0.6
0.5
0.4
0.3
0.2
0.1
0.0
−0.1
−0.2
−0.3
−0.4
0
10M
20M
30M
40M
50M
position, chr 22

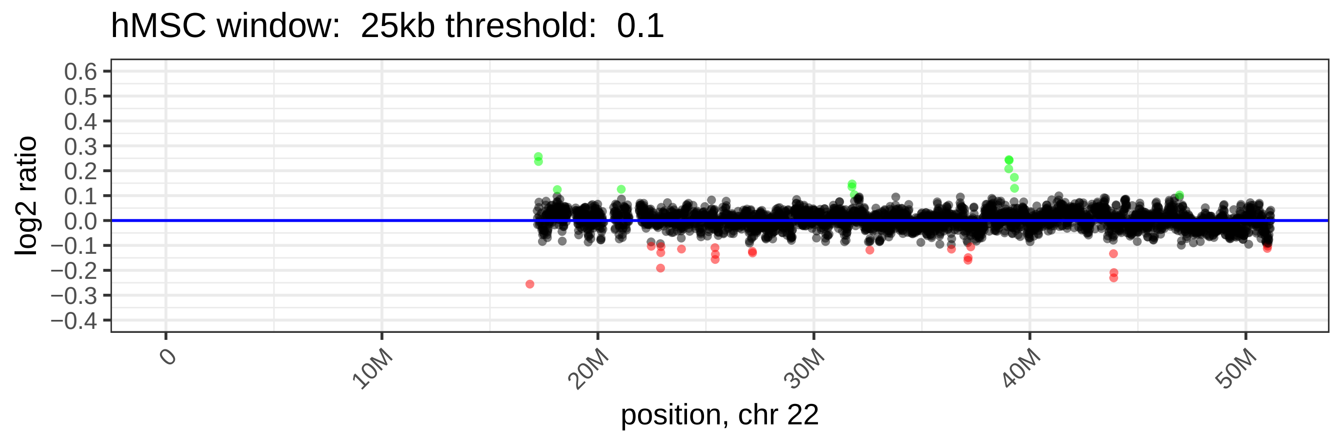
hMSC window: 25kb threshold: 0.1
log2 ratio
0.6
0.5
0.4
0.3
0.2
0.1
0.0
−0.1
−0.2
−0.3
−0.4
0
10M
20M
30M
40M
50M
position, chr 22

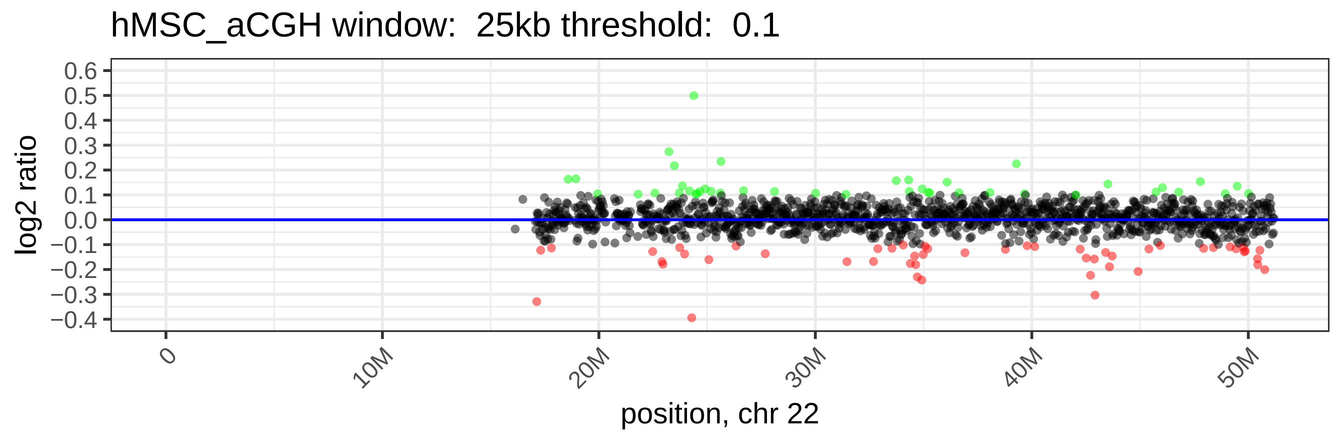
hMSC_aCGH window: 25kb threshold: 0.1
log2 ratio
0.6
0.5
0.4
0.3
0.2
0.1
0.0
−0.1
−0.2
−0.3
−0.4
0
10M
20M
30M
40M
50M
position, chr 22
